# Ginseng-derived nanoparticles alter macrophage polarization to inhibit melanoma growth

**DOI:** 10.1186/s40425-019-0817-4

**Published:** 2019-11-27

**Authors:** Meng Cao, Huaijiang Yan, Xuan Han, Ling Weng, Qin Wei, Xiaoyan Sun, Wuguang Lu, Qingyun Wei, Juan Ye, Xueting Cai, Chunping Hu, Xiaoyang Yin, Peng Cao

**Affiliations:** 1 Affiliated Hospital of Integrated Traditional Chinese and Western Medicine, Nanjing University of Chinese Medicine, Nanjing, Jiangsu China; 2 College of Pharmacy, Nanjing University of Chinese Medicine, Nanjing, Jiangsu China; 3 Collaborative Innovation Center for Cancer Medicine, Nanjing Medical University, Nanjing, Jiangsu China

**Keywords:** Extracellular vesicles (EVs), Ginseng-derived nanoparticles, Tumor-associated macrophages (TAM), Macrophage polarization, Melanoma

## Abstract

**Background:**

It is unclear whether plant-derived extracellular vesicles (EVs) can mediate interspecies communication with mammalian cells. Tumor-associated macrophages (TAMs) display a continuum of different polarization states between tumoricidal M1 phenotype and tumor-supportive M2 phenotypes, with a lower M1/M2 ratio correlating with tumor growth, angiogenesis and invasion. We investigated whether EVs from ginseng can alter M2-like polarization both in vitro and in vivo to promote cancer immunotherapy.

**Methods:**

A novel EVs-liked ginseng-derived nanoparticles (GDNPs) were isolated and characterized from *Panax ginseng* C. A. Mey. Using GDNPs as an immunopotentiator for altering M2 polarized macrophages, we analyzed associated surface markers, genes and cytokines of macrophages treated with GDNPs. Mice bearing B16F10 melanoma were treated with GDNPs therapy. Tumor growth were assessed, and TAM populations were evaluated by FACS and IF.

**Results:**

GDNPs significantly promoted the polarization of M2 to M1 phenotype and produce total reactive oxygen species, resulting in increasing apoptosis of mouse melanoma cells. GDNP-induced M1 polarization was found to depend upon Toll-like receptor (TLR)-4 and myeloid differentiation antigen 88 (MyD88)-mediated signaling. Moreover, ceramide lipids and proteins of GDNPs may play an important role in macrophage polarization via TLR4 activation. We found that GDNPs treatment significantly suppressed melanoma growth in tumor-bearing mice with increased presence of M1 macrophages detected in the tumor tissue.

**Conclusions:**

GDNPs can alter M2 polarization both in vitro and in vivo, which contributes to an antitumor response. The polarization of macrophages induced by GDNPs is largely dependent on TLR4 and MyD88 signalling. GDNPs as an immunomodulator participate in mammalian immune response and may represent a new class of nano-drugs in cancer immunotherapy.

## Background

The root of *Panax ginseng* C.A. Mey (Araliaceae) is well known for its multiple pharmacological properties, including anticancer, anti-inflammatory, antioxidant, and aging inhibitory effects [1–3]. Several studies have reported the immune-enhancing properties of ginseng root extract for cancer treatment, but the effector mechanism of their immunomodulating activity has remained partially understood [4, 5].

Extracellular vesicles (EVs) are nano-sized membrane vesicles with a cargo that includes diverse proteins, lipids, nucleic acids and polysaccharides [6, 7]. Cellular studies have shown that EVs bear surface receptors and ligands of the original cells and mediate intercellular communication [8]. In the past decade, the ability of mammalian EVs to transport bioactive contents has stimulated research into their biology and the development of EV-based therapies and diagnostic tests [9]. Like mammalian cells, plant cells also secrete EVs, although very little is known about their origins, compositions or functions [10]. Recent studies have indicated that these plant-derived nanoparticle-like EVs may be involved in plant cell–cell communication as a means to regulate plant innate immunity [11]. In addition, some plant-derived EVs may also mediate cross-species RNA interference causing fungal gene silencing [12]. It has never been reported previously whether ginseng release nanoparticle-like EVs, let alone the physiological function of plant-derived EVs in mammalian cells.

Macrophages are a major part of the mononuclear phagocyte system (MPS), which is responsible for the clearance for foreign matter from the body [13]. As a consequence, nanoparticles that come into contact with macrophages will be rapidly recognized, internalized, and degraded. This intrinsic mechanism of vesicle uptake by macrophages may be employed to target these cells for nanotherapeutic formulation [14]. There is recent evidence that natural and modified EVs from mammalian cells can induce an antitumor response in macrophages to inhibit tumor growth [15, 16]. Tumor-associated macrophages (TAMs) are a major component of the tumor microenvironment (TME) [17]. TAM infiltration in tumor tissues has been shown to support tumor growth, angiogenesis, invasion and metastasis, and a high density of TAMs in tumors is correlated with tumor progression and drug resistance. Thus, TAMs have been regarded as promising targets for novel anticancer agents [18]. In general, TAMs are considerably plastic and assume opposing phenotypes and functions, including tumoricidal M1 and tumor-supportive M2 macrophages. In most tumor types, macrophages with M2-like phenotype prevail. Thus, both depletion of M2-like cells and skewing the M1/M2 ratio towards M1-like phenotype have emerged as attractive therapeutic strategies in the treatment of cancer [19, 20].

Here, we successfully isolated and purified nanoparticle-like EVs efficiently from the roots of *Panax ginseng* C. A. Mey. Component analyses of these ginseng-derived nanoparticles (GDNPs) revealed that they are highly enriched in proteins, lipids and nucleic acids. We show that GDNPs induce M1-like macrophage polarization via Toll-like receptor (TLR)-4/myeloid differentiation antigen 88 (MyD88) signalling pathway and enhance production of total reactive oxygen species (ROS) to induce apoptosis of mouse melanoma cells. As a monotherapy, the administration of GDNPs in melanoma allografted mice altered the functional orientation of TAMs towards an M1-like phenotype, leading to suppressed tumor growth in vivo. Our work shows for the first time that GDNPs exert an immunomodulatory effect on murine macrophages to inhibit tumor growth in mice and provides the basis for further use as nano-drugs for cancer immunotherapy.

## Materials and methods

### Ethics statement, mice and cell lines

All human experimental protocols were approved by the Ethics Committee for Human Studies of Affiliated Hospital of Integrated Traditional Chinese and Western Medicine (2018LWKYZ010).

Wild-type (WT) C57BL/6 mice were purchased from the Comparative Medicine Center, Yangzhou University (Yangzhou, Jiangsu, China). MyD88-, TLR4- and TLR2-deficient C57/BL6 mice were gifts from Dr. Lixin Wang (Department of Microbiology and Immunology, Medical School of Southeast University, Nanjing, China) [21]. All animal experimental protocols were approved by the Institutional Animal Care and Use Committee of Nanjing University of Chinese Medicine.

The murine melanoma cell line (B16F10), breast cancer cell line (4T1) and human embryonic kidney cell line (HEK293T) were purchased from the Institute of Biochemistry and Cell Biology, Academy of Science (Shanghai, China). Cells were cultured in Dulbecco’s Modified Eagle’s Medium (DMEM) or RPMI 1640, supplemented with 10% foetal bovine serum (FBS), 100 U/ml penicillin, and 100 mg/ml streptomycin (all from Thermo Fisher Scientific, USA). All cells were incubated at 37 °C in a humidified atmosphere with 5% CO_2_.

### Purification and characterization of GDNPs

For isolation of GDNPs, fresh ginseng roots were purchased from *Panax ginseng* Base (Wanshan, Jilin, China) and washed with deionized water three times at room temperature (20 °C). After the final wash, ginseng roots were ground in a slow juicer to obtain ginseng fluid. Then, the juice was sequentially centrifuged at 200×*g* for 10 min, 2000×*g* for 20 min and 10,000×*g* for 30 min to remove large particles and fibres. The final supernatant was ultracentrifuged at 100,000×*g* for 60 min (Beckman Optima XE-100, Beckman, USA), and the pellets were resuspended in PBS, transferred to a gradient sucrose solution (15, 30, 45 and 60%) and ultracentrifuged at 150,000×*g* for another 60 min. The band at the 45% sucrose layer was collected and defined as GDNPs according to TEM (transmission electron microscopy) examination [22]. Finally, the GDNPs were diluted in PBS and ultracentrifuged at 100,000×g for 60 min, then the pellets were resuspended in sterile PBS. The resuspension was filtered (0.45 μm) and used freshly or stored at − 80 °C until further use.

The size and zeta potential of GDNPs were measured by dynamic light scattering using a Zetasizer nano ZS Zen3600 (Malvern, UK). For TEM imaging, a drop of purified GDNPs was deposited onto the surface of formvar-coated copper grids, followed by incubation with 1% uranyl acetate for 15 s. The samples were left to dry at room temperature and observed using a HITACHI H-7650 electron microscope operated at 200 kV at a magnification of 38,000×. The protein concentration in the GDNPs was quantified using a BCA protein assay kit (Beyotime Biotechnology, China) following the manufacturer’s instructions.

### Human PBMC derived macrophages and mouse bone marrow-derived macrophages (BMDMs) preparation and polarization

Human peripheral blood mononuclear cells (PBMCs) were collected from venous blood of healthy volunteers, and in leukocyte reduction chambers, diluted 2x with PBS and separated via Ficoll density gradient (Serumwerk Bernburg AG, Norway). CD14^+^ monocytes were positively selected to > 95% purity by MACS using anti-CD14 microbeads (Miltenyi, USA). Mouse bone marrow was collected by flushing the femurs of C57BL/6 mice (8~10 weeks old) with cold PBS. After collection, red blood cells were lysed with RBC lysis buffer (Thermo Fisher Scientific, USA), and the remaining cells were washed twice with PBS. For induction of macrophage differentiation, sorted monocytes or bone-marrow cells were cultured in RPMI 1640 or DMEM supplemented with 10% FBS and 20 ng/ml human or mouse macrophage colony-stimulating factor (M-CSF) (R&D Systems, USA). Fresh medium with M-CSF was added every 3 days. On day 7, M2-like polarization was achieved by treatment with human/mouse 20 ng/ml IL-4 and 20 ng/ml IL-13 (R&D Systems, USA) for 2 days. For M0, only DMEM-10% FBS was added. After polarization, the cells were phenotyped and used in different assays. The media from M0, M2 and GDNP-treated M2 macrophages was collected for ELISA, cytokines array and apoptosis assays.

For analysing the role of TLR signalling pathways in macrophage polarization induced by GDNPs, M2-like macrophages were purified from wild type, MyD88-, TLR4- and TLR2-deficient C57/BL6 mice and incubated with GDNPs for 72 h. The supernatants were collected for detection of IL-6 and tumor-necrosis factor-α (TNF-α) using ELISA kits (R&D Systems, USA). Then, the cells were harvested for detection of associated macrophage surface markers by flow cytometry analysis.

### Analysis of the uptake efficiency of GDNPs by macrophages

Cells (M0 macrophage, B16F10, 4T1 and HEK293T) were seeded on 12-chamber slides (Thermo Fisher Scientific, USA) at a density of 5 × 10^5^/well and cultured overnight at 37 °C. The media was then replaced with fresh culture media containing GDNPs (10 μg/ml) previously labelled with DiI (Thermo Fisher Scientific, USA) according to the manufacturer’s protocol. After 12 h incubation, the cells were fixed with 4% paraformaldehyde for 10 min and then dehydrated with acetone at − 20 °C for 5 min. After the cells were blocked with anti-CD16/32 (Fc block, BioLegend, USA) in PBS for 30 min, antifade mountant with 4′,6-Diamidino-2-Phenylindole (DAPI) (Thermo Fisher Scientific, USA) was added, and the mixture was incubated for an additional 30 min. Finally, cells were coverslip-mounted with mounting medium and imaged using an Olympus FV10i confocal microscope with Olympus Fluoview software version 4.0b (Olympus, Japan).

To determine the efficiency of GDNP uptake by macrophages, cells (1 × 10^6^/well) were co-incubated with DiI-labelled GDNPs (10 μg/ml) for 12 h or 24 h. The cells were harvested and single cell suspensions were prepared and analysed using the FACSAria II system (BD Biosciences, USA). Data analysis was performed using FlowJo Version7.6 (BD Biosciences, USA).

### Biodistribution and stability assays in vivo

For analysis of biodistribution of GDNPs in vivo, healthy male C57BL/6 mice (6–8 weeks old) were intraperitoneally (i.p.), intragastrically (i.g.), intravenously (i.v.) and subcutaneously (s.c.) administrated DiR-labeled GDNPs. Mice were sacrificed and different organs were collected at 72 h after the injection. The intensity of DiR signal from different samples was then measured using IVIS series in vivo imaging systems (PerKinElmer, USA). In vivo stability of DiR dye-labelled GDNPs was determined by scanning (IVIS series) mice that received an i.p. injection of DiR-GDNPs for 7 days.

To study GDNPs taken up by macrophages in vivo, healthy male C57BL/6 mice (6–8 weeks old) were received i.p. injection with clodronate liposomes (CL, 200 μg per mouse) [23], which deplete macrophages (*n* = 3). After 3 days, mice were i.p. administrated with DiR-labeled GDNPs in the presence or absence of CL. Mice were sacrificed and organs were collected at 72 h after the injection. The intensity of DiR signal of livers and spleens from mice treated with DiR-labeled GDNPs was then measured using IVIS series. In addition, splenocytes from mice treated with DiI-labeled GDNPs for 72 h were obtained by gently pressing the spleens between two sterile glass slides followed by washing the lymphocytes with PBS. Single cell suspensions were prepared by filtering through a 100-μm nylon filter strainer and washed thoroughly in PBS. Nonspecific labelling was blocked with anti-CD16/32 followed by staining with the following mouse monoclonal antibodies (BioLegend, USA and Thermo Fisher Scientific, USA) to detect splenocytes surface markers: anti-CD45 APC; anti-CD45 Brilliant Violet 510; anti-CD3 FITC; anti-CD4 PE/Cy7; anti-CD335 PerCP/Cy5.5; anti-CD11b APC/Cy7; anti-F4/80 PE/Cy7; anti-Ly6C/6G APC; anti CD11c FITC; and anti-CD45R/B220 Brilliant Violet 510, according to the manufacturer’s instruction (Additional file 1: Table S1). The stained cells were analysed on a FACSAria II Flow Cytometer using BD FACSDiva software (BD Biosciences, USA), and the data were processed using FlowJo Version 7.6 (BD Biosciences, USA).

### Measurement of GDNPs inhibition of M2-like macrophage polarization in vitro

M2-like macrophages (1 × 10^6^/well) were incubated with or without GDNPs (10 μg/ml). At 48 h, supernatants were collected for detection of M1-associated cytokines, including IL-6 and TNF-α, using enzyme-linked immunosorbent assay (ELISA) kits according to the manufacturer’s protocol (R&D Systems, USA). Then, the cells were harvested for measurement of gene expression and detection of surface markers as described below.

Total RNA was isolated from treated macrophages using TRIzol reagent (TaKaRa, Japan) and reverse transcribed into cDNA using a cDNA Synthesis Kit (TaKaRa, Japan) according to the manufacturer’s protocol. Then, RT-PCR was performed using SYBR Green Mastermix (Toyobo, Japan) following the manufacturer’s instructions and run on an ABI Prism 7500 Sequence Detection System (Applied Biosystems, USA). The primer sequences are shown in Additional file 1: Table S2. The 2^ΔΔCt^ method was used to calculate fold changes in gene expression normalized to the housekeeping gene *Gapdh* (Additional file 1: Table S2).

Single-cell suspensions of treated macrophages were prepared in PBS. Nonspecific labelling was blocked with anti-CD16/32 followed by staining with the following mouse monoclonal antibodies (BioLegend, USA and Thermo Fisher Scientific, USA) to detect macrophage surface markers: anti-CD206 APC; anti-CD80 APC; anti-CD86 PE; anti-MHC-II FITC; anti-CD11b APC; anti-F4/80 PE; anti-TLR2 FITC; and anti-TLR4 PE/Cy7, according to the manufacturer’s instruction. For each sample at least 2 × 10^4^ cells were analysed by flow cytometry. Data analysis was performed using FlowJo software (BD Biosciences, USA).

### Analysis of the GDNPs components involved in macrophage polarization

For lipidomic analysis, lipids from GDNPs were submitted to the APTBIO Co. Ltd. (Shanghai, China). Briefly, high-throughput identification and relative quantification of lipids was performed separately for positive and negative ionization mode data using LipidSearch software (Thermo Fisher Scientific, USA) using the default parameters for QExactive as previously described [24]. Data for each lipid molecular species were presented as mol % of the total lipids analyzed.

GDNPs were digested with proteinase K or DNase I/RNase I according to the manufacturer’s instructions (Beyotime Institute of Biotechnology, China). After digestion with proteinase K or DNase I/RNase I, proteins or DNA in supernatant were analyzed via 2% SDS polyacrylamide gel electrophoresis or agarose gel electrophoresis. The effect of protein- and nucleic acid-depleted GDNPs on macrophage polarization was analyzed as described above.

### Animal experiments

On day 0 of the experiments, sixteen C57BL/6 male mice (6–8 weeks old) were subcutaneously inoculated with 2 × 10^5^ B16F10 cells on their right flanks. The tumor size was measured every 2 days using digital calipers and tumor volume was calculated using the following equation: V = (length×width^2^)/2. Body weight was also monitored every two days. On day 7 post-tumor implantation, tumor-bearing mice were randomly separated into two groups (8 mice per group) that received four intraperitoneal injections (100 μl per mouse) in total, administered every four days, containing the following formulations: PBS and GDNPs (250 μg per mouse). Tumor growth was monitored for up to 21 days after implantation, at which point animals were euthanized in a CO_2_ chamber and tumors were removed for further analysis. All tumors were divided in two pieces, weighed and processed for both flow cytometry and histopathology as described below.

To investigate tumor growth inhibition in our mouse melanoma model was driven by GDNP-mediated TAMs polarization, B16F10-bearing C57BL/6 mice were treated with GDNPs in the presence or absence of clodronate liposomes. CL treatment (200 μg per mouse) was repeated every 4 days by i.p. injection. Mice in the control group (*n* = 5) were treated with the same dose of liposomes containing PBS at the same time. The tumor size was measured and mice were treated with GDNPs as described above. Twenty-one days after implantation, tumors were processed for IF.

### Tissue dissociation and flow cytometry

Tumor samples were minced with scissors before incubation with 66 μg/ml Liberase and 0.2 mg/ml DNase (Roche, Switzerland) in DMEM for 30 min at 37 °C. Single cell suspensions were prepared by filtering through a 100-μm nylon filter strainer and washed thoroughly in Hank’s balanced salt solution (HBSS) buffer supplemented with 2% FBS, 20 mM HEPES, and 5 mM EDTA. Fixable Viability Dye (Thermo Fisher Scientific, USA) was applied to eliminate dead cells in combination with anti-CD16/32 monoclonal antibody (BioLegend, USA) for 15 min on ice in the dark. Then, cells were stained for 30 min in PBS with appropriate dilutions of various combinations of the following fluorochrome-conjugated antibodies: anti-CD206 PE; anti-CD86 APC/Cy7; anti-CD45 APC; anti-CD11b FITC; and anti-F4/80 PE/Cy7; anti-CD4 PE/Cy7; anti-CD3 APC/Cy7; anti-CD25 APC; anti-CD8 FITC; anti-NK1.1 PE; anti-CD45 Brilliant Violet 510; anti-CD45R Brilliant Violet 510; anti-CD335 PerCP/Cy5.5; anti-CD45 Brilliant Violet 421; anti-Ly6G Alexa Fluor® 647; anti-CD11c FITC. For intracellular staining, cells were further permeabilized using a FoxP3 Fixation and Permeabilization Kit (Thermo Fisher Scientific, USA) and stained with anti-FoxP3 PE antibody (Thermo Fisher Scientific, USA). All flow cytometry data was acquired and analyzed as mentioned above.

### Statistical analysis

The results are expressed as the mean ± standard error (S.E.M). All data were analyzed using GraphPad Prism 6.0 (GraphPad Software, USA) by unpaired Student’s *t*-test and two-way analysis of variance (ANOVA). *P* < 0.05 was considered statistically significant (*P < 0.05, **P < 0.01, ***P < 0.001, ****P < 0.0001).

## Results

### Generation, isolation and characterization of ginseng-derived nanoparticles (GDNPs)

**Fig. 1:**
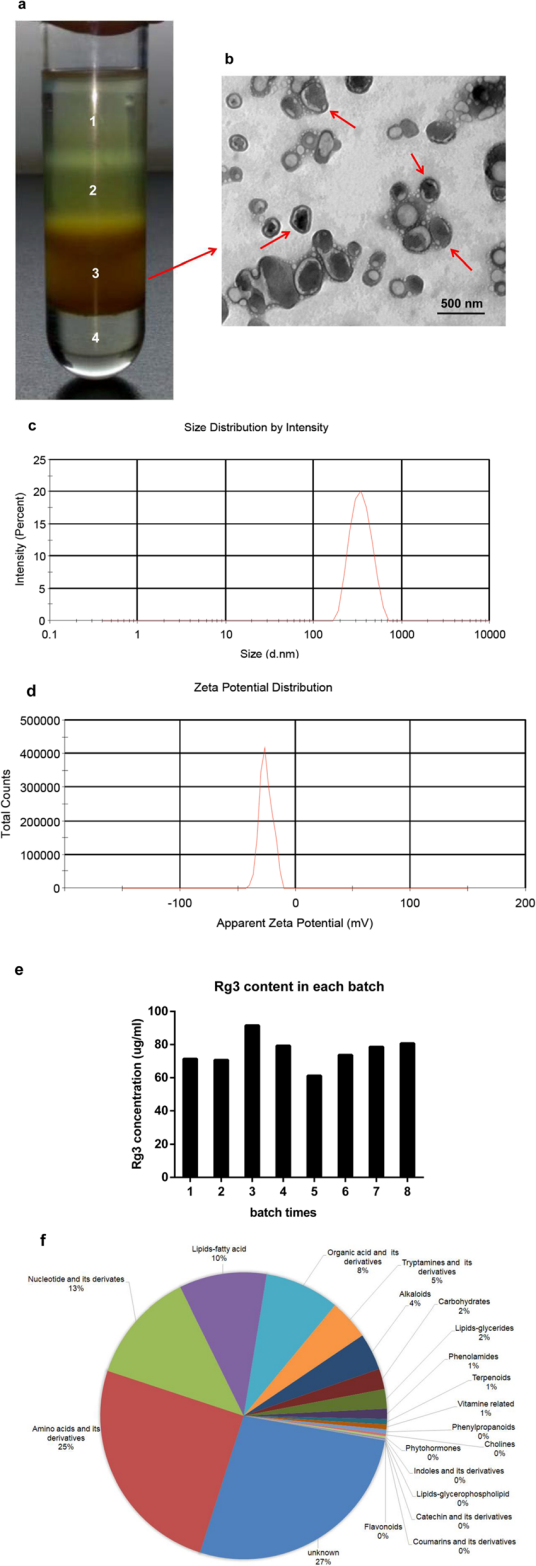
Characterization of ginseng-derived nanoparticles (GDNPs) prepared from ginseng roots. **a** Ginseng root juice was purified by sucrose density gradient (15%/30%/45%/60%) under ultracentrifugation, and the band from the interface of 45% (band 3) was harvested and defined as GDNPs according to the literature for further use. **b** GDNPs harvested from the sucrose density gradient (45%) were characterized by transmission electron microscopy (TEM) (Scale bar = 500 nm). **c** Particle size of the GDNPs was measured by dynamic light scattering (DLS). **d** Representative chart of the surface charge of GDNPs determined by dynamic light scattering coupled with Laser Doppler Velocimetry. **e** Ginsenoside Rg3 contents of GDNPs in each batch. **f** Pie chart of the composition of GDNPs showing the percentage of total metabolites. The composition for each protein and metabolite molecular species is reported as % of total proteins and metabolites analysed. For each category, a two-tailed Fisher’s exact test was applied to test the enrichment of the differentially expressed protein against all identified proteins. These analyses are derived from two biological replicates

To isolate EVs from ginseng, ginseng roots were harvested, followed by a combination of extraction, filtration and differential centrifugation. Four bands were formed after sucrose gradient ultracentrifugation. Transmission electron microscopy (TEM) examination indicated the majority of the GDNPs accumulated at the 45% interface (band 3), generally spherical in shape (Fig. 1a and b). Purified GDNPs were identified with an average diameter (as determined by dynamic light scattering) of ~ 344.8 nm (band 3) and a low polydispersity (Fig. 1c). Zeta potential analysis indicated that GDNPs had a negative zeta potential value of − 25.4 mV (Fig. 1d). The GDNPs were quantified by protein concentration using a micro BCA protein assay kit. Extracts from ginseng roots are enriched for nanoparticles (approximately 500 mg/kg ginseng), suggesting that ginseng could produce a large amount of GDNPs. In addition, the ginsenoside Rg3 were detected highly concentrated in the GDNPs by electrospray spray ionization (ESI) scanning. Our data indicated that the concentrations of the ginsenoside Rg3 were similar within different batches (Fig. 1e). It is a potential component to control quantities of GDNPs within different batches.

Next, we analysed the composition of the purified GDNPs using mass spectrometry (MS) in duplicate. Metabolic analysis revealed that GDNPs contained amino acids (~ 25%), nucleotides (~ 13%), lipids/fatty acids (10%) and organic acids (8%) (Fig. 1f). In addition, we identified 3129 proteins that were classified using Gene Ontology (GO) analysis into three categories: biological process, cellular compartment and molecular function (Additional file 2: Figure S1a-c).

### Internalization of GDNPs by mouse macrophages in vitro

**Fig. 2:**
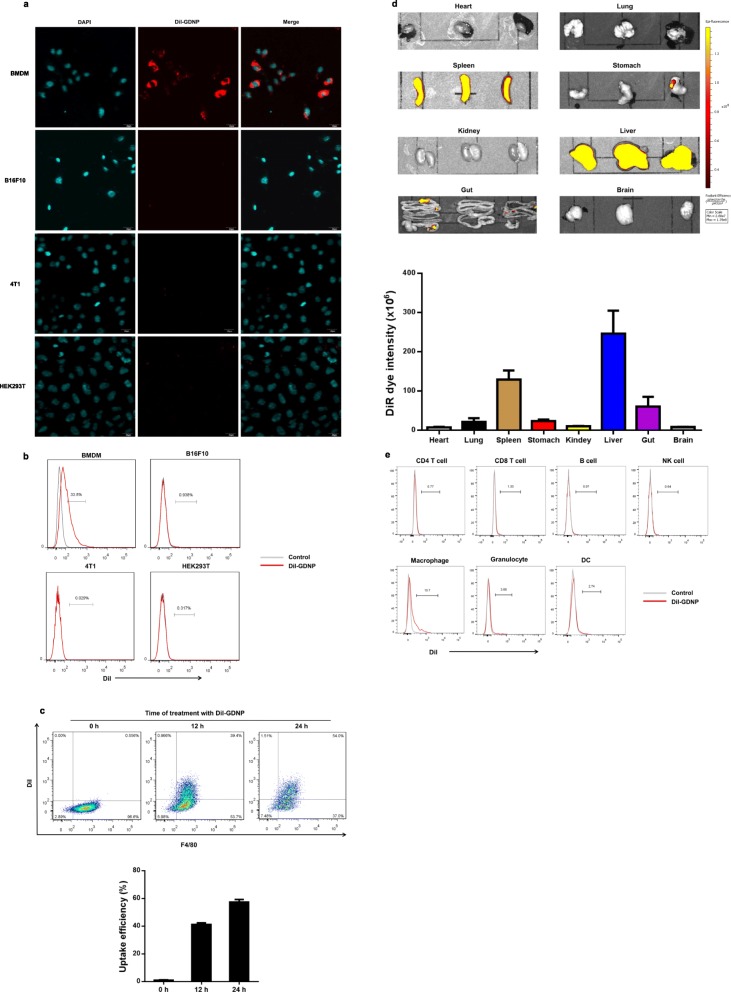
GDNPs are efficiently taken up by macrophages. **a** Confocal images (top) and FACS quantitative analysis (bottom) of DiI-labelled GDNPs (10 μg/ml) taken up by BMDM, B16F10, CT26 and HEK293T cells. Cells were incubated with DiI-labelled GDNPs for 12 h (Scale bar = 20 μm). **b** Quantitative flow cytometry analysis of DiI-labelled GDNPs taken up by BMDM (F4/80-FITC) at different time points. **c** Uptake efficiency was quantified by flow cytometry (*n* = 4). **d** In vivo biodistribution of GDNPs was determined by scanning mice that received an i.p. injection of DiR-labelled GDNPs. The main organs of the treated mice were examined. **e** In vivo FACS quantitative analysis of spleen cells uptake of DiI-labelled GDNPs administrated i.p. injection

Previous reports have shown that ginseng polysaccharide extracts stimulate the activity of macrophages and enhance the production of different mediators or active components [4, 5]. Since macrophages exhibited a high uptake potential for nanoparticles, we tested whether GDNPs are taken up by macrophages in vitro. BMDM, B16F10, 4T1 and HEK293T were incubated with GDNPs labelled with DiI, a lipophilic fluorescent dye, for 12 h. Compared to other cells, we found that GDNPs (red) were taken up more effectively by BMDMs and preferentially localized in the cytoplasm of the cells (Fig. 2a). Flow cytometry showed that the percentage of cells containing GDNPs increased with time from 41.3% at 12 h to 57.4% at 24 h (Fig. 2b and c).

### Biodistribution, stability and biocompatibility of GDNPs

To determine in vivo biodistribution of GDNPs, we first evaluated the effect of different routes of administration of DiR-labelled GDNPs. At 72 h following i. p. and i.v. injection, the majority of the DiR-labelled GDNPs were located in the liver and spleen, whereas i.g. administration of DiR-labelled GDNPs were predominantly localized in stomach and gut when compared with PBS-treated control mice. However, no signal was detected in lung, heart, kidney and brain (Fig. 2d; Additional file 2: Figure S2a and b). In vivo imaging to continuously track the stability of injected DiR-GDNPs further revealed that fluorescent signals remained strong in the liver and spleen at day 7 (Additional file 2: Figure S2c). Our findings suggested that the size and structure of nanoparticulate increased the stability and retention of GDNPs in the circulation.

FACS analysis was done on splenic cells from mice received i.p. injection of DiI- GDNPs. The results indicated that 72 h after GDNPs were i.p. administration, they were easily taken up by macrophages (13.7%) (Fig. 2e). Upon analysis of the biodistribution of DiR-GDNPs with i.p. injection in the presence of CL, we found that the DiR fluorescent signals decreased significantly in the liver and spleen (Additional file 2: Figure S3a and b). Our findings showed the cellular tropism of GDNPs to macrophages in vitro and in vivo.

To assess the biocompatibility of GDNPs in vitro, cell viability assays were performed. The results of cell viability assays revealed that GDNPs exhibited no cytotoxicity on cells for 72 h, even at the high concentration of 30 μg/mL (Additional file 2: Figure S4a). To further evaluate the biosafety of GDNPs in vivo, mice were treated with GDNPs via i.p. injection. Sex-, age- and weight-matched healthy mice were used as controls. The body weights of the mice from GDNPs treated groups did not differ significantly from those of the control group (Additional file 2: Figure S4b). Two weeks after injection, all mice were euthanized for blood biochemistry and hematology analyses as well as histological examinations. As shown in Additional file 2: Figure S4c and d, i.p. injection of GDNPs did not lead to any changes in blood cells, hemoglobin, and platelets. No statistically significant differences were detected by evaluating liver enzymes, kidney function and hematologic toxicity. In addition, the heart, lungs, liver, spleen and kidneys were collected for haematoxylin & eosin (H&E) staining (Additional file 2: Figure S4e). No apparent organ or tissue damages in the brain, heart, kidney, liver, lungs or spleen were observed in GDNP-administrated mice, compared with those in control group. Thus, these results indicate that GDNPs show no significant toxic effects in the range of administration in vitro and in vivo.

### GDNPs alter M2-like polarization of macrophages in vitro

**Fig. 3:**
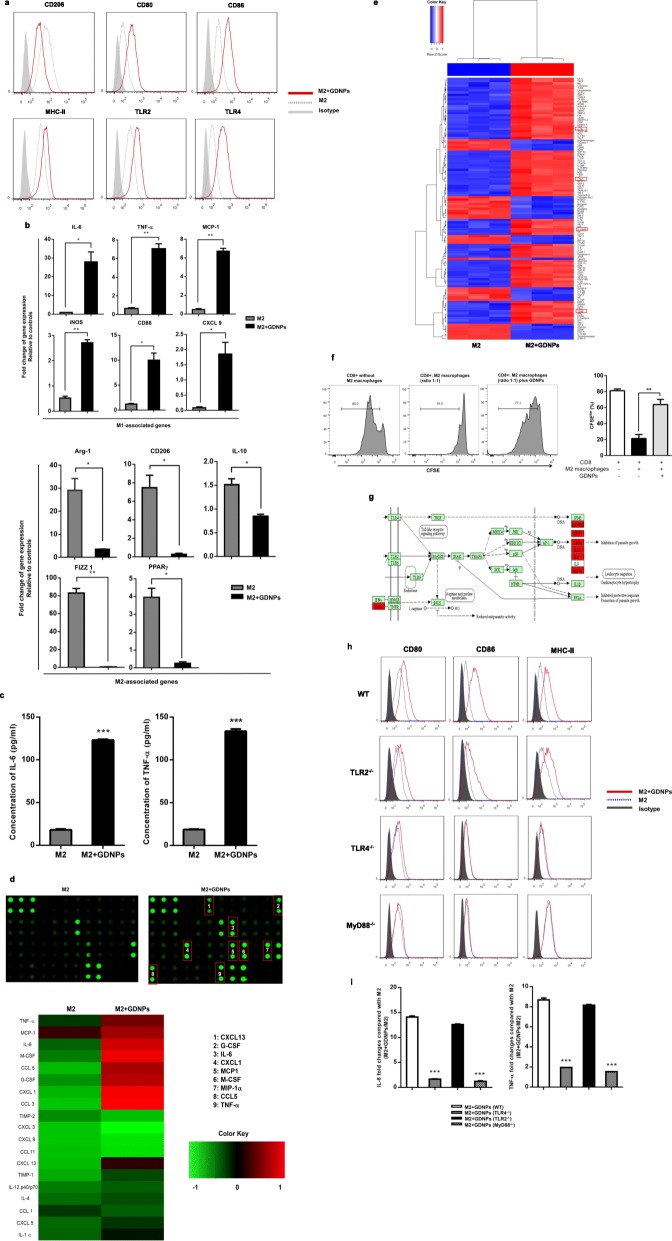
GDNPs inhibit M2-like polarization of macrophages. BMDM macrophages were M2 polarized in vitro by treatment with 20 ng/ml IL-4 and 20 ng/ml IL-13 for 2 days. **a** Representative flow cytometry data showing the surface marker expression profile of M2 macrophages treated with or without GDNPs (10 μg/ml) for 48 h. The shadowed area represents isotype staining. **b** Quantitative RT-PCR was carried out to assess mRNA expression of M1-marker genes and M2-marker genes. **c** IL-6 and TNF-α in the supernatants were analysed by ELISAs. **d** Heat map analysis of inflammatory cytokines from murine M2 macrophages in the presence or absence of GDNPs. **e** Heat map analysis of inflammatory cytokines from human M2 macrophages in the presence or absence of GDNPs. **f** In vitro suppressive activity of M2 macrophages treatment with GDNPs or PBS. Representative histograms of CD8^+^ T cell proliferation at a ratio of 1:1 CD8^+^ to M2 cells (left panel) and quantification of CD8^+^ T cell proliferation using CFSE dilution (right panel). **g** Signalling pathway analysis based on cytokine array examination (high expression of cytokines from murine M2-like macrophages with GDNPs treatment are marked with red). **h** M2-like macrophages were prepared from wild-type (WT), TLR2^−/−^, TLR4^−/−^ or MyD88^−/−^ mice and cultured with or without GDNPs (10 μg/ml) for 48 h. The expression of surface markers on macrophages was analysed by flow cytometry. The shadowed area represents isotype staining. **i** IL-6 and TNF-α were measured in the supernatants by ELISAs. The results represent three independent experiments as the mean ± SEM. *P < 0.05, **P < 0.01, ***P < 0.001 compared with M2 (b, c, f) or M2 + GDNPs (WT) (i); evaluated using Student’s *t* test

M2-like macrophages account for the majority of TAMs. Thus, inhibiting or altering M2-like cells is considered to be an effective therapeutic strategy in cancer therapy. Next, we determined whether GDNPs can alter M2-like polarization of macrophages. To this end, we incubated BMDMs with IL-4 and IL-13 for 24 h to polarize the cells to an M2-like phenotype, and then added GDNPs (10 μg/ml) for 48 h. Polarization corroboration by flow cytometric analysis was performed to examine the levels of polarization-related surface markers CD80^mid^, CD86^mid^, MHC-II(I-A^b^)^mid^, TLR2/4^mid^, and CD206^high^, characteristic of M1/M2 macrophages [25–27]. Treatment with GDNPs resulted in significantly reduced levels of CD206 in M2-like macrophages, whereas the expression of CD80, CD86, MHC-II and TLR2/4 was up-regulated (Fig. 3a).

To further confirm that treatment with GDNPs alter M2-like polarization, we prepared RNA samples from M1 and M2 macrophages and measured the expression of M1- and M2-associated genes using quantitative real-time polymerase chain reaction (RT-PCR). Transcriptional profiling revealed that GDNP exposure significantly induced M1-related markers while M2-associated markers were down-regulated (Fig. 3b). Increased production of the M1 markers, IL-6 and TNF-α, in the medium of GDNP-treated macrophages was further verified by ELISA (Fig. 3c).

Moreover, production of inflammatory cytokine by M2-like macrophages before and after GDNP treatment was quantified in the macrophage medium using a cytokine array. The results showed that treatment with GDNPs resulted in a dramatic increase in the production of M1-related cytokines and chemokines, such as CCL5, IL-6, MCP-1, TNF-α IL-1α and IL-12 (Fig. 3d). As shown in the heat maps of human cytokines microarray analysis, the similar results were observed (Fig. 3e). The proinflammatory cytokine IL-12 and TNF-α are known to promote cell-mediated immunity via stimulation of Th1 immune response. Thus, we tested the suppressive function of M2 macrophages with GDNPs treatment on naive CD8^+^ T cell proliferation. The results showed that suppression of CD8^+^ T cells was mitigated when GDNPs were added to the M2 macrophages (Fig. 3f). Collectively, these data revealed that GDNPs effectively inhibit M2-like polarization of macrophages in vitro.

### GDNPs induce macrophage polarization via a TLR4-MyD88-dependent mechanism

In innate immunity, macrophages produce proinflammatory mediators upon activation of several receptors that recognize pathogens, including the family of Toll-like receptors (TLRs) [28–30]. Based on cytokine array examination, the signalling pathway analysis suggested that the response induced by GDNPs on macrophages is similar in TLRs/MyD88 (myeloid differentiation antigen 88) signalling pathway induced by pathogen-associated molecular patterns (PAMPs) (Fig. 3g). We hypothesized that the immunomodulatory effect of GDNPs might occur via a similar signalling pathway. To examine this hypothesis, we generated M2-like macrophages from mice deficient in MyD88, a common signalling adaptor for different TLRs [31]. Up-regulation of M1-related surface markers and production of the cytokines IL-6 or TNF-α did not occur when MyD88^−/−^ M2-like macrophages were incubated with GDNPs (10 μg/ml) for 48 h (Fig. 3h). To determine which TLRs were specifically responsible for the GDNP-induced M1-like macrophages associated cytokines production, the response to GDNP treatment was analysed in M2-like macrophages derived from mice lacking TLR2 or TLR4. We found that TLR2^−/−^ M2-like macrophages produced those cytokines in response to GDNPs but TLR4^−/−^ M2-like macrophages failed to do so (Fig. 3i). These findings suggest that TLR4 on macrophages may interact with ligands on GDNPs, resulting in macrophage polarization.

For evaluating whether the EVs-like nanoparticles from non-medicinal plant have the similar effect of macrophages polarization, EVs-like nanoparticles from cucumber (*Cucumis sativus* L.) and kiwi (*Actinidia chinensis*) were isolated. BMDMs were incubated with nanoparticles (10 μg/ml) derived from ginseng (GDNPs), cucumber (CDNPs) and kiwi fruit (KDNPs) for 48 h. Polarization corroboration by FACS analysis was performed to examine the levels of M1-related surface markers. These results showed that nanoparticles from cucumber and kiwi fruit could not polarize macrophages to M1 type (Additional file 2: Figure S5).

### GDNP-treated macrophages inhibit melanoma growth in vitro

**Fig. 4:**
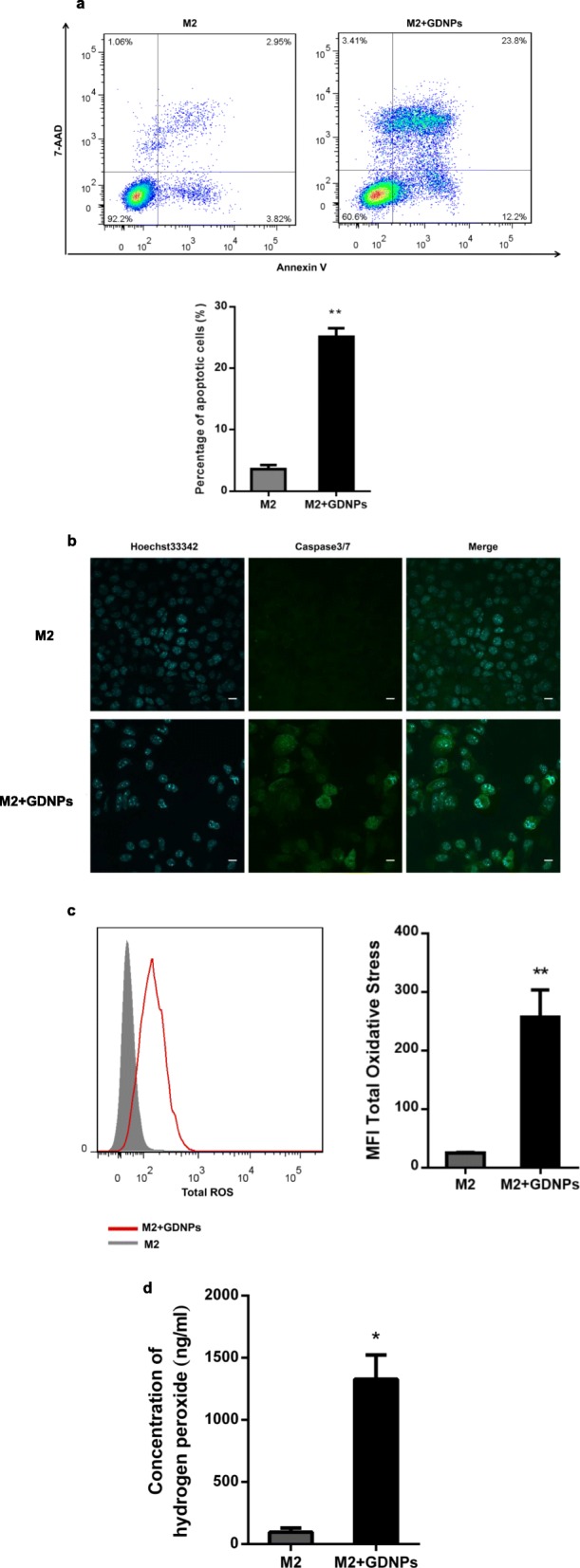
GDNPs inhibit M2 macrophage-promoted tumor cell growth in vitro. **a** B16F10 cells were cultured in the presence of conditioned medium prepared from M2 macrophages treated or untreated with GDNPs for 24 h, and apoptosis was evaluated by FACS using Annexin V-PE/7-AAD staining. Corresponding quantitative data of the percentages of apoptotic cells is shown below. **b** B16F10 cells in the abovementioned cultures were stained for caspase 3/7 expression (green). The cell nuclei were counterstained with Hoechst33342 (blue) (Scale bar = 20 μm). **c** ROS production was measured in M2-like macrophages treated with or without GDNPs (10 μg/ml) for 48 h by FACS (left). Quantitative data is shown in the graph on the right. **d** Hydrogen peroxide was quantified in the medium from M2 macrophages treated with or without GDNPs with a colorimetric hydrogen peroxide detection kit. The results represent three independent experiments as the mean ± SEM. *P < 0.05, **P < 0.01 compared with M2; evaluated using Student’s *t* test (**a**, **c**, **d**)

Since M1-like macrophages are actually capable of killing tumor cells by producing proinflammatory cytokines, promoting T helper type 1 cell response and releasing ROS [26, 32]. We investigated the effect of GDNPs on the macrophage-tumor cell interaction. M2-like macrophages were treated with or without GDNPs (10 μg/ml), and the culture medium was replaced with fresh medium. After further 48 h, the supernatant medium was collected as conditioned medium (CM). To quantitatively assess the apoptotic effects of different CM, we treated B16F10 melanoma cells with the different CM for 24 h and stained them with an annexin V-PE/7-AAD apoptosis assay detection kit. We found that treatment with CM from GDNP-stimulated macrophages significantly increased apoptosis of B16F10 melanoma cells compared to treatment with CM from unstimulated macrophages as measured by increased Annexin V binding (Fig. 4a) and increased caspase 3/7 expression (Fig. 4b).

Previous reports have demonstrated that production of total ROS and superoxide levels is increased in tumoricidal M1-like macrophages, resulting in production of highly toxic hydrogen peroxide through TLR-mediated signalling [33]. Therefore, we measured total ROS production in M2-like macrophages treated with or without GDNPs. We found that total ROS production in GDNP-treated M2-like macrophages was higher than in untreated macrophages (Fig. 4c). We further investigated whether hydrogen peroxide was induced by GDNPs. As shown in Fig. 4d, treatment with GDNPs resulted in a 14-fold increase in hydrogen peroxide production in M2-like macrophages. Thus, treatment of macrophages with GDNPs increases the production of ROS, which is known to contribute to the tumoricidal function of M1-like macrophages.

### Lipids and proteins of GDNPs alter macrophage polarization

**Fig. 5:**
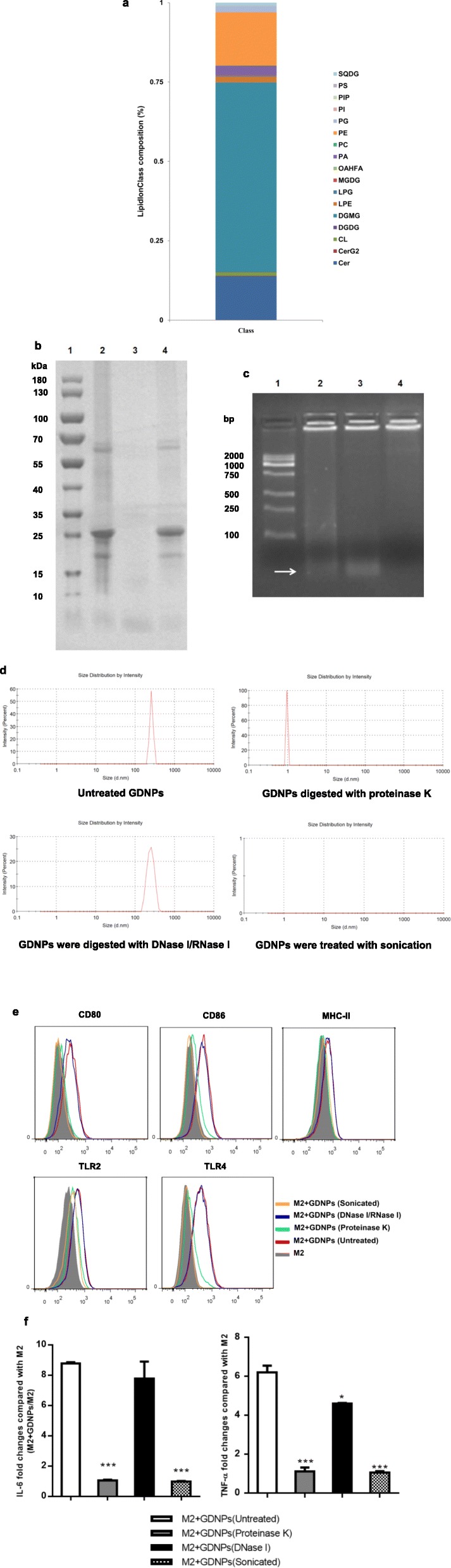
Analysis of the GDNPs components involved in macrophage polarization. **a** The percentages of lipid species in GDNPs. **b** GDNPs were digested with proteinase K. The proteins in GDNPs were analyzed via 12% SDS polyacrylamide gel electrophoresis. (1.Markers, 2.GDNPs, 3.GDNPs digested with proteinase K, 4. GDNPs digested with DNase I/RNase I). **c** GDNPs were digested with DNase I/RNase I. The nucleic acids in GDNPs were analyzed via agarose gel electrophoresis. (1.Markers, 2.GDNPs, 3.GDNPs digested with proteinase K, 4. GDNPs digested with DNase I/RNase I). **d** Particle size of the GDNPs was treated with different reagents. **e** GDNPs (10 μg/ml) subjected to the indicated treatments were co-incubated with M2-like macrophages for 48 h. Macrophages were collected and stained with antibodies against the indicated surface markers. The expression of surface markers on macrophages was analysed by flow cytometry (grey shaded histograms indicate complete medium; red line indicates exposure to untreated GDNPs; green line indicates exposure to GDNPs treated with proteinase K; blue line indicates exposure to GDNPs treated with DNase I and RNase I; orange line indicates exposure to sonicated GDNPs). **f** M2-like macrophages were incubated with GDNPs subjected to the indicated treatments for 48 h; IL-6 and TNF-α in the supernatants were measured by ELISAs. The results represent three independent experiments as the mean ± SEM. **P < 0.01, ***P < 0.001 compared with M2 + GDNPs (untreated); evaluated using Student’s *t* test (f)

To explore which components in GDNPs mediate the polarization of macrophages. We assessed comparative lipid profiles generated from lipidomic analysis (Additional file 1: Table S3). The results revealed that GDNPs were enriched with digalactosyl monoacylglycerol (DGMG, 59.4%), phosphatidyl ethanolamine (PE, 16.8%) and ceramide (Cer, 13.8%) (Fig. 5a). In contrast, the majorities of the lipid in other plants derived nanoparticles were phosphatidylcholine (PC) and glycerophosphate (PA), whereas DGMG and Cer were not detected [34].

Next, we digested GDNPs with proteinase K or DNase I/RNase I or ultrasonic and used these protein- and nucleic acid-depleted GDNPs to treat M2-like macrophages (Fig. 5b-d). Proteinase K treatment of EVs was shown to significantly reduce their uptake by ovarian cancer cells which strongly supports the role of proteins in the EV uptake pathway [35]. Many EV proteins have been shown to interact with membrane receptors on target cells [36, 37]. We found that DNase I/RNase I treatment did not affect GDNP-induced up-regulation of M1-related surface markers (Fig. 5e), suggesting that GDNP-associated nucleic acids were not involved in macrophage polarization. By contrast, up-regulation of these surface markers did not occur in macrophages stimulated with proteinase K-digested GDNPs, indicating that GDNP proteins may participate in the effect of these particles on macrophages polarization.

In addition, no significant differences in IL-6 and TNF-α secretion were observed in macrophages exposed to DNase I/RNase I-digested GDNPs compared to macrophages exposed to undigested GDNPs. However, only small amounts of IL-6 and TNF-α were detected when macrophages were treated with proteinase-digested GDNPs (Fig. 5f). Interestingly, treatment with sonicated GDNPs resulted in both down-regulation of M1-associated surface markers and a significant reduction in IL-6 and TNF-α secretion by M2-like macrophages compared with treatment with unsonicated GDNPs, indicating that the intact structure of GDNPs is necessary for macrophage polarization.

### Uptake of GDNPs by macrophages through phagocytosis

To further evaluate the routes of uptake of GDNP by macrophages, the percentage of DiI-GDNPs in cells was examined by using confocal microscopy (Additional file 2: Figure S6a) and FACS analysis, and determined by quantitative analysis of DiI-GDNPs^+^ cells (Additional file 2: Figure S6b and c). Our results indicated that uptake of GDNPs was remarkably inhibited by LY294002, but treatment with 5-(N,N-Dimethyl) amiloride hydrochloride (EIPA), the macropinocytosis inhibitor, resulted in no reduction of GDNPs uptake, suggesting that macropinocytosis is not main route of macrophages internalizing GDNPs. By FACS analysis, we further demonstrated that macropinocytosis inhibition had no apparent effect on the polarization of macrophages with GDNPs treatment, compared with phagocytosis inhibition (Additional file 2: Figure S6d). These findings suggested that the polarization of macrophages depended on GDNPs internalization.

### GDNPs inhibit mouse melanoma growth in vivo

**Fig. 6:**
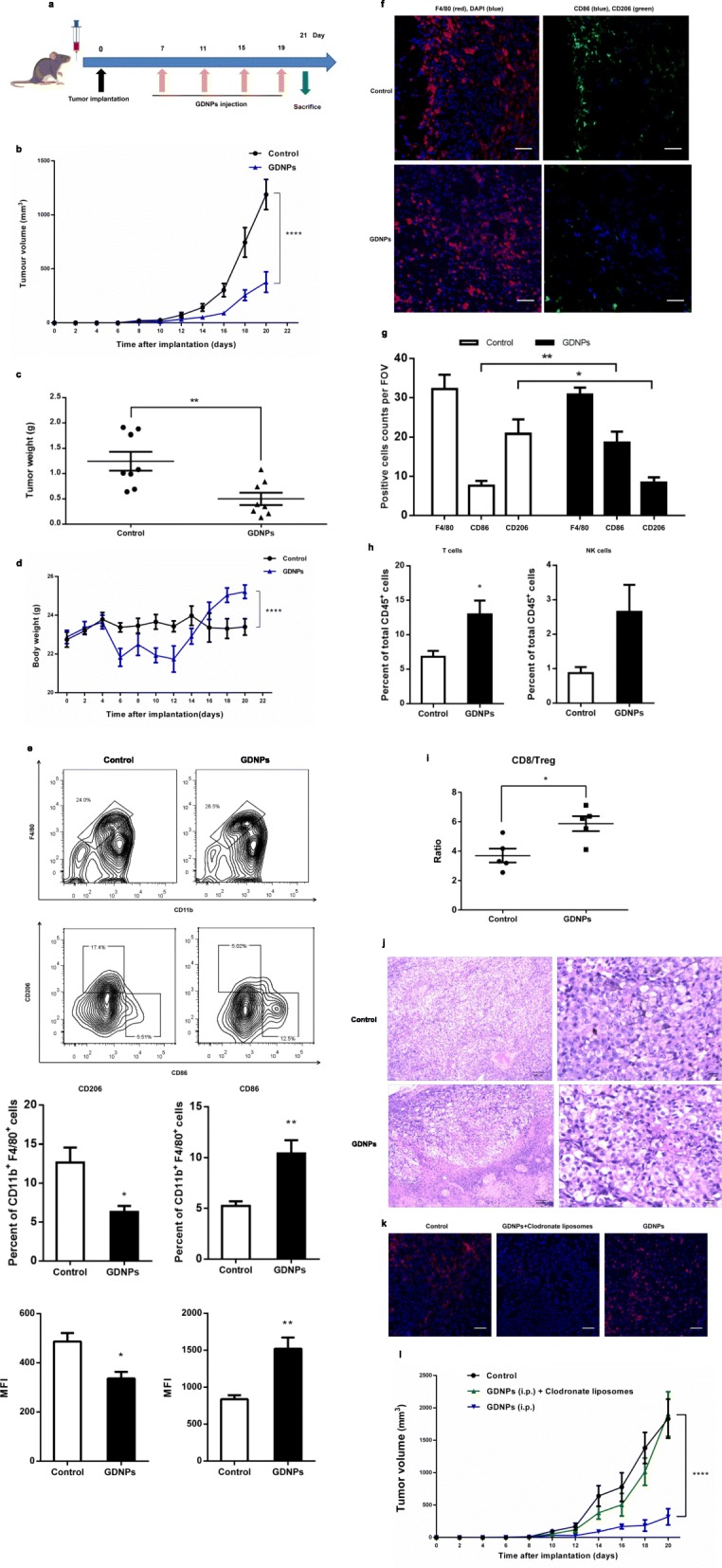
GDNPs inhibit melanoma tumor growth in vivo. **a** Schematic of the B16F10 melanoma model and GDNPs/vehicle treatment regimen. At the end of the experiments, mice were sacrificed, and the anticancer effects in each group were evaluated and compared (*n* = 8). **b** Tumor growth profiles in different treatment groups (*n* = 8). **c** Tumor weights at the end of the experiment were compared (*n* = 8). **d** Body weight changes in different treatment groups. Mouse body weight was normalized to that at the time of implantation (*n* = 8). **e** Flow cytometry analysis and quantification of M1 (CD86^hi^) and M2 (CD206^hi^) cell populations in TAMs at day 21 post-implantation (*n* = 5). Representative flow cytometry analysis and quantification of CD11b^+^ F4/80^+^ (TAM) cell populations in tumors at day 21 post-implantation; expression of CD206^hi^ (M2) and CD86^hi^ (M1) in CD11b^+^ F4/80^+^ cell populations. The histogram bars show the percentage and mean fluorescence intensity (MFI) change in each cell population (M2 and M1) in GDNP-treated groups compared to the PBS-treated controls. **f** Representative immunofluorescence staining for F4/80 (red), CD206 (green) and CD86 (blue) of B16F10 tumor sections obtained at 21 days post-implantation (Scale bar = 100 μm). **g** The number of positive cells in 10 randomly selected fields of view (FOV) were counted and quantified for 3 tumors per group. **h** The percentages of T cells and NK cells in total CD45^+^ TILs. **i** Quantification by flow cytometry of ratio of CD8^+^/Treg in CD45^+^ TILs in B16F10 tumors at day 14 with GDNPs treatment. **j** Tumor tissues were excised, fixed and sectioned. H&E staining of tumor tissues from each group was used to evaluate the death of tumor cells (Scale bar = 100/20 μm). **k** B16F10-bearing C57BL/6 mice were treated with GDNPs in the presence or absence of clodronate liposomes, which deplete macrophages (*n* = 5). Mice in the control group were treated with liposomes containing PBS. Immunofluorescence staining for F4/80 (red) and DAPI (blue) of tumor sections obtained 21 days post-implantation of cancer cells with and without GDNPs and/or clodronate liposome treatment (Scale bar = 100 μm). **l** Serial tumor volume measurements up to day 20 after tumor implantation in mice treated with GDNPs in the presence or absence of clodronate liposomes (*n* = 5). All results represent the mean ± SEM. Two-way ANOVA (b, d, l) and Student’s *t* test (c, e, g, h, i) were used to compare results of different experimental groups for statistically significant difference (*P < 0.05, **P < 0.01, ****P < 0.0001)

Our in vitro results showed that GDNP treatment of macrophages results in altering M2-like polarization, increased production of proinflammatory cytokines and ROS, and induction of apoptosis of melanoma cells. Next we investigated whether treatment with GDNPs had a similar effect in vivo and resulted in a change in TAM polarization towards a favourable antitumor profile. To this end, we established a tumor-bearing mouse model by subcutaneously inoculating B16F10 cancer cells into the right flanks of male C57BL/6 mice as described previously [21]. After 7 days, mice were treated with PBS (control) or GDNPs every four days and the experiment was ended on day 21 post-tumor implantation (Fig. 6a). Treatment with GDNPs significantly suppressed tumor growth as measured by tumor volume from day 14 of treatment (Fig. 6b). In addition, at day the end of the experiment, tumor weight in GDNP-treated mice was reduced by 53% (Fig. 6c; Additional file 2: Figure S7a), and these mice had gained more body weight than control mice (Fig. 6d).

To better understand the antitumor mechanism induced by GDNPs, we purified the tumor-infiltrating leukocytes (TILs) using specific anti-CD45 antibodies and analysed the different cell populations by multicolour flow cytometry and immunofluorescence (IF). Treatment of B16F10-allografted mice with GDNPs for 21 days resulted in a significantly higher quantity of M1 macrophages in the TIL population (Fig. 6e) than in the PBS treated control mice. The ratio of M1 cell counts (CD86^+^/total macrophages) was significantly higher in GDNP-treated mice than in control mice. In addition, the median fluorescence intensity (MFI) of CD86, a marker of M1-like macrophages, was significantly elevated in GDNP-treated mice. Meanwhile, the proportion of CD206-positive cells, which indicate M2-like macrophages, decreased among all examined macrophages in GDNP-treated mice. These results were further confirmed by IF staining (Fig. 6f and g). We evaluated major immune cell in TME by FACS analyses at day 21 after B16F10 cancer cells implantation. The proportion of T cells and natural killer (NK) cells in the TILs population were increased in the tumors of GDNP-treated mice (Fig. 6h). And the GDNPs treatment correlates with increased T cell infiltrates and a higher CD8^+^/regulatory T cell ratio at day 21 in tumors (Fig. 6i). In comparison, there are no remarkable differences in the quantity of DCs, B cells and granulocytes in the TME (Additional file 2: Figure S7b). FACS and IF images analysis was also done on main sets of TILs from tumor-bearing mice received i.p. injection of DiI-labelled GDNPs. The results indicated that 14 days after GDNPs were i.p. administration, the majority of them taken up by macrophages (23.2%) in the TME, with minority populations of DCs (3.92%), granulocytes (3.42%) and CD8^+^ T cells (2.51%) (Additional file 2: Figure S7c and d). Confocal fluorescence analysis further indicated GDNPs co-localized mainly with macrophages signal in liver, spleen and tumor (Additional file 2: Figure S7e). Moreover, the impact of GDNPs was compromised in mice lacking T cells (after anti-CD8^+^ T cell depletion) (Additional file 2: Figure S8). Therefore, our results showed that GDNP-treatment in vivo alters M2-like polarization to M1-like polarization with the consequent increase in T cells in the TME.

Finally, we studied whether treatment with GDNPs induces tumor cell apoptosis in vivo by histopathology analysis. Similarly to what we had observed in vitro, we found that treatment with GDNPs induced death of tumor cells in TME (Fig. 6j). Overall these results indicate that GDNP treatment polarizes M1 macrophages, represses M2 macrophages and subsequently induces tumor cell death, thus inhibiting tumor growth.

To further determine whether macrophage was responsible for the GDNP-mediated tumor growth inhibition, we compared tumor growth and M1 polarization in tumor-bearing mice treated with either GDNPs or GDNPs plus clodronate liposomes, which deplete TAMs. Control mice inoculated with melanoma cells were treated with control liposomes that were coated in PBS. As expected, macrophages were depleted efficiently with clodronate liposomes treatment (Fig. 6k). We found that no significant tumor growth inhibition in mice treated with GDNPs plus clodronate liposomes (Fig. 6l). Control mice also showed no significant tumor growth inhibition. These results confirmed that GDNPs inhibit tumor growth through a TAM-dependent mechanism.

## Discussion

EVs from mammalian cells have been recognized as one of the major mechanisms of intercellular communication. EVs can induce signalling via receptor-ligand interactions, be internalized by endocytosis and/or phagocytosis, or even fuse with the target cell’s membrane to deliver their content into its cytosol, thereby modifying the physiological state of the recipient cell [38]. Compared to synthesized nanoparticles, EVs from mammalian cells offer multiple benefits, such as low toxicity and good tissue-specific targeting [39, 40]. However, potential biohazard risks to the recipient and large-scale economical production are challenging issues in the therapeutic application of mammalian-derived EVs [41].

Several groups have independently demonstrated that nanoparticle-like EVs are also produced by several types of plants, and play different roles in plant cell-cell communication [42]. These plant-derived EVs have no known potential toxicity to humans, and can be produced in large quantities. Thus, the use of plant-derived EVs has been demonstrated as a vector to deliver chemotherapeutic agents, microRNAs, DNA and proteins for cancer treatment and intestinal bowel disease [43, 44]. These observations prompted us to explore whether similar nano-sized EVs are produced by certain natural herbs, which may have medicinal properties and could easily be taken up by mammalian cells to mediate cross-species communication.

In particular, we focused on *P. ginseng*, which is well known for its multiple pharmacological properties, including anticancer, anti-obesity and neuroprotective activities and is used as a medicinal herb or a dietary supplement worldwide. The extracts of ginseng, such as, ginsenoside (unique triterpenoid saponins), phenols and acidic polysaccharides have been known to exhibit numerous pharmacological efficacies. However, the clinical application of Ginseng phytochemicals is significantly hampered due to its limited solubility, low oral bioavailability and nontargeted cytotoxicity to normal cells. Most reported nanoparticles of ginsenoside by various nanocarriers, such as polymer-drug conjugates, liposomes and metal nanoparticles could be a promising candidate against cancer and various other diseases [45]. In this study, we found that nanoparticle-like EVs are extracted from *P. ginseng* root. These GDNPs can be isolated by ultracentrifugation followed by density gradient centrifugation. Our electron microscopy and zeta potential analyses indicate that these GDNPs have a structure similar to those of mammalian-derived EVs, and contain cytosolic components such as proteins, lipids and nucleic acids. Moreover, GDNPs accumulation was dominated by macrophages, relative to other immune cell types and the uptake of GDNPs by macrophages was phagocytosis dependent.

In solid tumors, such as melanoma and breast cancer, infiltrating TAMs are abundant and linked with poor clinical outcome [46]. Thus, targeting TAMs is thought to be a promising strategy for cancer therapy. Several small molecule-based or antibody-based drugs have been developed to deplete TAM populations [47, 48]. These approaches have been demonstrated to delay tumor progression in animal models and are currently being evaluated in the clinic [49]. However, experimental studies have also shown that depletion of TAMs may not suffice to trigger durable anticancer effects [50, 51]. Because plasticity and flexibility are key features of macrophages, an alternative therapeutic approach consists of altering/reprogramming TAMs [52]. These reprogramming strategies offer the possibility of not only abolishing phagocytes’ tumor-supportive functions but also actively promoting their antitumor immune actions [53, 54]. As nanoparticles are often easily internalized by macrophages, TLR7/8 agonist-loaded nanoparticles were delivered to TAMs efficiently altering the polarization of TAMs, which promoted antitumor immune responses [55]. Despite these observations, how to drive reprogramming with high potency is a challenge.

Our data demonstrated that GDNPs significantly suppressed IL-4 and IL-13-induced M2-like polarization of macrophages, as illustrated by the reduced expression of M2 surface markers (CD206) and down-regulation of M2-related surface markers. Considering that in most cases, the patterns of gene expression of macrophages in response to various stimuli are heterogeneous and do not precisely fit the published patterns associated with these M1/M2 designations, we further analysed the secretion of related cytokines in M2-like macrophages in response to GDNPs. The results showed that treatment with GDNPs substantially increased the secretion of M1-associated cytokines, such as TNF-α, IL-12 and IL-6. We speculated that these proinflammatory cytokines produced from GDNPs-activated macrophages favored Th1 immune response to promote CD8^+^ T cells activation. Moreover, we analysed the antitumor function of GDNP-treated macrophages. Continued M1 polarization maintained the production of cytokines and cytotoxic hydroxyl radicals, thus inducing apoptosis of cancer cells.

Recent studies have shown that mouse macrophages express TLR1–9, TLR11 and TLR13. TLRs play a central role in macrophage activation, differentiation, and polarization. Most TLR signals require the adaptor protein MyD88, activating the classical NF-κB cascade. Here we found that M1-related surface markers and secretion of associated cytokines were not induced in response to GDNP treatment in macrophages derived from MyD88- or TLR4-deficient mice, while TLR2 deficiency barely affected the macrophage response to GDNP treatment. Our data provide new evidence that GDNPs trigger macrophage polarization in a TLR4/MyD88-dependent manner.

Ceramide was identified as a TLR4 agonist and has been demonstrated to be a powerful tumor suppressor. However, it has not been used as a chemotherapeutic agent because of its cell impermeability and precipitation in aqueous solution [56, 57]. Recently, Li et al have reported that vein injection of nanoliposome C6-ceramide, a cell membrane permeable form of ceramide, slows growth of liver tumors in mice, through suppressing tumor-associated macrophage functions and enhancing tumor antigen-specific CD8^+^ T-cell activity [58]. As the authors have shown, the supplementation of exogenous nanoliposome ceramide may prime and alter systematic innate immune cell activities, particularly after immunization using tumor antigens. Thus, we hypothesized that ceramide lipids of GDNPs may play an important role in macrophage polarization via TLR4 activation. The impairment observed for GDNPs treated with proteinase K indicated that proteins could also be required for their efficacy. In addition, the size and structure of nanoparticulate enhanced bioavailability and retention of GDNPs in vivo, facilitating its potential clinical use as a drug.

Importantly, we found that GDNP treatment significantly reduced tumor growth in mice inoculated with melanoma cells. This effect was mediated by macrophages and involved M2-like macrophages switching from an immune-supportive M2-like (CD11b^+^F4/80^+^CD206^+^) phenotype to a more tumoricidal M1-like (CD11b^+^F4/80^+^CD86^+^) state in the TME. At the same time, the percentages of T cells increased markedly. We speculate that increased M1 macrophages induced the activation of the immune cells in the TME. These results further validate the TAM-dependent mechanism of tumor growth inhibition by GDNPs.

## Conclusions

**Fig. 7:**
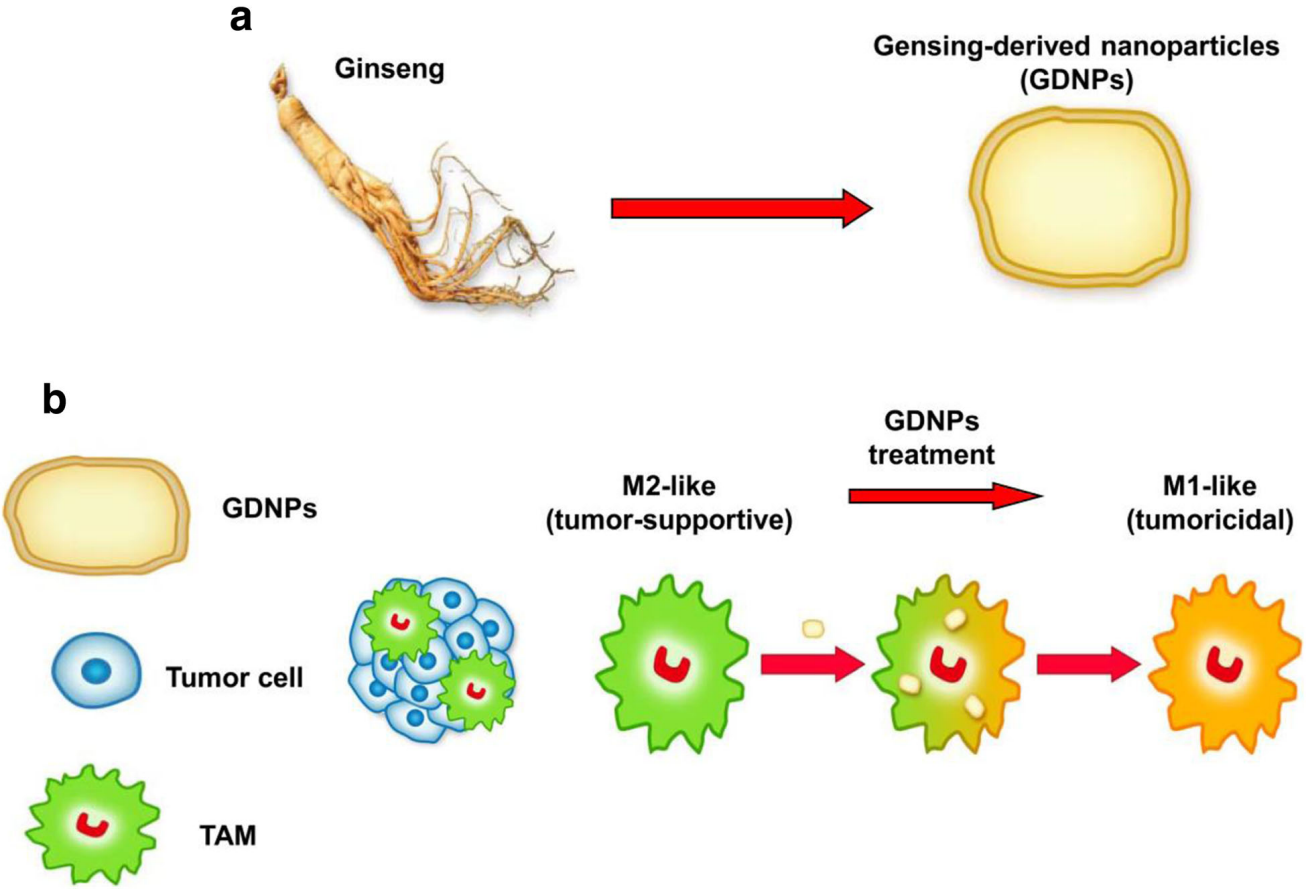
Proposed strategy for the preparation of ginseng-derived nanoparticles (GDNPs) to reprogramme macrophage polarization. **a** GDNPs were isolated and purified from extracts of ginseng roots by multiple ultracentrifugation. **b** The schematic diagram showed that GDNPs targeted tumor-associated macrophages (TAMs), which are canonically M2-like (tumor-supportive) in the tumor microenvironment

In this study, we demonstrated for the first time that GDNPs can alter M2-like polarization both in vitro and in vivo, which contributes to an antitumor response (Fig. 7a and b). The polarization of macrophages induced by GDNPs is largely dependent on TLR4 and MyD88 signalling. More importantly, GDNPs can be easily produced in a scalable manner. Our results suggest that GDNPs as an immunomodulator participate in mammalian immune response and may represent a new class of nano-drugs in cancer immunotherapy.

## Supplementary information

**Additional file 1: Table S1.** Antibodies used for flow cytometry and immunofluorescence. **Table S2.** Primer sequences for real-time RT-PCR analysis. **Table S3.** Lipid profile of nanoparticles purified from GDNPs.

**Additional file 2: Figure S1.** Analysis of composition of GDNPs. **Figure S2.** Biodistribution and stability of GDNPs *In vivo*. **Figure S3.** C57BL/6 mice were treated with i.p. injection of DiR-lablled GDNPs in the presence or absence of clodronate liposomes. **Figure S4.** Biocompatibility of GDNPs *in vitro* and *in vivo*. **Figure S5.** FACS analysis of related surface markers of macrophages treated with nanoparticles from different plants. **Figure S6.** Uptake of GDNPs by macrophages depends on phagocytosis. **Figure S7.** GDNPs inhibit melanoma tumor growth in vivo. **Figure S8.** Mean tumor volume of subcutaneous B16F10 tumors in GDNPs versus PBS-treated mice with or without CD8^+^ T cell depletion by anti-CD8 antibody. **Figure S9.** Flow cytometry gating strategy for polarization analysis of tumor associated macrophages (TAMs).

## Data Availability

The datasets used and/or analyzed during the current study are available from the corresponding author on reasonable request.

## Abbreviations

7-AAD: 7-Aminoactinomycin D
BMDMs: Bone marrow derived macrophages
CCL: Chemokine ligand
CD: Cluster of differentiation
CDNP: Cucumber -derived nanoparticle
Cer: Ceramide
CL: Clodronate liposomes
CM: Conditioned medium
DAPI: 4′,6-Diamidino-2-Phenylindole
DC: Dendritic cell
DGMG: Digalactosyl monoacylglycerol
DiI & DiR: Dialkylcarbocyanine Probes
DMEM: Dulbecco’s Modified Eagle’s Medium
EIPA: 5-(N,N-Dimethyl) amiloride hydrochloride
ELISA: Enzyme-linked immunosorbent assay
EVs: Extracellular vesicles
FACS: Fluorescence activated Cell Sorting
FBS: Foetal bovine serum
GAPDH: Glyceraldehyde-3-phosphate dehydrogenase
GDNP: Ginseng-derived nanoparticle
GO: Gene Ontology
HBSS: Hank’s balanced salt solution
HE: Hematoxylin-eosin
IF: Immunofluorescence
IL: Interleukin
KDNP: kiwi fruit-derived nanoparticle
M-CSF: Macrophage colony-stimulating factor
MFI: Median fluorescence intensity
MCP: Monocyte chemoattractant protein
MPS: Mononuclear phagocyte system
MS: Mass spectrometry
MyD88: Myeloid differentiation antigen 88
OCT: Optimal cutting temperature
PA: glycerophosphate
PAMP: Pathogen-associated molecular pattern
PBMC: Peripheral blood mononuclear cell
PBS: Phosphate buffer saline
PC: Phosphatidylcholine
PE: Phosphatidyl ethanolamine
ROS: Reactive oxygen species
RT-PCR: Real-time polymerase chain reaction
SDS: Sodium dodecyl sulfate
TAMs: Tumor-associated macrophages
TEM: Transmission electron microscopy
TIL: Tumor-infiltrating leukocyte
TLR: Toll-like receptor
TME: Tumor microenvironment
TNF: Tumor necrosis factor
WT: Wild-type
