# Supplementary material for: Ginseng-derived nanoparticles alter macrophage polarization to inhibit melanoma growth
Source: J Immunother Cancer. 2019 Nov 27;7:326. doi: 10.1186/s40425-019-0817-4 (PMC6882204; doi:10.1186/s40425-019-0817-4)
Supplement: Supplementary file 1 — Additional file 1: Table S1. Antibodies used for flow cytometry and immunofluorescence. Table S2. Primer sequences for real-time RT-PCR analysis. Table S3. Lipid profile of nanoparticles purified from GDNPs. [file 40425_2019_817_MOESM1_ESM.docx]

**Additional file 1**

**Additional file 1: Table S1**

**Table S1. Antibodies used for flow cytometry and immunofluorescence**

| **Antigen** | **Clone** | | **Fluorophore** | | **Company** | **Catalog No.** | |
| --- | --- | --- | --- | --- | --- | --- | --- |
| Mouse CD11b | M1/70 | APC | | Thermo(eBioscience) | | | 17-0112 |
| Mouse F4/80 | BM8 | PE | | Thermo(eBioscience) | | | 12-4801 |
| Mouse CD3 | 17A2 | FITC | | Thermo(eBioscience) | | | 11-0032 |
| Mouse CD11c | N418 | FITC | | Thermo(eBioscience) | | | 11-0114 |
| Mouse F4/80 | BM8 | PE/Cy7 | | BioLegend | | | 123112 |
| Mouse F4/80 | BM8 | FITC | | BioLegend | | | 123108 |
| Mouse CD206 | C068C2 | APC | | BioLegend | | | 141708 |
| Mouse CD80 | 16-10A1 | APC | | BioLegend | | | 104713 |
| Mouse CD86 | PO3 | PE | | BioLegend | | | 105106 |
| Mouse I-A^b^ | AF6-120.1 | FITC | | BioLegend | | | 116406 |
| Mouse TLR2 | T2.5 | FITC | | BioLegend | | | 121805 |
| Mouse TLR4 | MTS510 | PE/Cy7 | | BioLegend | | | 117609 |
| Mouse CD45 | 30-F11 | APC | | BioLegend | | | 103112 |
| Mouse CD11b | M1/70 | FITC | | BioLegend | | | 101205 |
| Mouse CD4 | GK1.5 | PE/Cy7 | | BioLegend | | | 100421 |
| Mouse CD45 | 30-F11 | Brilliant Violet 510 | | BioLegend | | | 103137 |
| Mouse CD45R | RA3-6B2 | Brilliant Violet 510 | | BioLegend | | | 103247 |
| Mouse CD11b | M1/70 | APC/Cy7 | | BioLegend | | | 101225 |
| Mouse CD335 | 29A1.4 | PerCP/Cy5.5 | | BioLegend | | | 137609 |
| Mouse Ly-6G/6C | RB6-8C5 | APC | | BioLegend | | | 108411 |
| Mouse CD86 | GL-1 | APC/Cy7 | | BioLegend | | | 105029 |
| Mouse CD206 | C068C2 | PE | | BioLegend | | | 141706 |
| Mouse CD3 | 145-2C11 | APC/Cy7 | | BioLegend | | | 100330 |
| Mouse NK1.1 | PK136 | PE | | BioLegend | | | 108707 |
| Mouse CD86 | GL-1 | Alexa Fluor® 647 | | BioLegend | | | 105020 |
| Mouse CD206 | C068C2 | Alexa Fluor® 488 | | BioLegend | | | 141710 |
| Mouse CD335 | 29A1.4 | PerCP/Cy5.5 | | BioLegend | | | 137609 |
| Mouse CD3 | 17A2 | Brilliant Violet 421 | | BioLegend | | | 100228 |
| Mouse CD45 | 30-F11 | Brilliant Violet 421 | | BioLegend | | | 103134 |
| Mouse F4/80 | BM8 | Brilliant Violet 421 | | BioLegend | | | 123137 |
| Mouse Ly6G | 1A8 | Alexa Fluor® 647 | | BioLegend | | | 127610 |
| Mouse CD8 | 53-6.7 | FITC | | BioLegend | | | 100706 |
| Mouse CD8 | 53-6.7 | PE | | BioLegend | | | 100707 |
| Mouse F4/80 | BM8 | Alexa Fluor® 594 | | BioLegend | | | 123140 |
| Mouse F4/80 | BM8 | Alexa Fluor® 647 | | BioLegend | | | 123121 |
| Mouse CD25 | PC61.5 | APC | | Thermo(eBioscience) | | | 17-0251-82 |
| Mouse FoxP3 | FJK-16s | PE | | Thermo(eBioscience) | | | 12-5773-82 |
|  |  |  | |  | | |  |

**Additional file 1: Table S2**

**Table S2.** Primer sequences for real-time RT-PCR analysis

| **Gene** | **Forward** | **Reverse** |
| --- | --- | --- |
| GAPDH | TGAAGCAGGCATCTGAGGG | CGAAGGTGGAAGAGTGGGAG |
| iNOS | CAGAGGACCCAGAGACAAGC | TGCTGAAACATTTCCTGTGC |
| TNF-α | CCTCCCTCTCATCAGTTCTA | ACTTGGTGGTTTGCTACGAC |
| IL-6 | CCCCAATTTCCAATGCTCTCC | TTGGTCCTTAGCCACTCCTTC |
| CCL 2 | CTTCTGGGCCTGCTGTTCA | CCAGCCTACTCATTGGGATCA |
| CCL 3 | CATATGGAGCTGACACCCCG | GAGCAAAGGCTGCTGGTTTC |
| CD 86 | TCAATGGGACTGCATATCTGCC | GCCAAAATACTACCAGCTCACT |
| Arg-1 | CAGAAGAATGGAAGAGTCAG | CAGATATGCAGGGAGTC |
| CD206 | CAGGTGTGGGCTCAGGTAGT | TGTGGTGAGCTGAAAGGTGA |
| IL-10 | CTTACTGACTGGCATGAGGATCA | GCAGCTCTAGGAGCATGTGG |
| CHI 313 | GGGCATACCTTTATCCTGAG | CCACTGAAGTCATCCATGTC |

**Additional file 1: Table S3**

**Table S3. Lipid profile of nanoparticles purified from GDNPs**

| **Mass** | **Compound** | **Compound** | **% of total Lipid** |
| --- | --- | --- | --- |

| 1141.730 | CL(49:0) | | C58H111O17P2 | | 0.007 | |  |
| --- | --- | --- | --- | --- | --- | --- | --- |
| 1349.949 | CL(16:0/16:0/16:0/16:1) | | C73H139O17P2 | | 0.006 | |  |
| 1347.934 | CL(16:0/16:1/16:0/16:1) | | C73H137O17P2 | | 0.119 | |  |
| 1345.918 | CL(16:0/16:1/16:1/16:1) | | C73H135O17P2 | | 0.009 | |  |
| 1361.949 | CL(17:1/16:0/16:1/16:0) | | C74H139O17P2 | | 0.022 | |  |
| 1377.981 | CL(16:0/16:0/16:1/18:0) | | C75H143O17P2 | | 0.037 | |  |
| 1375.965 | CL(18:1/16:0/16:0/16:1) | | C75H141O17P2 | | 0.023 | |  |
| 1375.965 | CL(18:2/16:0/16:0/16:0) | | C75H141O17P2 | | 0.011 | |  |
| 1373.949 | CL(18:1/16:1/16:0/16:1) | | C75H139O17P2 | | 0.107 | |  |
| 1369.918 | CL(18:4/16:0/16:1/16:0) | | C75H135O17P2 | | 0.033 | |  |
| 1387.965 | CL(17:1/16:0/18:1/16:1) | | C76H141O17P2 | | 0.018 | |  |
| 1383.934 | CL(67:5) | | C76H137O17P2 | | 0.006 | |  |
| 1403.996 | CL(18:1/16:0/16:1/18:0) | | C77H145O17P2 | | 0.026 | |  |
| 1401.981 | CL(18:1/16:1/18:1/16:0) | | C77H143O17P2 | | 0.014 | |  |
| 1399.965 | CL(16:0/16:0/16:1/20:3) | | C77H141O17P2 | | 0.022 | |  |
| 1399.965 | CL(18:1/16:1/16:1/18:1) | | C77H141O17P2 | | 0.030 | |  |
| 1399.965 | CL(18:2/16:0/16:1/18:1) | | C77H141O17P2 | | 0.010 | |  |
| 1397.949 | CL(20:4/16:0/16:0/16:1) | | C77H139O17P2 | | 0.007 | |  |
| 1395.934 | CL(18:4/16:0/16:1/18:1) | | C77H137O17P2 | | 0.033 | |  |
| 1425.981 | CL(18:1/16:0/16:1/20:3) | | C79H143O17P2 | | 0.014 | |  |
| 1447.965 | CL(18:2/18:2/18:2/18:2) | | C81H141O17P2 | | 0.024 | |  |
| **Total CL** |  | |  | | **0.577** | |  |
| 342.265 | Cer(d17:1+hO/2:0) | | C19H36O4N1 | | 0.080 | |  |
| 538.4841 | Cer(d17:1+hO/16:0) | | C33H64O4N1 | | 0.007 | |  |
| 584.4896 | Cer(d17:1+hO/16:0) | | C34H66O6N1 | | 0.078 | |  |
| 554.479 | Cer(d16:1+hO/17:0+O) | | C33H64O5N1 | | 0.026 | |  |
| 600.5209 | Cer(d18:0+pO/16:0) | | C35H70O6N1 | | 0.147 | |  |
| 552.4997 | Cer(d34:1+O) | | C34H66O4N1 | | 0.006 | |  |
| 552.4997 | Cer(d18:1+hO/16:0) | | C34H66O4N1 | | 0.013 | |  |
| 598.5052 | Cer(d18:1+hO/16:0) | | C35H68O6N1 | | 2.324 | |  |
| 568.4946 | Cer(d34:1+hO+O) | | C34H66O5N1 | | 0.332 | |  |
| 580.4946 | Cer(d18:2/16:0) | | C35H66O5N1 | | 0.190 | |  |
| 596.4896 | Cer(d18:1+hO/16:1) | | C35H66O6N1 | | 0.045 | |  |
| 596.4896 | Cer(d18:1+hO/16:1) | | C35H66O6N1 | | 0.027 | |  |
| 612.5209 | Cer(d17:1+hO/18:0) | | C36H70O6N1 | | 0.011 | |  |
| 610.5052 | Cer(d17:1+hO/18:1) | | C36H68O6N1 | | 0.015 | |  |
| 608.4896 | Cer(d17:1+hO/18:2) | | C36H66O6N1 | | 0.010 | |  |
| 628.5522 | Cer(d17:0+pO/19:0) | | C37H74O6N1 | | 0.009 | |  |
| 580.531 | Cer(d18:1+hO/18:0) | | C36H70O4N1 | | 0.001 | |  |
| 626.5365 | Cer(d18:1+hO/18:0) | | C37H72O6N1 | | 0.165 | |  |
| 596.5259 | Cer(d17:1+hO/19:0+O) | | C36H70O5N1 | | 0.023 | |  |
| 626.5365 | Cer(d18:0+pO/18:1) | | C37H72O6N1 | | 0.020 | |  |
| 624.5209 | Cer(d18:1+hO/18:1) | | C37H70O6N1 | | 0.086 | |  |
| 578.5154 | Cer(d18:1+hO/18:1) | | C36H68O4N1 | | 0.024 | |  |
| 606.5103 | Cer(d16:0/20:3) | | C37H68O5N1 | | 0.253 | |  |
| 622.5052 | Cer(d36:3+O) | | C37H68O6N1 | | 0.012 | |  |
| 576.4997 | Cer(d18:1+hO/18:2) | | C36H66O4N1 | | 0.014 | |  |
| 622.5052 | Cer(d18:1+hO/18:2) | | C37H68O6N1 | | 0.145 | |  |
| 592.4946 | Cer(d36:3+hO+O) | | C36H66O5N1 | | 0.003 | |  |
| 638.5001 | Cer(d36:3+hO+O) | | C37H68O7N1 | | 0.058 | |  |
| 604.4946 | Cer(d18:2/18:2) | | C37H66O5N1 | | 0.034 | |  |
| 636.4845 | Cer(d36:4+2O) | | C37H66O7N1 | | 0.021 | |  |
| 574.4841 | Cer(d18:1+hO/18:3) | | C36H64O4N1 | | 0.006 | |  |
| 620.4896 | Cer(d18:1+hO/18:3) | | C37H66O6N1 | | 0.012 | |  |
| 618.4739 | Cer(d18:1+hO/18:4) | | C37H64O6N1 | | 0.013 | |  |
| 640.5522 | Cer(d17:1+hO/20:0) | | C38H74O6N1 | | 0.006 | |  |
| 638.5365 | Cer(d17:1+hO/20:1) | | C38H72O6N1 | | 0.007 | |  |
| 654.5678 | Cer(d18:1+hO/20:0) | | C39H76O6N1 | | 0.024 | |  |
| 670.5627 | Cer(d18:0+pO/20:1+O) | | C39H76O7N1 | | 0.068 | |  |
| 650.5365 | Cer(d17:1+hO/21:2) | | C39H72O6N1 | | 0.025 | |  |
| 668.5835 | Cer(d17:1+hO/22:0) | | C40H78O6N1 | | 0.024 | |  |
| 638.5729 | Cer(d17:1+hO/22:0+O) | | C39H76O5N1 | | 0.027 | |  |
| 684.6148 | Cer(d17:0+pO/23:0) | | C41H82O6N1 | | 0.016 | |  |
| 654.6042 | Cer(d17:0+pO/23:0+O) | | C40H80O5N1 | | 0.015 | |  |
| 636.5936 | Cer(d18:1+hO/22:0) | | C40H78O4N1 | | 0.006 | |  |
| 682.5991 | Cer(d18:1+hO/22:0) | | C41H80O6N1 | | 0.156 | |  |
| 652.5885 | Cer(d17:1+hO/23:0+O) | | C40H78O5N1 | | 0.144 | |  |
| 680.5835 | Cer(d17:1+hO/23:1) | | C41H78O6N1 | | 0.013 | |  |
| 650.6093 | Cer(d18:1+hO/23:0) | | C41H80O4N1 | | 0.006 | |  |
| 696.6148 | Cer(d18:1+hO/23:0) | | C42H82O6N1 | | 0.210 | |  |
| 666.6042 | Cer(d17:1+hO/24:0+O) | | C41H80O5N1 | | 0.313 | |  |
| 712.6461 | Cer(d17:0+pO/25:0) | | C43H86O6N1 | | 0.039 | |  |
| 682.6355 | Cer(d17:0+pO/25:0+O) | | C42H84O5N1 | | 0.047 | |  |
| 664.6249 | Cer(d18:1+hO/24:0) | | C42H82O4N1 | | 0.002 | |  |
| 710.6304 | Cer(d18:1+hO/24:0) | | C43H84O6N1 | | 0.036 | |  |
| 680.6198 | Cer(d17:1+hO/25:0+O) | | C42H82O5N1 | | 0.209 | |  |
| 708.6148 | Cer(d17:1+hO/25:1) | | C43H82O6N1 | | 0.067 | |  |
| 726.6617 | Cer(d17:0+pO/26:0) | | C44H88O6N1 | | 0.010 | |  |
| 696.6511 | Cer(d17:0+pO/26:0+O) | | C43H86O5N1 | | 0.016 | |  |
| 678.6406 | Cer(d18:1+hO/25:0) | | C43H84O4N1 | | 0.009 | |  |
| 724.6461 | Cer(d18:1+hO/25:0) | | C44H86O6N1 | | 0.270 | |  |
| 694.6355 | Cer(d17:1+hO/26:0+O) | | C43H84O5N1 | | 0.313 | |  |
| 722.6304 | Cer(d18:1+hO/25:1) | | C44H84O6N1 | | 0.012 | |  |
| 738.6617 | Cer(d17:1+hO/27:0) | | C45H88O6N1 | | 0.068 | |  |
| 708.6511 | Cer(d17:1+hO/27:0+O) | | C44H86O5N1 | | 0.127 | |  |
| 736.6461 | Cer(d17:1+hO/27:1) | | C45H86O6N1 | | 0.009 | |  |
| 752.6774 | Cer(d18:1+hO/27:0) | | C46H90O6N1 | | 0.006 | |  |
| **Total Cer** |  | |  | | | **6.508** | |
| 816.5479 | CerG2(d31:2) | | C43H78O13N1 | | 0.010 | |  |
| 1000.731 | CerG2(d43:2+pO) | | C55H102O14N1 | | 0.046 | |  |
| **Total CerG2** | |  | |  | | **0.056** | |
| 1037.715 | DGDG(43:4) | | C58H101O15 | | 0.019 | |  |
| 1107.72 | DGDG(45:6) | | C61H103O17 | | 0.006 | |  |
| 1061.715 | DGDG(45:6) | | C60H101O15 | | 0.028 | |  |
| 1059.699 | DGDG(45:7) | | C60H99O15 | | 0.004 | |  |
| **Total DGDG** | |  | |  | | **0.057** | |
| 845.4904 | DGMG(27:4) | | C43H73O16 | | 41.505 | |  |
| 845.4904 | DGMG(27:4) | | C43H73O16 | | 0.004 | |  |
| 845.4904 | DGMG(27:4) | | C43H73O16 | | 0.001 | |  |
| 845.4904 | DGMG(27:4) | | C43H73O16 | | 0.000 | |  |
| 845.4904 | DGMG(27:4) | | C43H73O16 | | 0.009 | |  |
| **Total DGMG** | |  | |  | | **41.519** | |
| 452.2783 | LPE(16:0) | | C21H43O7N1P1 | | 0.016 | |  |
| 450.2626 | LPE(16:1) | | C21H41O7N1P1 | | 0.684 | |  |
| 464.2783 | LPE(17:1) | | C22H43O7N1P1 | | 0.215 | |  |
| 478.2939 | LPE(18:1) | | C23H45O7N1P1 | | 0.110 | |  |
| 476.2783 | LPE(18:2) | | C23H43O7N1P1 | | 0.024 | |  |
| 550.3878 | LPE(23:0) | | C28H57O7N1P1 | | 0.003 | |  |
| **Total LPE** |  | |  | | **1.051** | |  |
| 483.2728 | LPG(16:0) | | C22H44O9N0P1 | | 0.036 | |  |
| 481.2572 | LPG(16:1) | | C22H42O9N0P1 | | 0.065 | |  |
| 509.2885 | LPG(18:1) | | C24H46O9N0P1 | | 0.009 | |  |
| **Total LPG** |  | |  | | **0.110** | |  |
| 753.4795 | MGDG(31:4) | | C41H69O12 | | 0.007 | |  |
| 799.5577 | MGDG(16:0/18:2) | | C44H79O12 | | 0.021 | |  |
| 825.5734 | MGDG(36:3) | | C46H81O12 | | 0.010 | |  |
| 823.5577 | MGDG(18:2/18:2) | | C46H79O12 | | 0.008 | |  |
| 823.5577 | MGDG(18:2/18:2) | | C46H79O12 | | 0.034 | |  |
| 821.5421 | MGDG(18:2/18:3) | | C46H77O12 | | 0.001 | |  |
| 945.6673 | MGDG(45:6) | | C55H93O12 | | 0.004 | |  |
| **Total MGDG** | |  | |  | | **0.086** | |
| 509.4575 | OAHFA(16:0/16:0) | | C32H61O4 | | 0.008 | |  |
| 533.4575 | OAHFA(18:2/16:0) | | C34H61O4 | | 0.016 | |  |
| **Total OAHFA** | |  | |  | | **0.024** | |
| 645.4501 | PA(16:0/16:1) | | C35H66O8N0P1 | | 0.323 | |  |
| 645.4501 | PA(16:0/16:1) | | C35H66O8N0P1 | | 0.006 | |  |
| 645.4501 | PA(16:0/16:1) | | C35H66O8N0P1 | | 0.042 | |  |
| 645.4501 | PA(16:0/16:1) | | C35H66O8N0P1 | | 0.018 | |  |
| 645.4501 | PA(16:0/16:1) | | C35H66O8N0P1 | | 0.002 | |  |
| 645.4501 | PA(16:0/16:1) | | C35H66O8N0P1 | | 0.001 | |  |
| 645.4501 | PA(16:0/16:1) | | C35H66O8N0P1 | | 0.003 | |  |
| 645.4501 | PA(16:0/16:1) | | C35H66O8N0P1 | | 0.002 | |  |
| 645.4501 | PA(16:0/16:1) | | C35H66O8N0P1 | | 0.000 | |  |
| 645.4501 | PA(16:0/16:1) | | C35H66O8N0P1 | | 0.001 | |  |
| 659.4657 | PA(17:1/16:0) | | C36H68O8N0P1 | | 0.005 | |  |
| 659.4657 | PA(17:1/16:0) | | C36H68O8N0P1 | | 0.026 | |  |
| 659.4657 | PA(17:1/16:0) | | C36H68O8N0P1 | | 0.005 | |  |
| 659.4657 | PA(17:1/16:0) | | C36H68O8N0P1 | | 0.004 | |  |
| 659.4657 | PA(17:1/16:0) | | C36H68O8N0P1 | | 0.007 | |  |
| 659.4657 | PA(17:1/16:0) | | C36H68O8N0P1 | | 0.003 | |  |
| 659.4657 | PA(17:1/16:0) | | C36H68O8N0P1 | | 0.001 | |  |
| 659.4657 | PA(17:1/16:0) | | C36H68O8N0P1 | | 0.000 | |  |
| 659.4657 | PA(17:1/16:0) | | C36H68O8N0P1 | | 0.012 | |  |
| 659.4657 | PA(17:1/16:0) | | C36H68O8N0P1 | | 0.000 | |  |
| 673.4814 | PA(34:1) | | C37H70O8N0P1 | | 0.050 | |  |
| 671.4657 | PA(16:0/18:2) | | C37H68O8N0P1 | | 0.533 | |  |
| 671.4657 | PA(16:0/18:2) | | C37H68O8N0P1 | | 0.072 | |  |
| 671.4657 | PA(16:0/18:2) | | C37H68O8N0P1 | | 0.148 | |  |
| 671.4657 | PA(16:0/18:2) | | C37H68O8N0P1 | | 0.004 | |  |
| 671.4657 | PA(16:0/18:2) | | C37H68O8N0P1 | | 0.000 | |  |
| 671.4657 | PA(16:0/18:2) | | C37H68O8N0P1 | | 0.000 | |  |
| 671.4657 | PA(16:0/18:2) | | C37H68O8N0P1 | | 0.000 | |  |
| 671.4657 | PA(16:0/18:2) | | C37H68O8N0P1 | | 0.003 | |  |
| 671.4657 | PA(16:0/18:2) | | C37H68O8N0P1 | | 0.006 | |  |
| 671.4657 | PA(16:0/18:2) | | C37H68O8N0P1 | | 0.002 | |  |
| 671.4657 | PA(16:0/18:2) | | C37H68O8N0P1 | | 0.000 | |  |
| 671.4657 | PA(16:0/18:2) | | C37H68O8N0P1 | | 0.001 | |  |
| 671.4657 | PA(16:0/18:2) | | C37H68O8N0P1 | | 0.001 | |  |
| 671.4657 | PA(16:0/18:2) | | C37H68O8N0P1 | | 0.001 | |  |
| 671.4657 | PA(16:0/18:2) | | C37H68O8N0P1 | | 0.001 | |  |
| 671.4657 | PA(16:0/18:2) | | C37H68O8N0P1 | | 0.002 | |  |
| 671.4657 | PA(16:0/18:2) | | C37H68O8N0P1 | | 0.001 | |  |
| 671.4657 | PA(34:2) | | C37H68O8N0P1 | | 0.000 | |  |
| 669.4501 | PA(16:1/18:2) | | C37H66O8N0P1 | | 0.012 | |  |
| 669.4501 | PA(16:1/18:2) | | C37H66O8N0P1 | | 0.000 | |  |
| 685.4814 | PA(17:1/18:1) | | C38H70O8N0P1 | | 0.005 | |  |
| 699.497 | PA(36:2) | | C39H72O8N0P1 | | 0.005 | |  |
| 697.4814 | PA(18:1/18:2) | | C39H70O8N0P1 | | 0.001 | |  |
| 695.4657 | PA(18:2/18:2) | | C39H68O8N0P1 | | 0.244 | |  |
| 695.4657 | PA(18:2/18:2) | | C39H68O8N0P1 | | 0.006 | |  |
| 695.4657 | PA(18:2/18:2) | | C39H68O8N0P1 | | 0.000 | |  |
| 695.4657 | PA(18:2/18:2) | | C39H68O8N0P1 | | 0.002 | |  |
| 695.4657 | PA(18:2/18:2) | | C39H68O8N0P1 | | 0.004 | |  |
| 695.4657 | PA(18:2/18:2) | | C39H68O8N0P1 | | 0.000 | |  |
| **Total PA** |  | |  | | **1.568** | |  |
| 804.576 | PC(16:0/18:1) | | C43H83O10N1P1 | | 0.025 | |  |
| **Total PC** |  | |  | | **0.025** | |  |
| 634.4453 | PE(16:0/12:0) | | C33H65O8N1P1 | | 0.006 | |  |
| 640.3984 | PE(29:4) | | C34H59O8N1P1 | | 0.004 | |  |
| 662.4766 | PE(16:0/14:0) | | C35H69O8N1P1 | | 0.081 | |  |
| 660.461 | PE(16:1/14:0) | | C35H67O8N1P1 | | 0.033 | |  |
| 690.5079 | PE(16:0/16:0) | | C37H73O8N1P1 | | 0.152 | |  |
| 688.4923 | PE(16:0/16:1) | | C37H71O8N1P1 | | 3.936 | |  |
| 686.4766 | PE(16:1/16:1) | | C37H69O8N1P1 | | 0.133 | |  |
| 702.5079 | PE(17:1/16:0) | | C38H73O8N1P1 | | 1.114 | |  |
| 700.4923 | PE(17:1/16:1) | | C38H71O8N1P1 | | 0.193 | |  |
| 718.5392 | PE(18:0/16:0) | | C39H77O8N1P1 | | 0.006 | |  |
| 716.5236 | PE(16:0/18:1) | | C39H75O8N1P1 | | 0.424 | |  |
| 714.5079 | PE(16:0/18:2) | | C39H73O8N1P1 | | 0.006 | |  |
| 714.5079 | PE(16:1/18:1) | | C39H73O8N1P1 | | 2.006 | |  |
| 714.5079 | PE(17:1/17:1) | | C39H73O8N1P1 | | 0.123 | |  |
| 712.4923 | PE(34:3) | | C39H71O8N1P1 | | 0.005 | |  |
| 712.4923 | PE(16:1/18:2) | | C39H71O8N1P1 | | 0.025 | |  |
| 730.5392 | PE(19:1/16:0) | | C40H77O8N1P1 | | 0.023 | |  |
| 728.5236 | PE(17:1/18:1) | | C40H75O8N1P1 | | 0.282 | |  |
| 726.5079 | PE(35:3) | | C40H73O8N1P1 | | 0.007 | |  |
| 744.5549 | PE(18:0/18:1) | | C41H79O8N1P1 | | 0.006 | |  |
| 742.5392 | PE(18:1/18:1) | | C41H77O8N1P1 | | 0.142 | |  |
| 742.5392 | PE(17:1/19:1) | | C41H77O8N1P1 | | 0.019 | |  |
| 740.5236 | PE(18:1/18:2) | | C41H75O8N1P1 | | 0.005 | |  |
| 740.5236 | PE(18:1/18:2) | | C41H75O8N1P1 | | 0.047 | |  |
| 738.5079 | PE(18:2/18:2) | | C41H73O8N1P1 | | 0.035 | |  |
| 738.5079 | PE(18:2/18:2) | | C41H73O8N1P1 | | 0.018 | |  |
| 756.5549 | PE(19:1/18:1) | | C42H79O8N1P1 | | 0.010 | |  |
| 766.5392 | PE(18:0/20:4) | | C43H77O8N1P1 | | 0.006 | |  |
| 798.6018 | PE(22:0/18:2) | | C45H85O8N1P1 | | 0.006 | |  |
| 812.6175 | PE(18:2/23:0) | | C46H87O8N1P1 | | 0.007 | |  |
| 826.6331 | PE(24:0/18:2) | | C47H89O8N1P1 | | 0.008 | |  |
| 824.6175 | PE(24:1/18:2) | | C47H87O8N1P1 | | 0.007 | |  |
| **Total PE** |  | |  | | **8.876** | |  |
| 693.4712 | PG(16:0/14:0) | | C36H70O10N0P1 | | 0.012 | |  |
| 691.4556 | PG(16:1/14:0) | | C36H68O10N0P1 | | 0.003 | |  |
| 721.5025 | PG(16:0/16:0) | | C38H74O10N0P1 | | 0.006 | |  |
| 721.5025 | PG(16:0/16:0) | | C38H74O10N0P1 | | 0.047 | |  |
| 719.4869 | PG(16:0/16:1) | | C38H72O10N0P1 | | 0.006 | |  |
| 719.4869 | PG(16:0/16:1) | | C38H72O10N0P1 | | 0.476 | |  |
| 717.4712 | PG(16:1/16:1) | | C38H70O10N0P1 | | 0.008 | |  |
| 717.4712 | PG(16:1/16:1) | | C38H70O10N0P1 | | 0.013 | |  |
| 733.5025 | PG(17:1/16:0) | | C39H74O10N0P1 | | 0.073 | |  |
| 747.5182 | PG(16:0/18:1) | | C40H76O10N0P1 | | 0.086 | |  |
| 745.5025 | PG(16:1/18:1) | | C40H74O10N0P1 | | 0.005 | |  |
| 745.5025 | PG(16:1/18:1) | | C40H74O10N0P1 | | 0.196 | |  |
| 743.4869 | PG(16:1/18:2) | | C40H72O10N0P1 | | 0.004 | |  |
| 759.5182 | PG(17:1/18:1) | | C41H76O10N0P1 | | 0.019 | |  |
| 773.5338 | PG(18:1/18:1) | | C42H78O10N0P1 | | 0.011 | |  |
| 769.5025 | PG(18:2/18:2) | | C42H74O10N0P1 | | 0.006 | |  |
| **Total PG** |  | |  | | **0.969** | |  |
| 833.5186 | PI(16:0/18:2) | | C43H78O13N0P1 | | 0.016 | |  |
| **Total PI** |  | |  | | **0.016** | |  |
| 995.5631 | PIP(40:3) | | C49H89O16N0P2 | | 0.010 | |  |
| 1109.704 | PIP(48:2) | | C57H107O16N0P2 | | 0.031 | |  |
| **Total PIP** |  | |  | | **0.041** | |  |
| 716.4872 | PS(32:1p) | | C38H71O9N1P1 | | 0.008 | |  |
| 754.4665 | PS(34:4) | | C40H69O10N1P1 | | 0.011 | |  |
| 796.5134 | PS(37:4) | | C43H75O10N1P1 | | 0.060 | |  |
| 808.5134 | PS(38:5) | | C44H75O10N1P1 | | 0.009 | |  |
| **Total PS** |  | |  | | **0.087** | |  |
| 817.5141 | SQDG(34:2) | | C43H77O12S1 | | 0.055 | |  |
| 855.457 | SQDG(34:6) | | C44H71O14S1 | | 0.038 | |  |
| 841.5141 | SQDG(36:4) | | C45H77O12S1 | | 0.015 | |  |
| 881.4727 | SQDG(36:7) | | C46H73O14S1 | | 0.016 | |  |
| 961.5353 | SQDG(42:9) | | C52H81O14S1 | | 0.300 | |  |
| **Total SQDG** | |  | |  | | **0.424** | |

Lipids abbreviations:

LPC： lysophosphatidylcholine PC： phosphatidylcholine；

LPE： lysophosphatidylethanolamine PE： phosphatidylethanolamine

LPS： lysophosphatidylserine PS： phosphatidylserine

LPG： lysophosphatidylglycerol PG： phosphatidylglycerol

LPI： lysophosphatidylinositol PI： phosphatidylinositol

PA： phosphatidic acid CL： Cardiolipin

Cer： Ceramides DGMG： Digalactosylmonoacylglycerol

DGDG： Digalactosyldiacylglycerol SQMG：Sulfoquinovosylmonoacylglycerol；

SQDG Sulfoquinovosyldiacylglycerol
