# Supplementary material for: Ginseng-derived nanoparticles alter macrophage polarization to inhibit melanoma growth
Source: J Immunother Cancer. 2019 Nov 27;7:326. doi: 10.1186/s40425-019-0817-4 (PMC6882204; doi:10.1186/s40425-019-0817-4)
Supplement: Supplementary file 2 — Additional file 2: Figure S1. Analysis of composition of GDNPs. Figure S2. Biodistribution and stability of GDNPs In vivo. Figure S3. C57BL/6 mice were treated with i.p. injection of DiR-lablled GDNPs in the presence or absence of clodronate liposomes. Figure S4. Biocompatibility of GDNPs in vitro and in vivo. Figure S5. FACS analysis of related surface markers of macrophages treated with nanoparticles from different plants. Figure S6. Uptake of GDNPs by macrophages depends on phagocytosis. Figure S7. GDNPs inhibit melanoma tumor growth in vivo. Figure S8. Mean tumor volume of subcutaneous B16F10 tumors in GDNPs versus PBS-treated mice with or without CD8+ T cell depletion by anti-CD8 antibody. Figure S9. Flow cytometry gating strategy for polarization analysis of tumor associated macrophages (TAMs). [file 40425_2019_817_MOESM2_ESM.docx]

**Additional file 2**

**a**


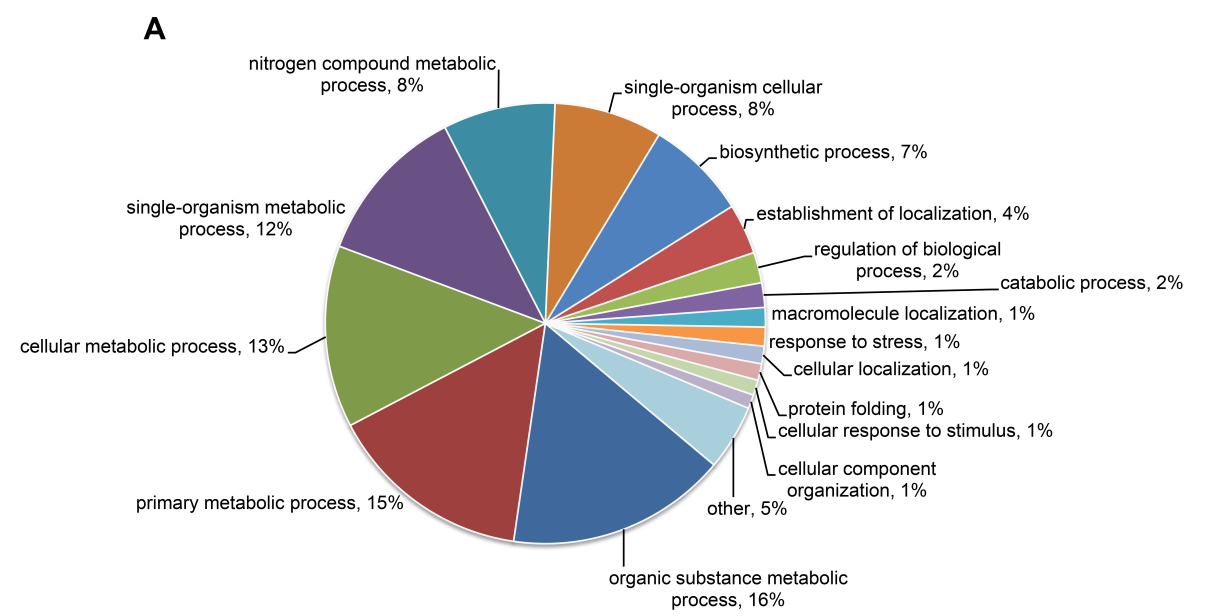


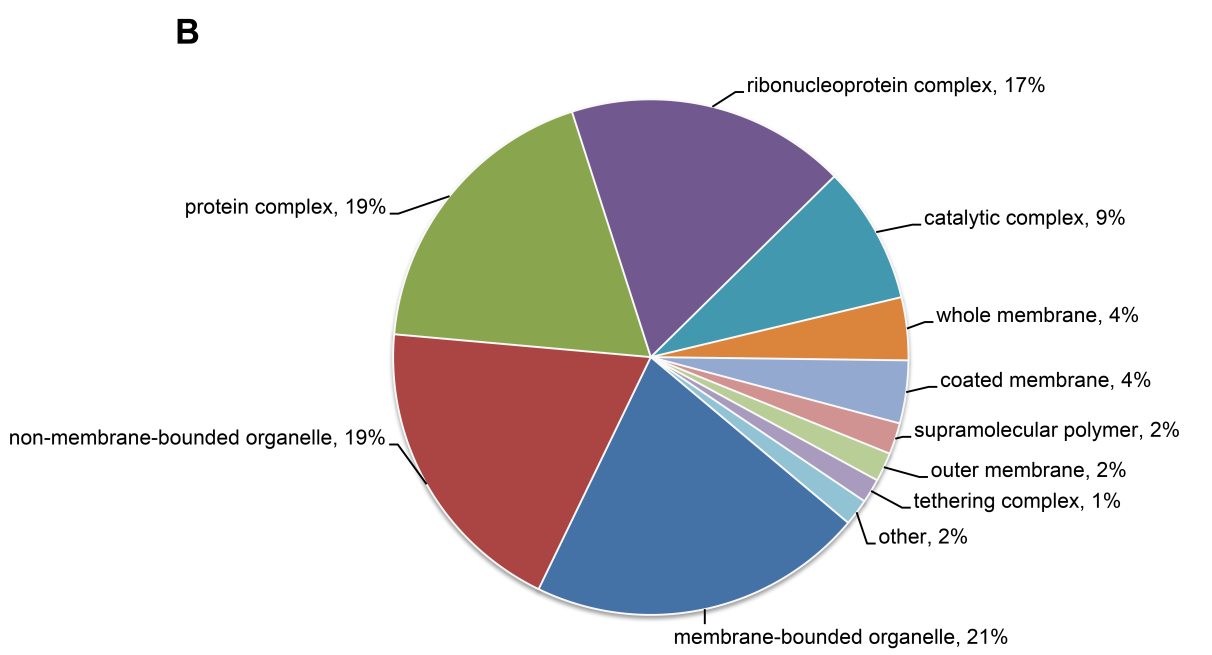


**c**

**b**


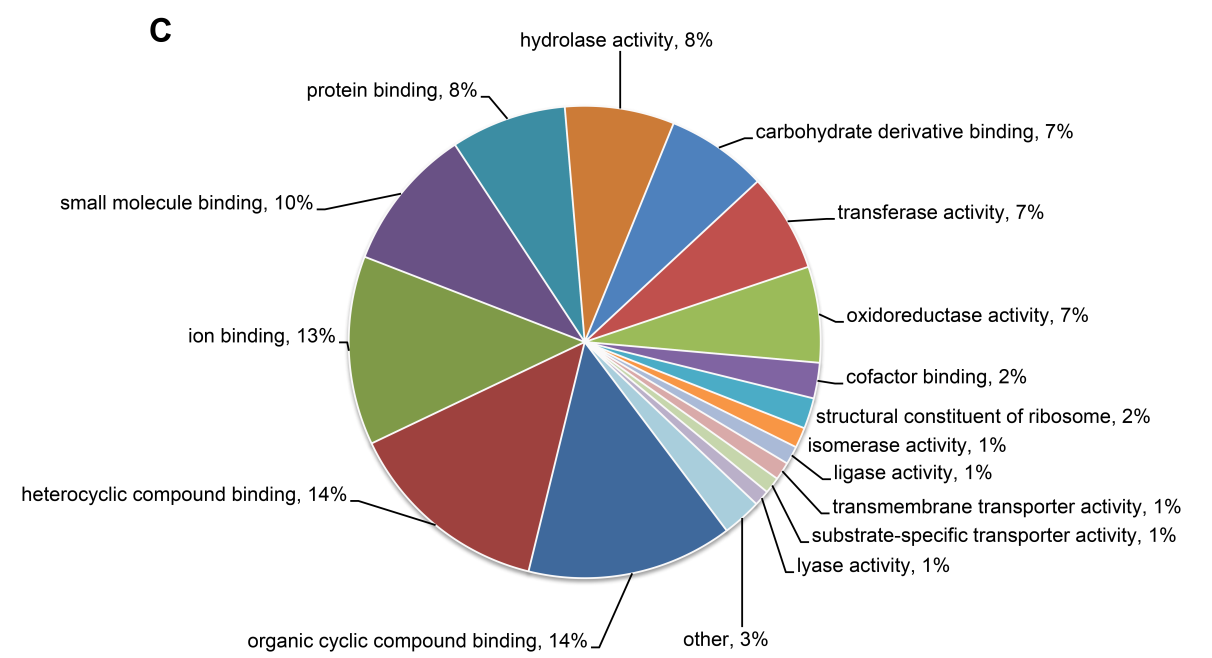


**Figure S1.** Analysis of composition of GDNPs. The GDNP proteome was categorized based on GO terms through the Uniprot-GOA database (http://www.ebi.ac.uk/GOA/). **a** Cellular component. **b** Molecular function. **c** Biological process.

**s.c.**

**i.v.**

**i.g.**

**Control**

**i.p.**


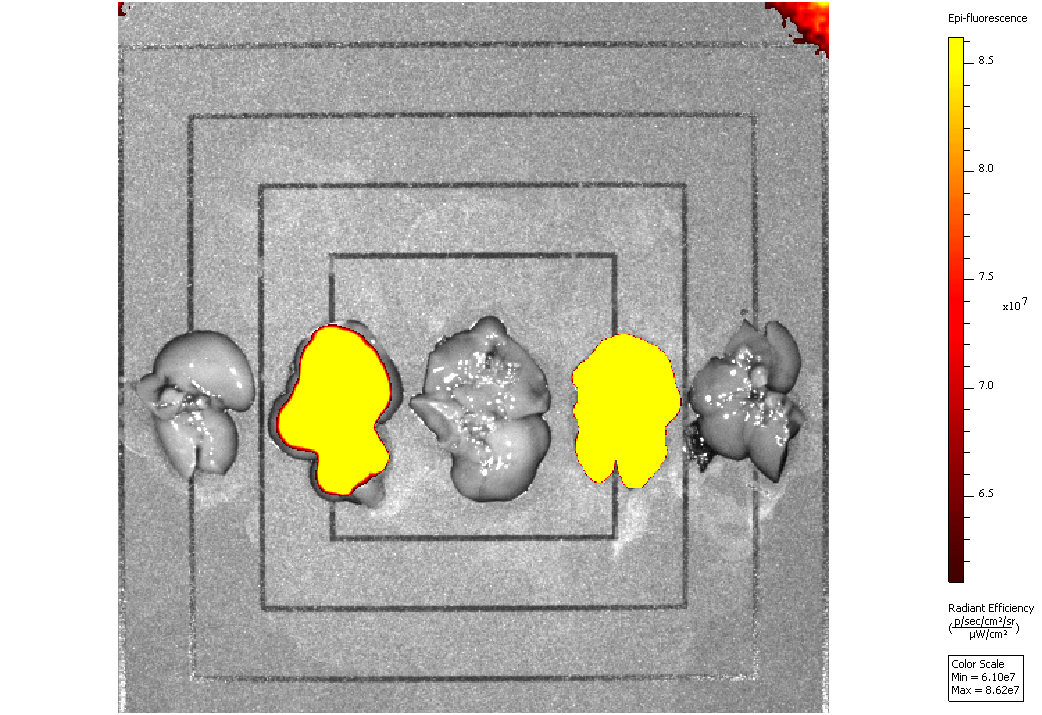

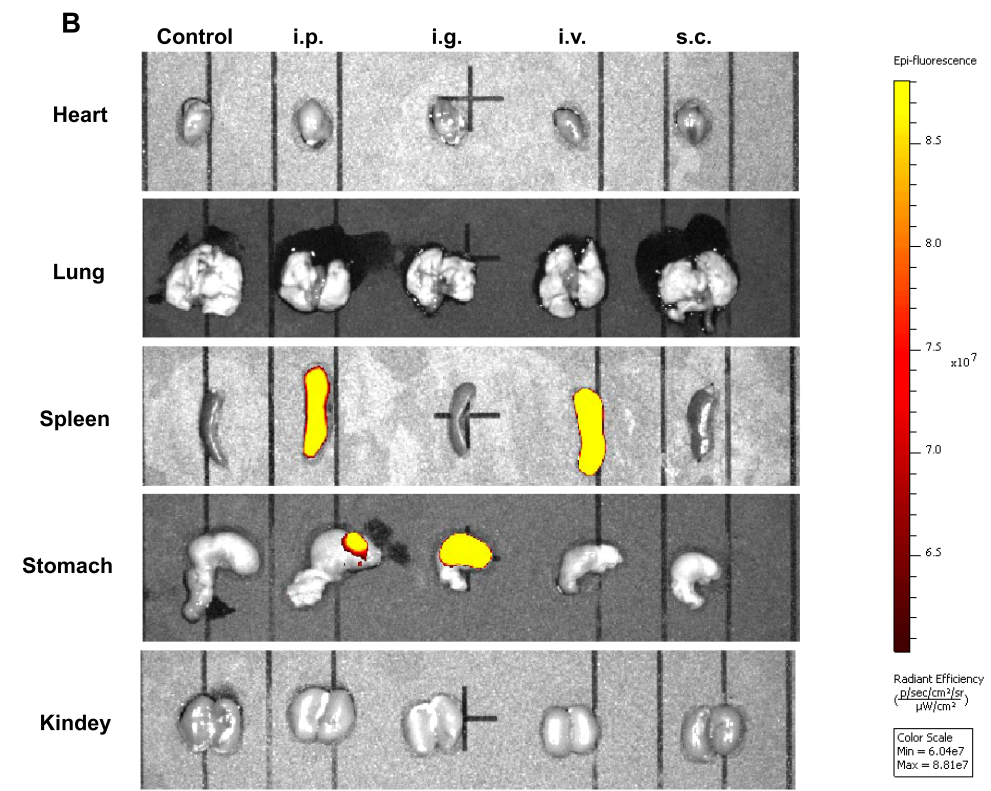


**Heart**

**Lung**

**Spleen**

**a**

**Stomach**

**Kidney**


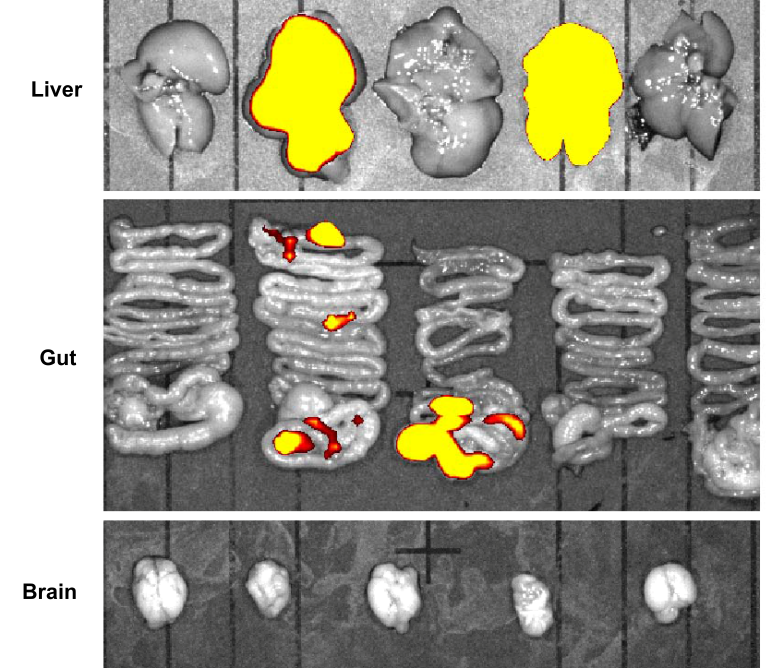


**Liver**

**Gut**

**Brain**


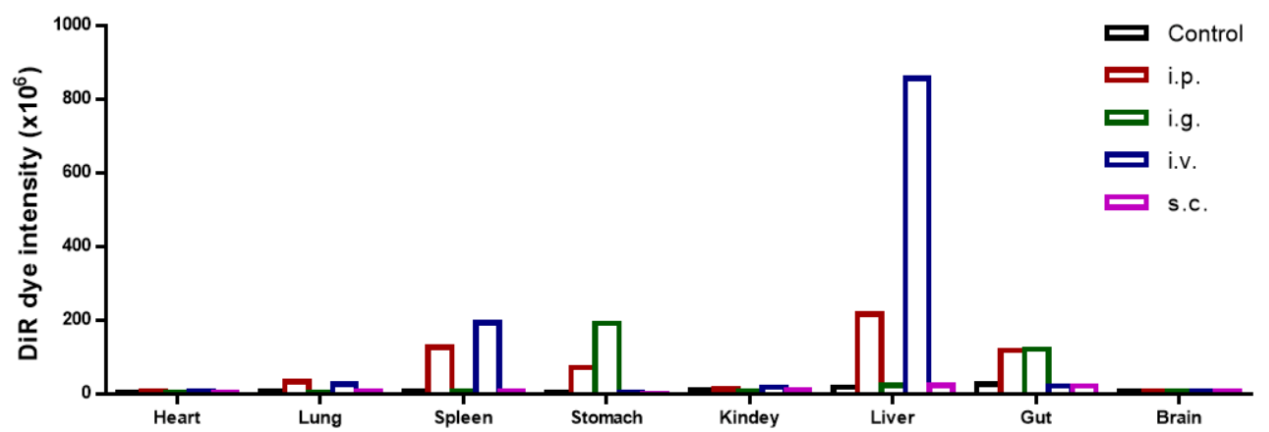


**c**

**b**

**
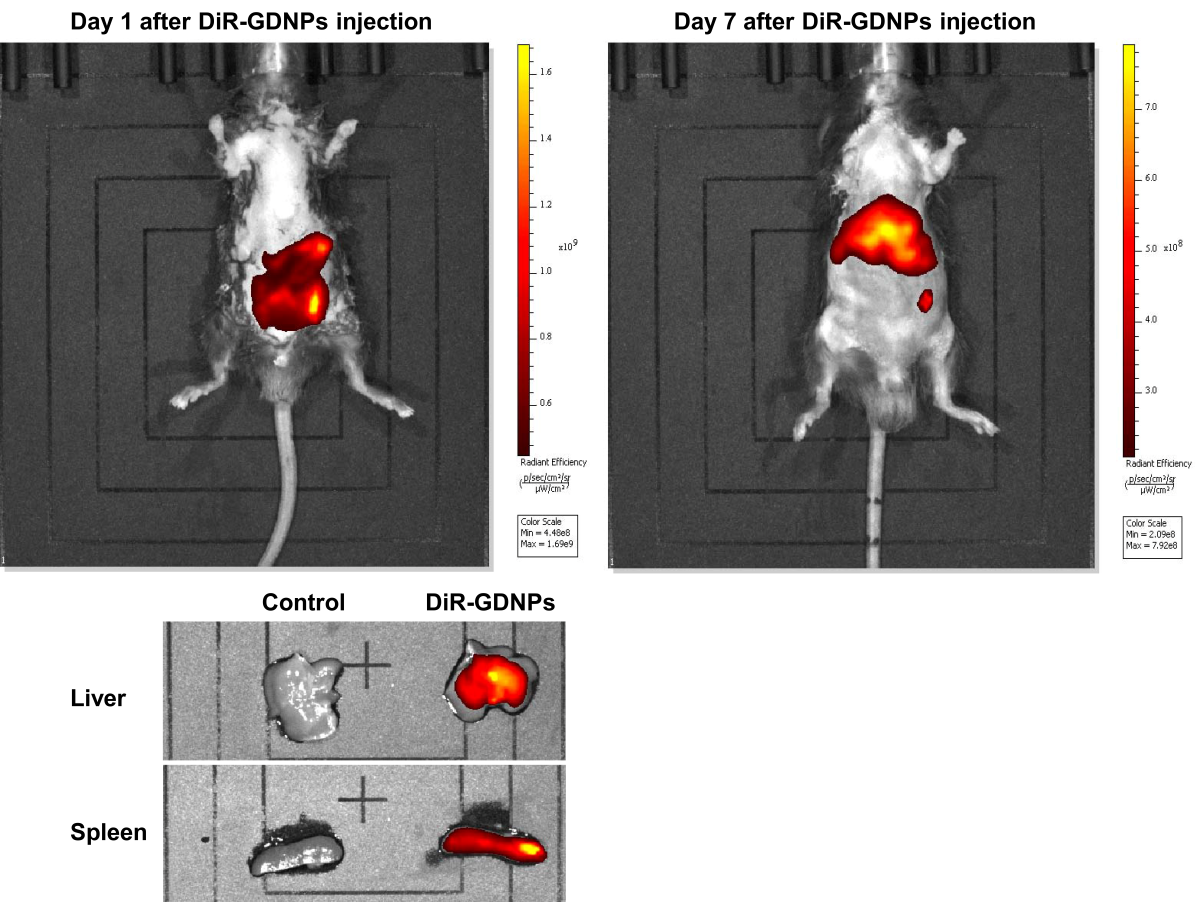
**

**
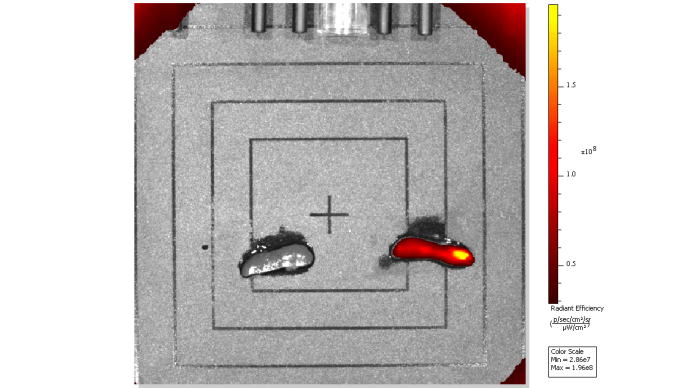

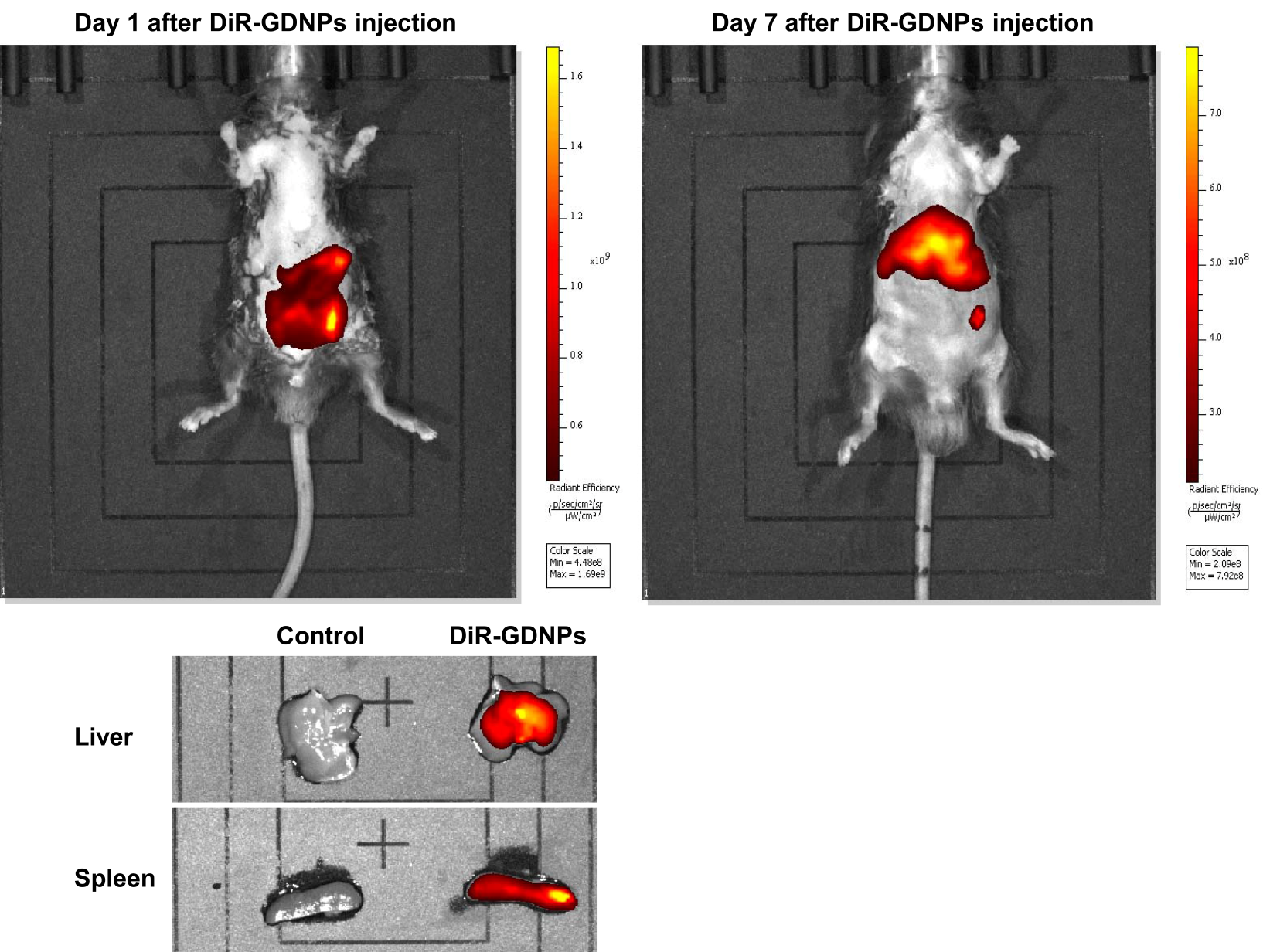
**

**Figure S2.** Biodistribution and stability of GDNPs *In vivo*. **a** *In vivo* biodistribution of DiR-labelled GDNPs administrated intraperitoneally (i.p.), intragastrically (i.g.), intravenously (i.v.) and subcutaneously (s.c.) to mice for 72 h. **b** Corresponding quantification of accumulated fluorescence signals in different organs (*n* = 3). **c** *In vivo* stability of GDNPs was determined by scanning (IVIS series) mice that received an i.p. injection of DiR-GDNPs.


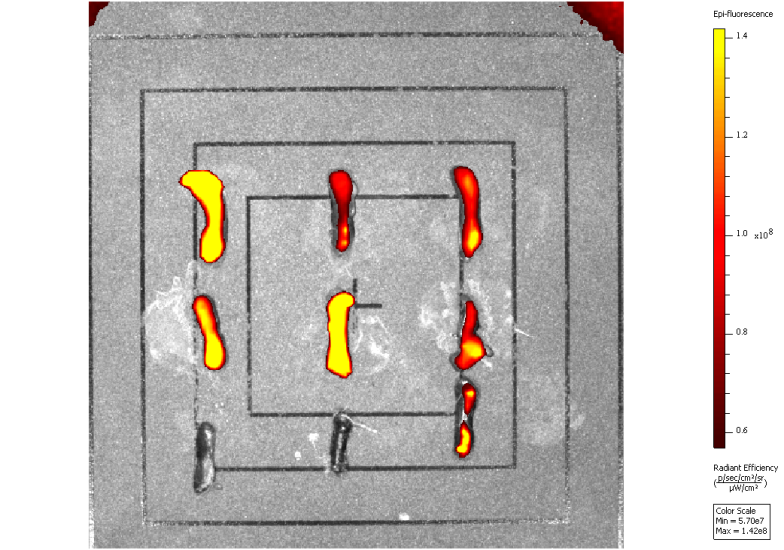

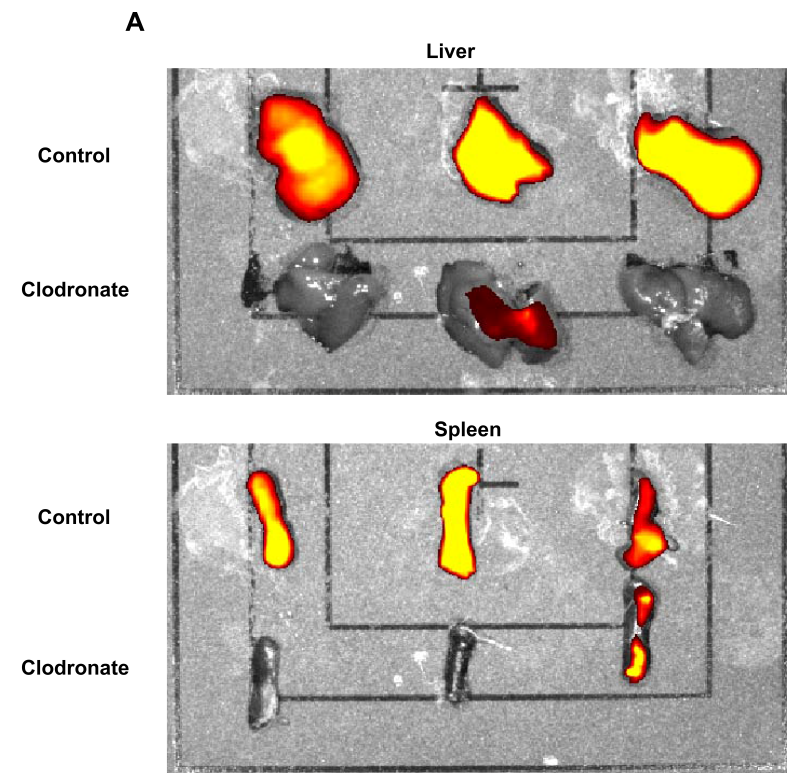

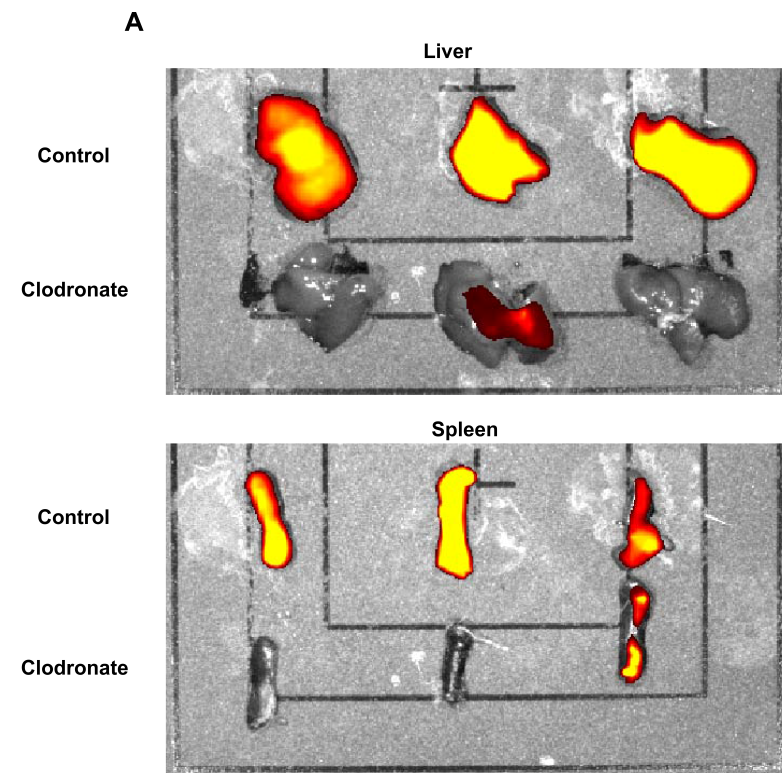


**a**


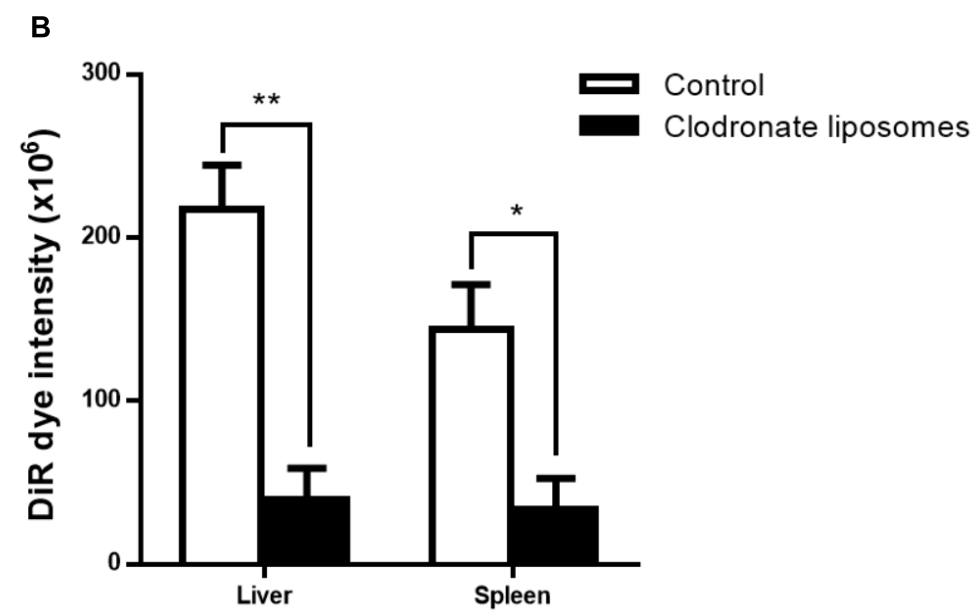


**b**

**Figure S3.** C57BL/6 mice were treated with i.p. injection of DiR-lablled GDNPs in the presence or absence of clodronate liposomes. **a** Biodistribution of DiR-lablled GDNPs in livers and spleens at 72 h following administration. **b** Corresponding quantification of accumulated fluorescence signals in liver and spleens (*n* = 3). All results represent the mean ± SEM. **P* < 0.05, ***P* < 0.01 compared with control; evaluated using Student’s *t* test (b).


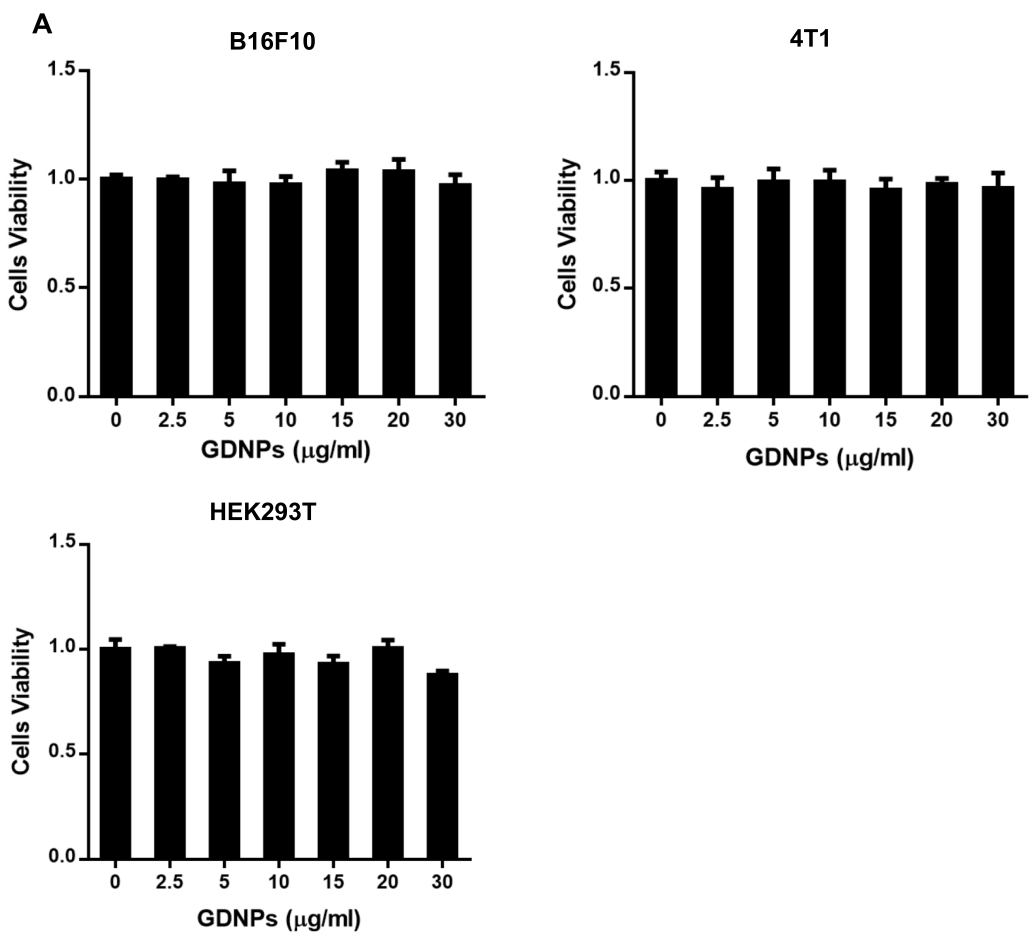

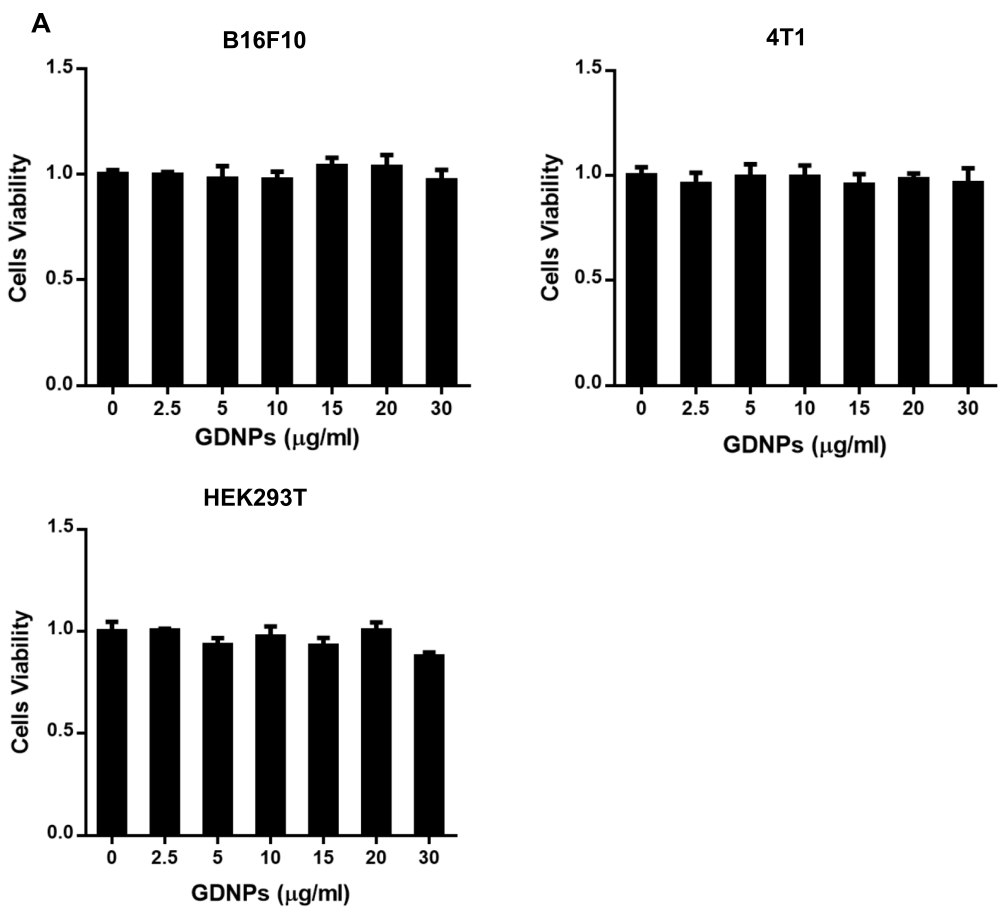


**a**

**A**

**b**


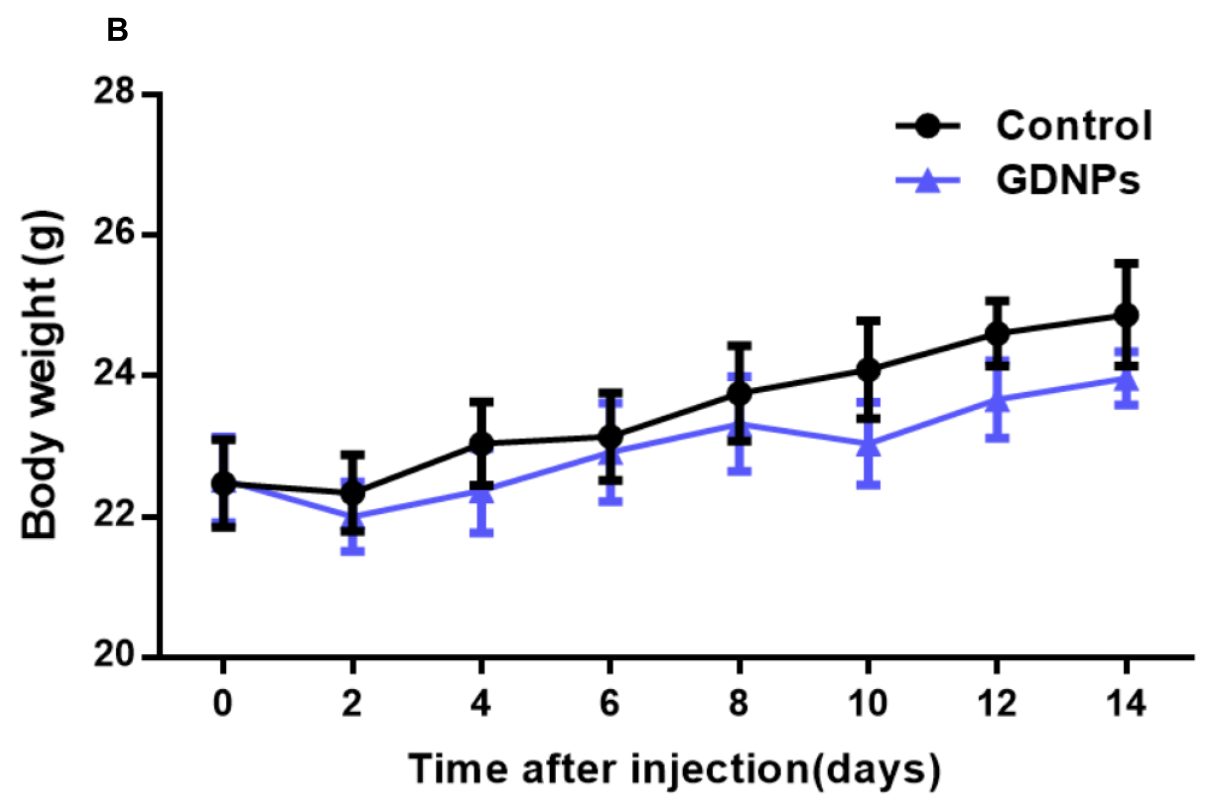


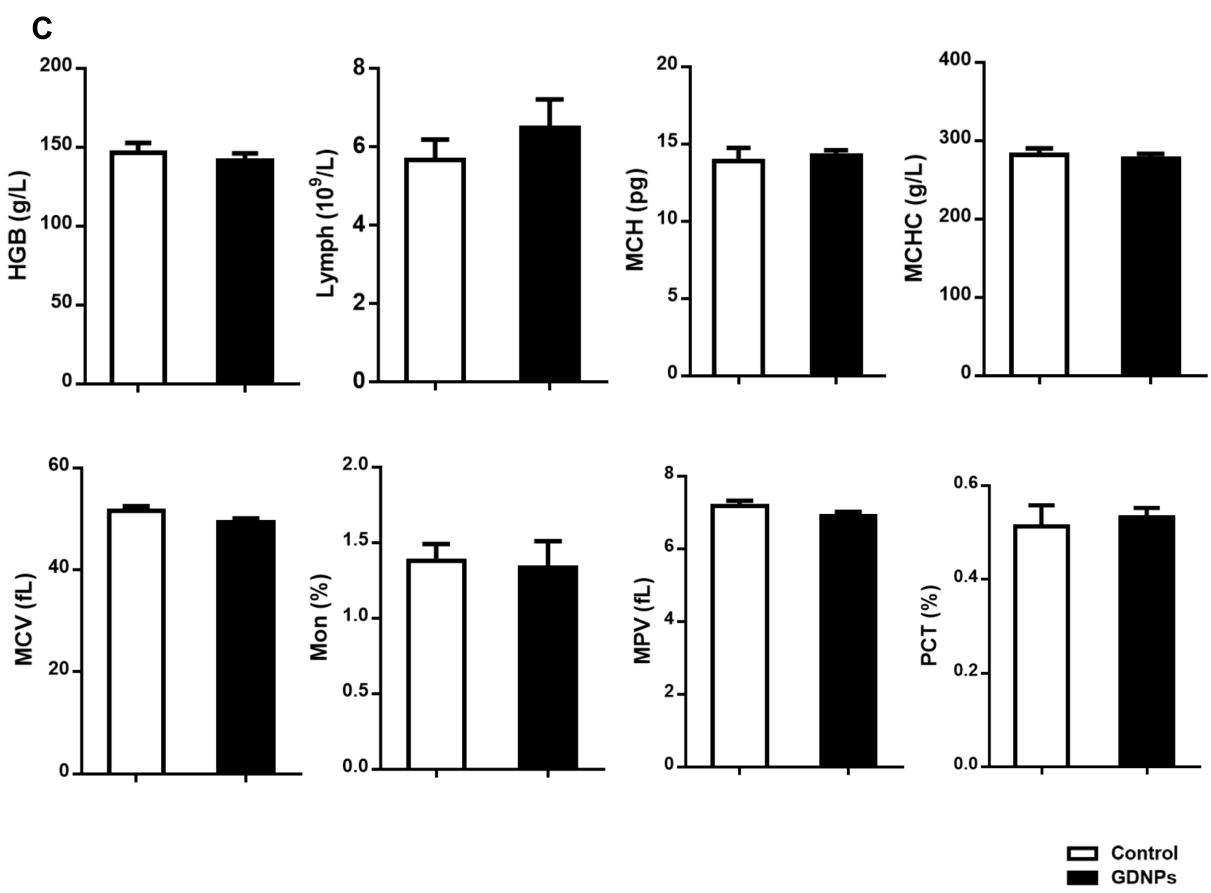


**c**


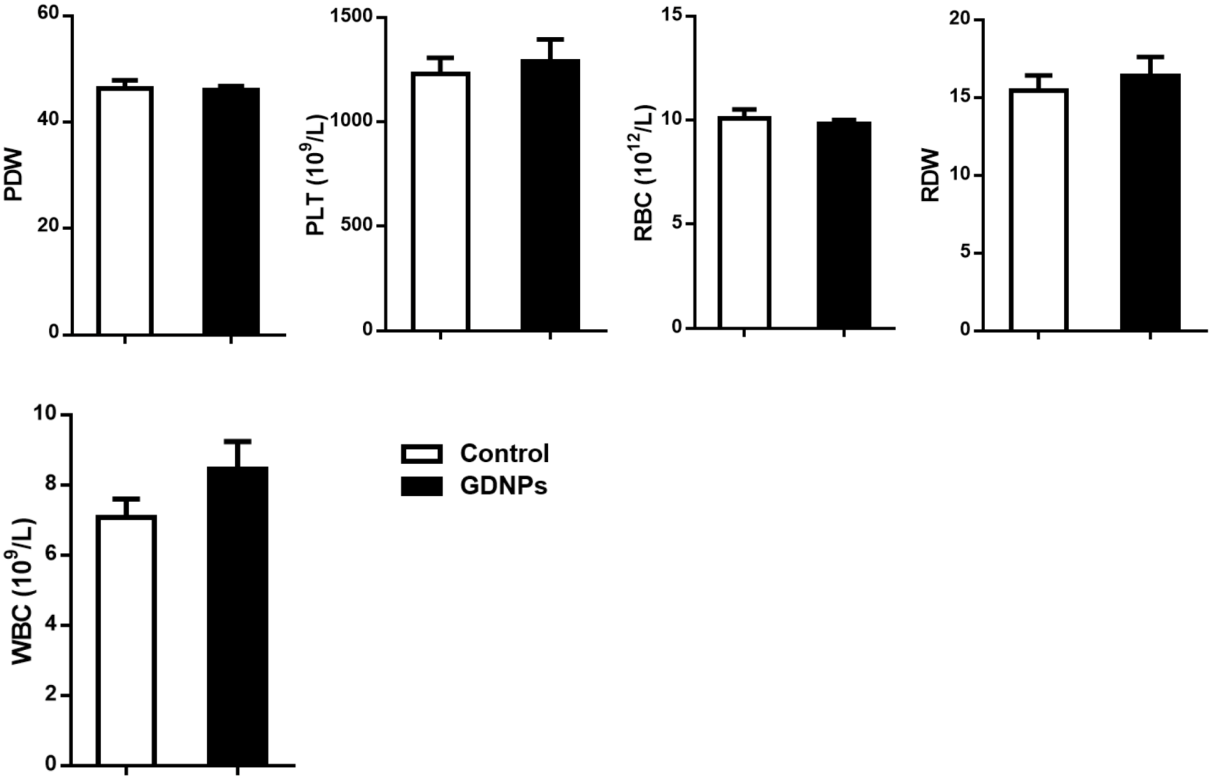


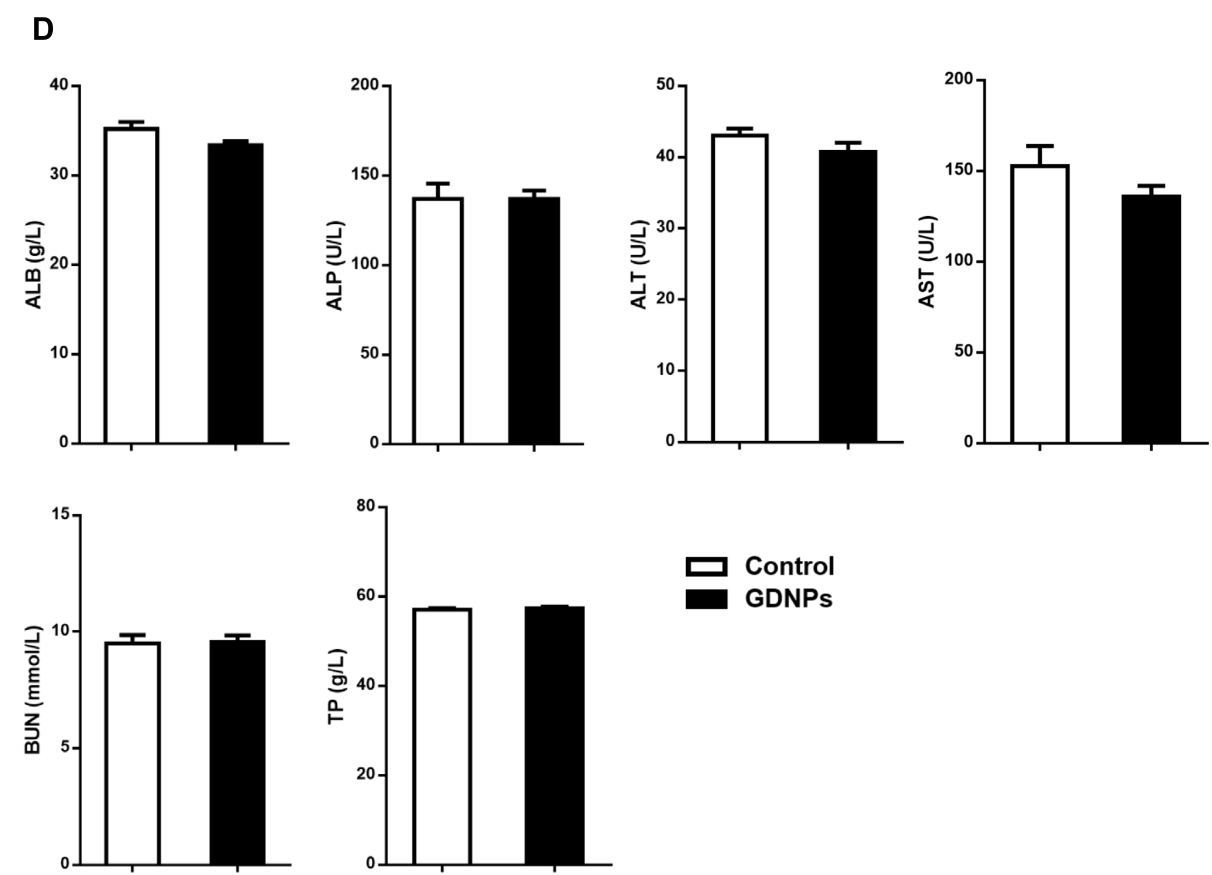


**d**


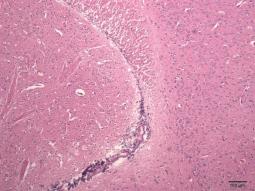

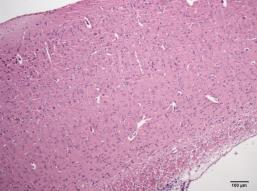


**GDNPs**

**Control**

**e**

**Brain**


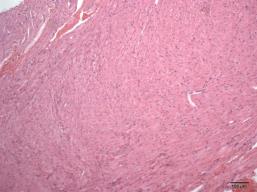

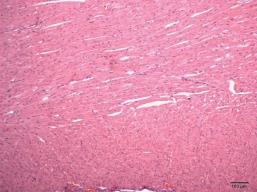


**Heart**


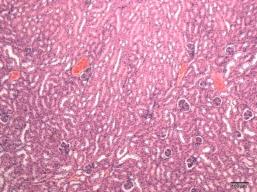

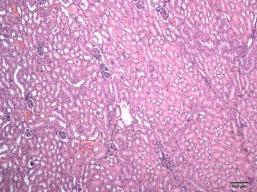


**Kidney**


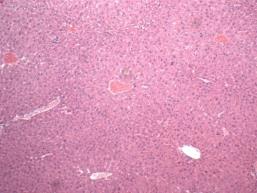

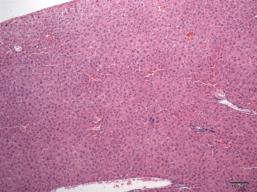


**Liver**


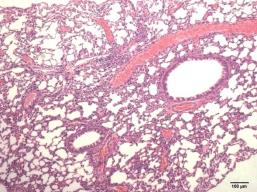

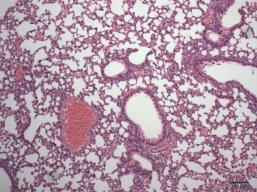


**Lungs**


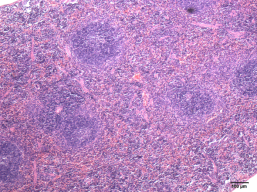

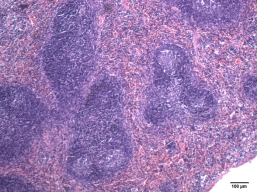


**Spleen**

**Figure** **S4.** Biocompatibility of GDNPs *in vitro* and *in vivo*. **a** Viability of several malignant (B16F10 cell line, mouse melanoma cell; 4T1 cell line, mouse mammary carcinoma cell) and non-malignant cell (HEK293T cell line, human embtyonic kidney cell) types after exposure to increased concentrations of GDNPs for 48 h. **b** Body weights of mice after intraperitoneal injection with GDNPs (100 μl, 250 μg/ml). **c** Whole blood cell analysis of mice after injection of GDNPs. HGB, haemoglobin; Lymph, lymphocyte; MCH, mean corpuscular haemoglobin; MCHC, Mean corpuscular haemoglobin concentration; MCV, mean corpuscular volume; Mon, monocyte; MPV, mean platelet volume; PCT, plateletcrit; PDW, platelet distribution width; PLT, platelets; RBC, red blood cell; RDW, red blood cell distribution width; WBC, white blood cell;. **d** The hepatic and renal functions of mice after injection of GDNPs were determined by blood chemistry tests. ALB, albumin; ALP, alkaline phosphatase; ALT, alanine aminotransferase; AST, aspartate aminotransferase; BUN, blood urea nitrogen; TP, total protein. **e** H&E staining of major organs harvested from mice injected with PBS and GDNPs (Scale bar = 100 μm). A representative image from each group of mice is shown.

**kindey**

**Spleen**

**CD80**


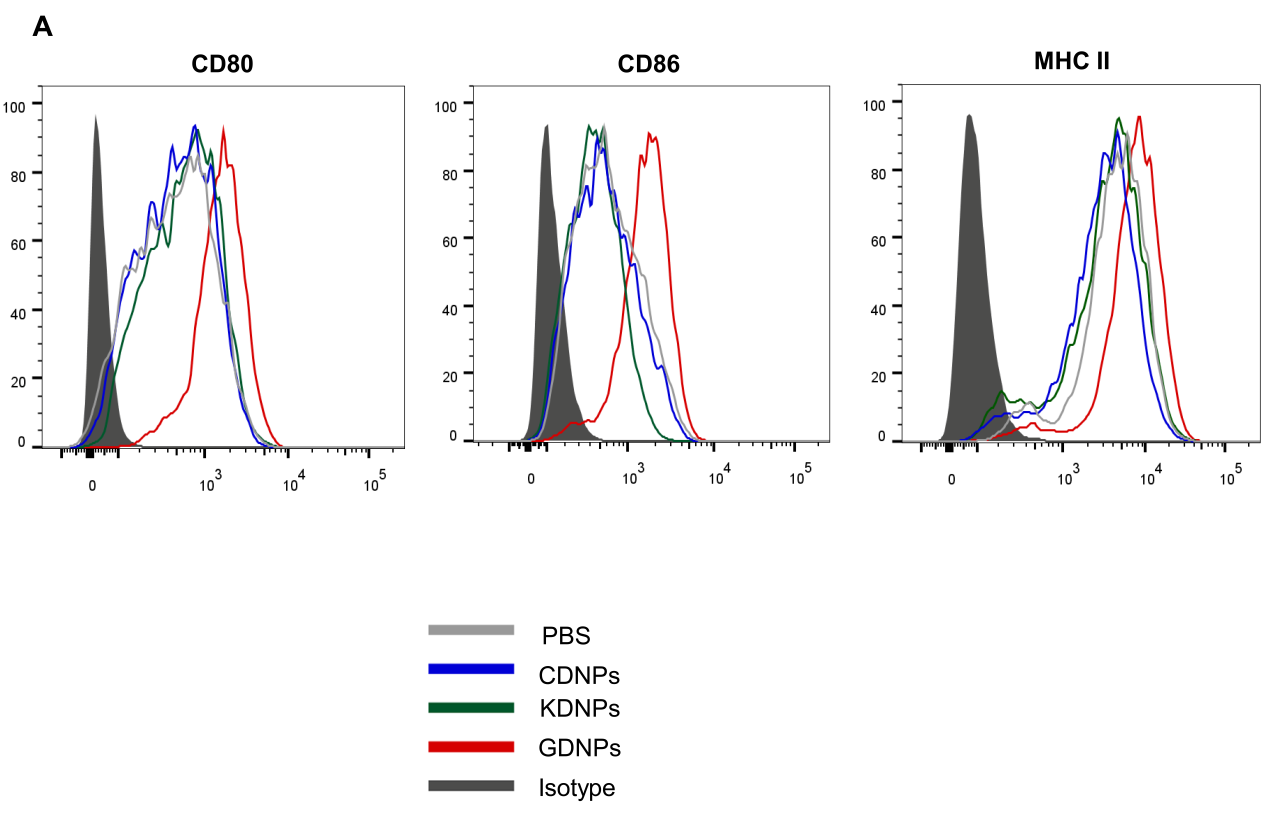


**MHC-II**

**CD86**


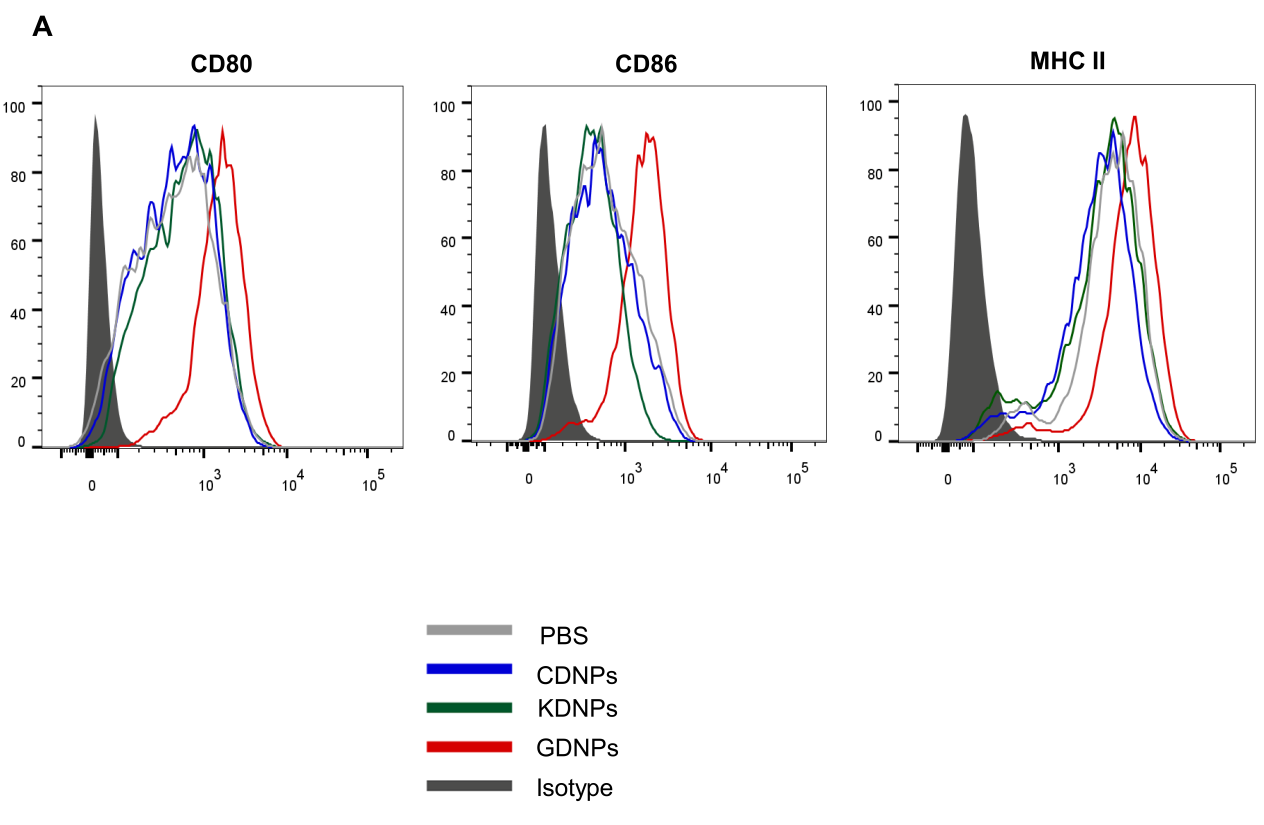


**Figure** **S5.** FACS analysis of related surface markers of macrophages treated with nanoparticles from different plants. BMDMs were incubated with EVs-like nanoparticles (10 μg/ml) derived from ginseng (GDNPs), cucumber (CDNPs) and kiwi fruit (KDNPs) for 48 h. The expression of M1-related surface markers on macrophages was analysed by FACS.

**DiI-GDNPs + LY294002**

**DiI-GDNPs + EIPA**

**DiI-GDNPs only**

**a**


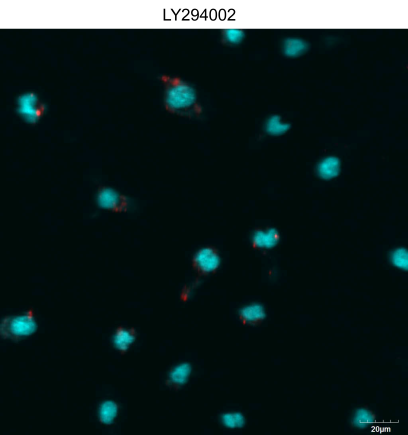

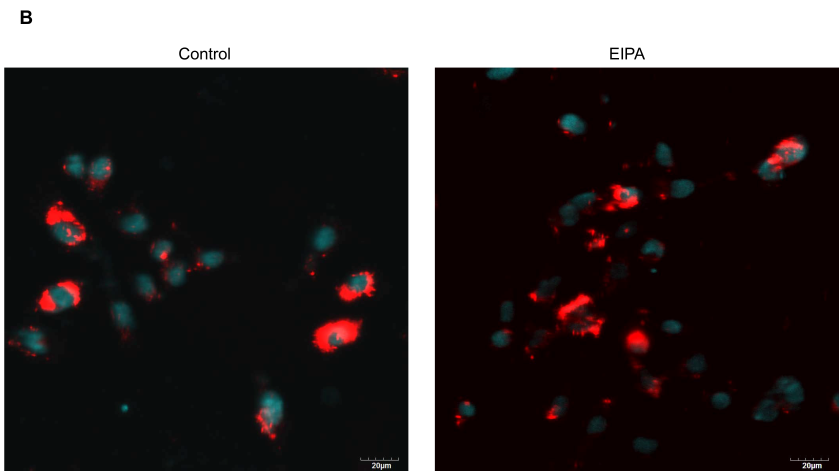


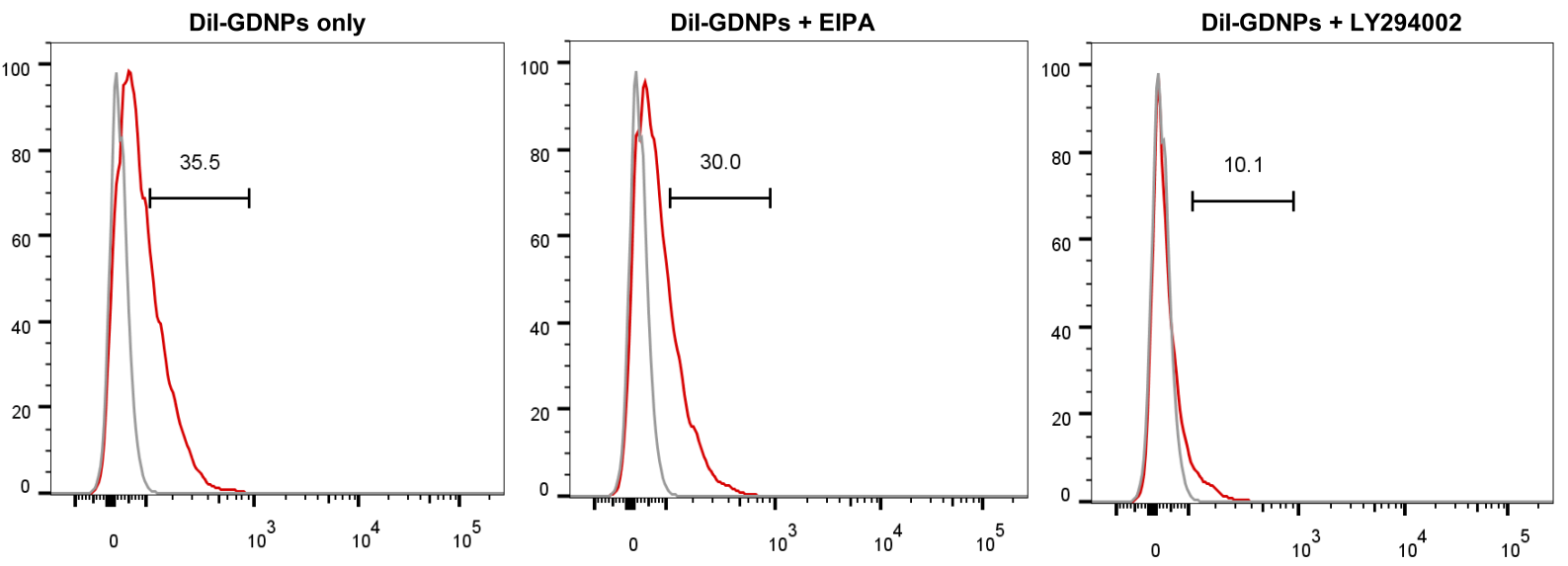


**DiI-GDNPs only**

**DiI-GDNPs + EIPA**

**DiI-GDNPs + LY294002**

**b**


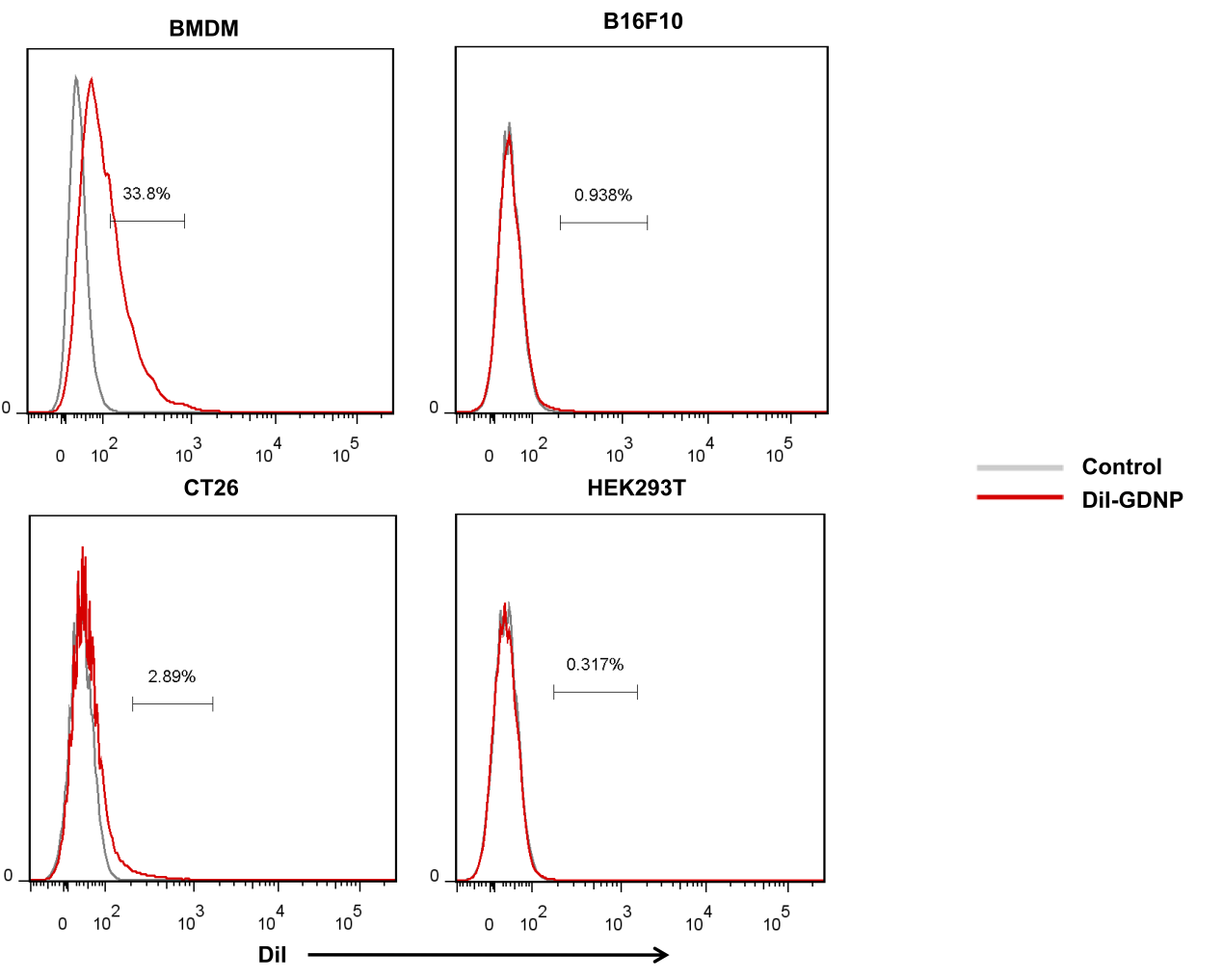


**DiI**





**c**

**CD80**

**MHC-II**

**CD86**


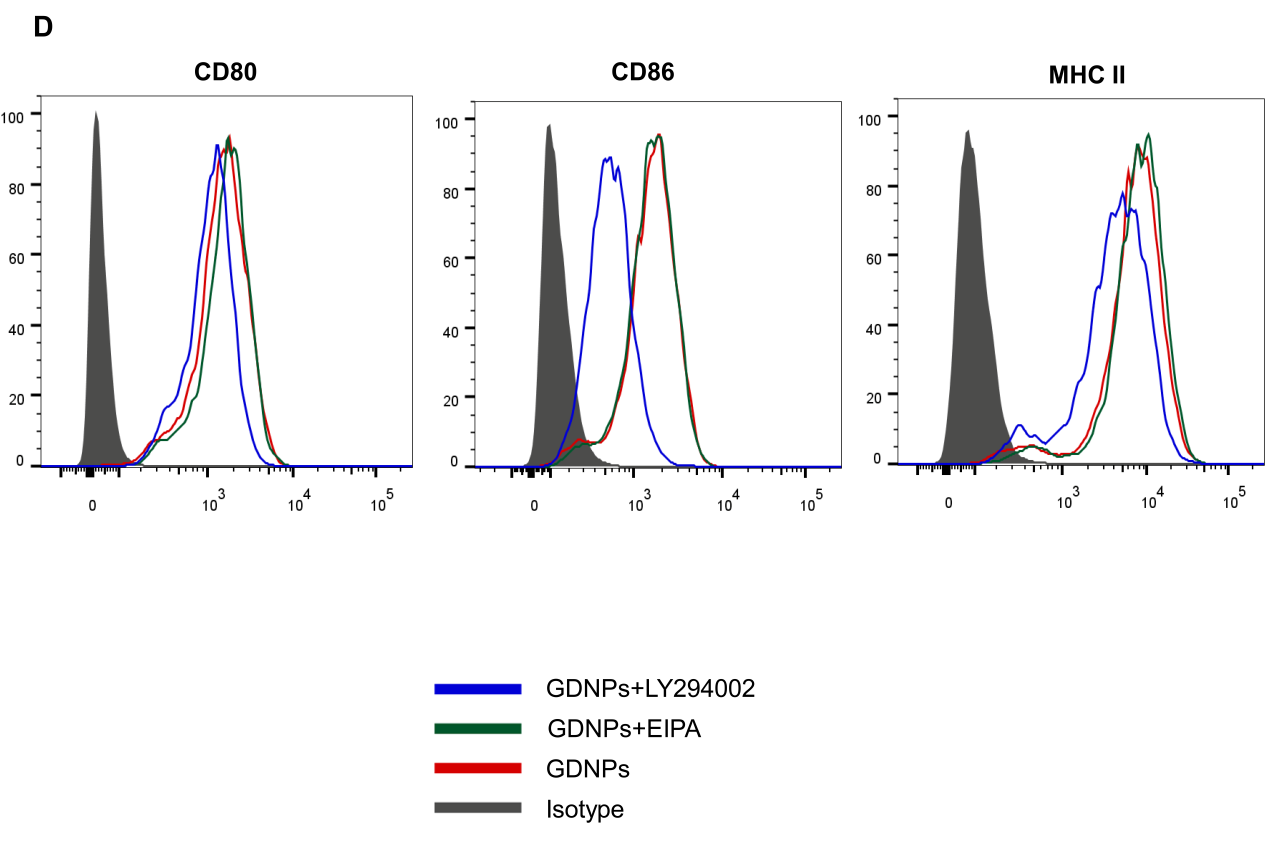

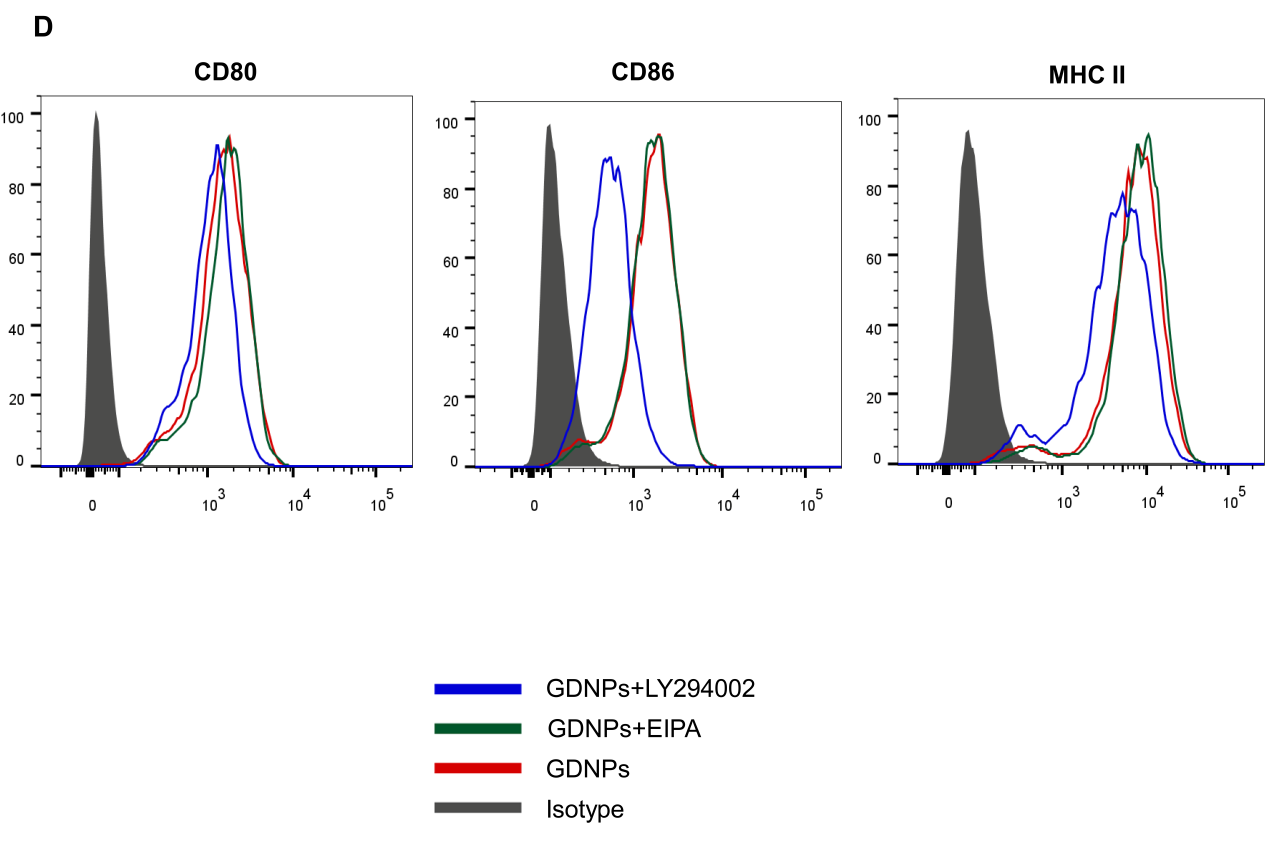


**GDNPs only**

**GDNPs + LY294002**

**Isotype**

**GDNPs + EIPA**

**d**

**Figure S6.** Uptake of GDNPs by macrophages depends on phagocytosis. **a** Confocal images of DiI-labelled GDNPs taken up by macrophages in the presence of EIPA (10 μM) or LY294002 (30 μM). **b** Effect of EIPA and LY294002 on DiI-GDNPs uptake (12 h) monitored by FACS analysis. **c** Internalization of the DiI-GDNPs was quantified based on the FACS analysis (*n* = 3). **d** The expression of related surface markers on macrophages was analysed by FACS. The results represent the mean ± SEM. ****P* < 0.001 compared with M0 + DiI-GDNPs; evaluated using Student’s *t* test (c).

**a**


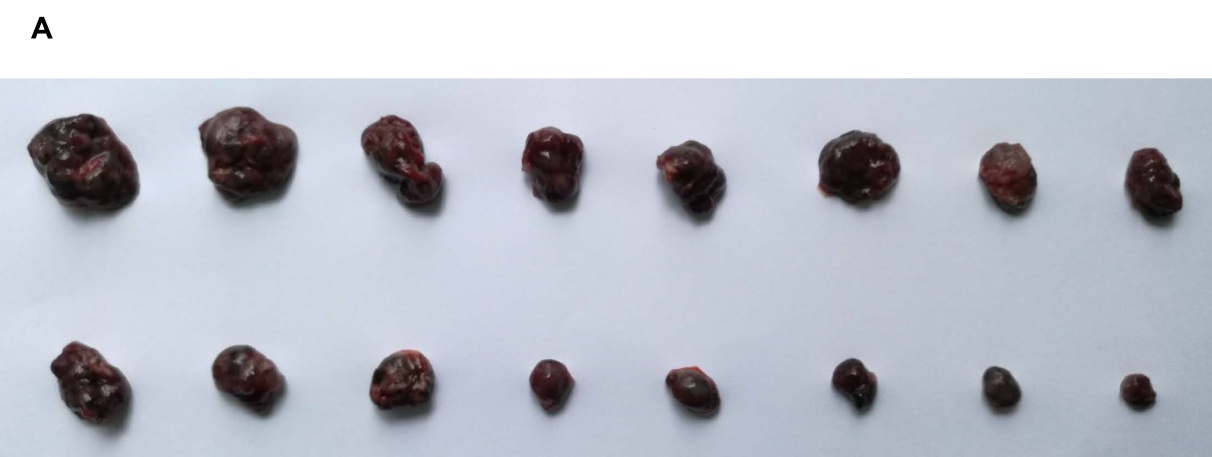


**b**

**
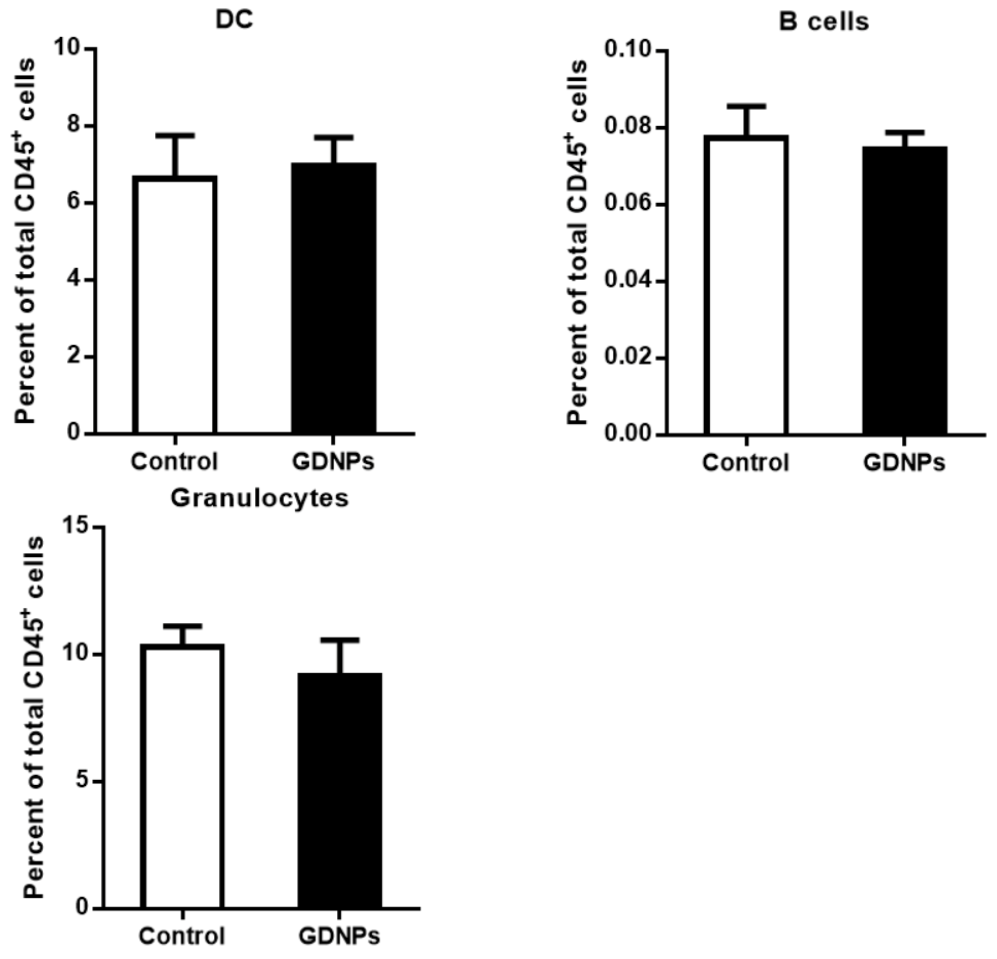
**

**
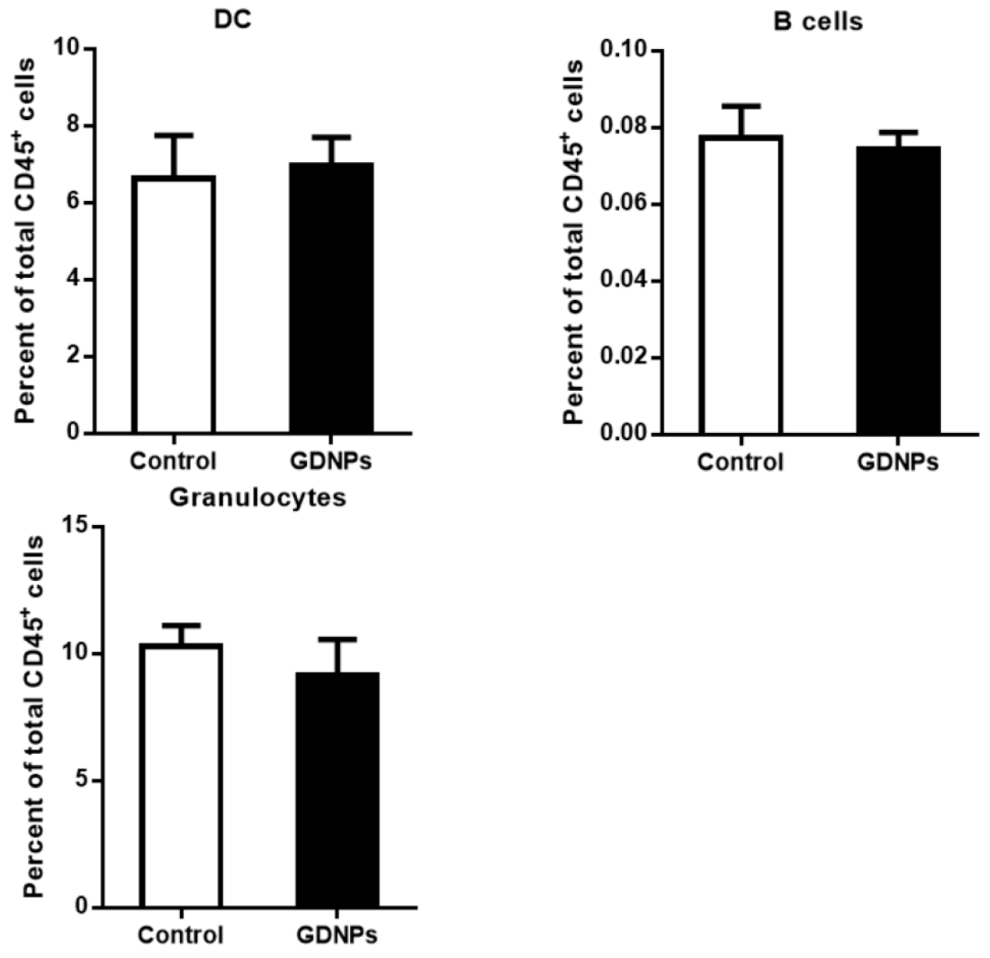
**

**c**

**
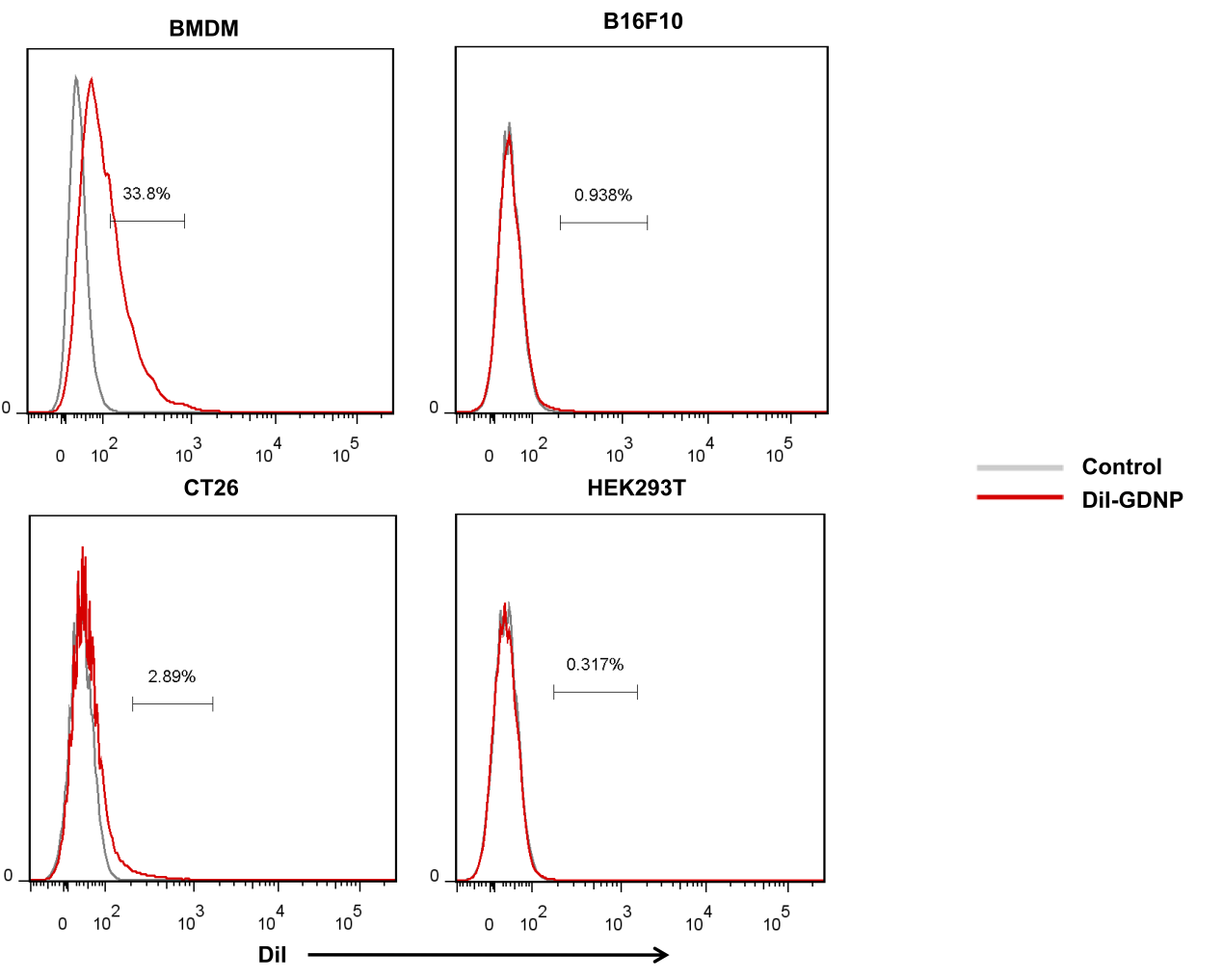

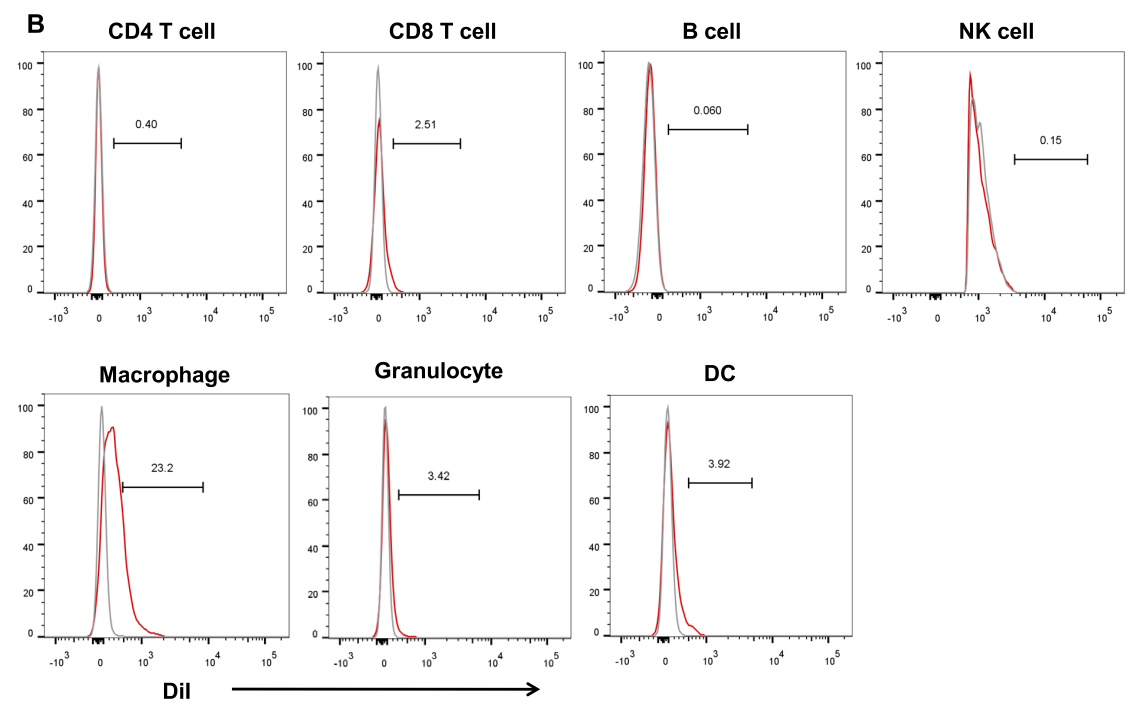
**

**d**

**Tumor**

**D**

**
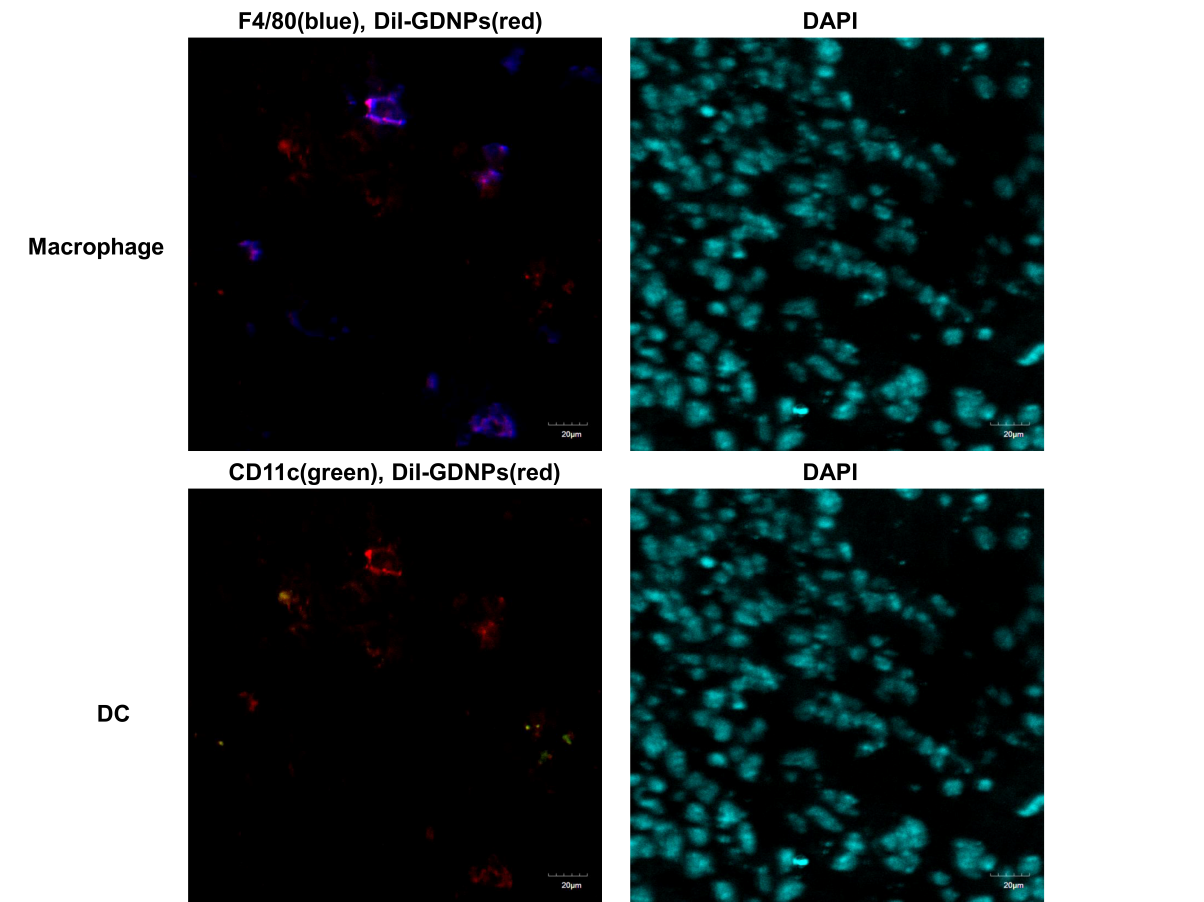
**

**F4/80**

**
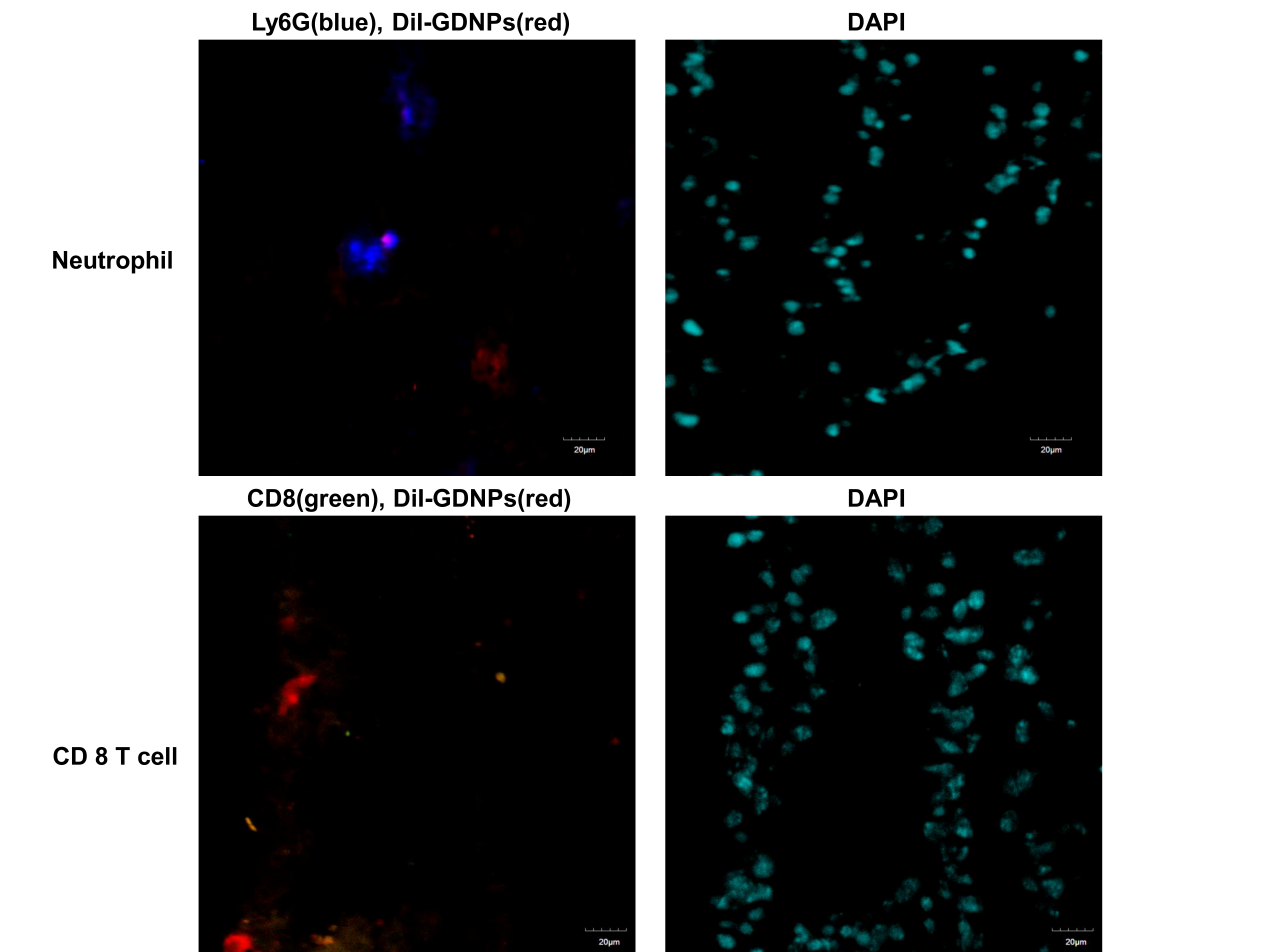
**

**DiI-GDNPs**

**F4/80**

**e**

**Merge**

**F4/80**

**DiI-GDNPs**

**
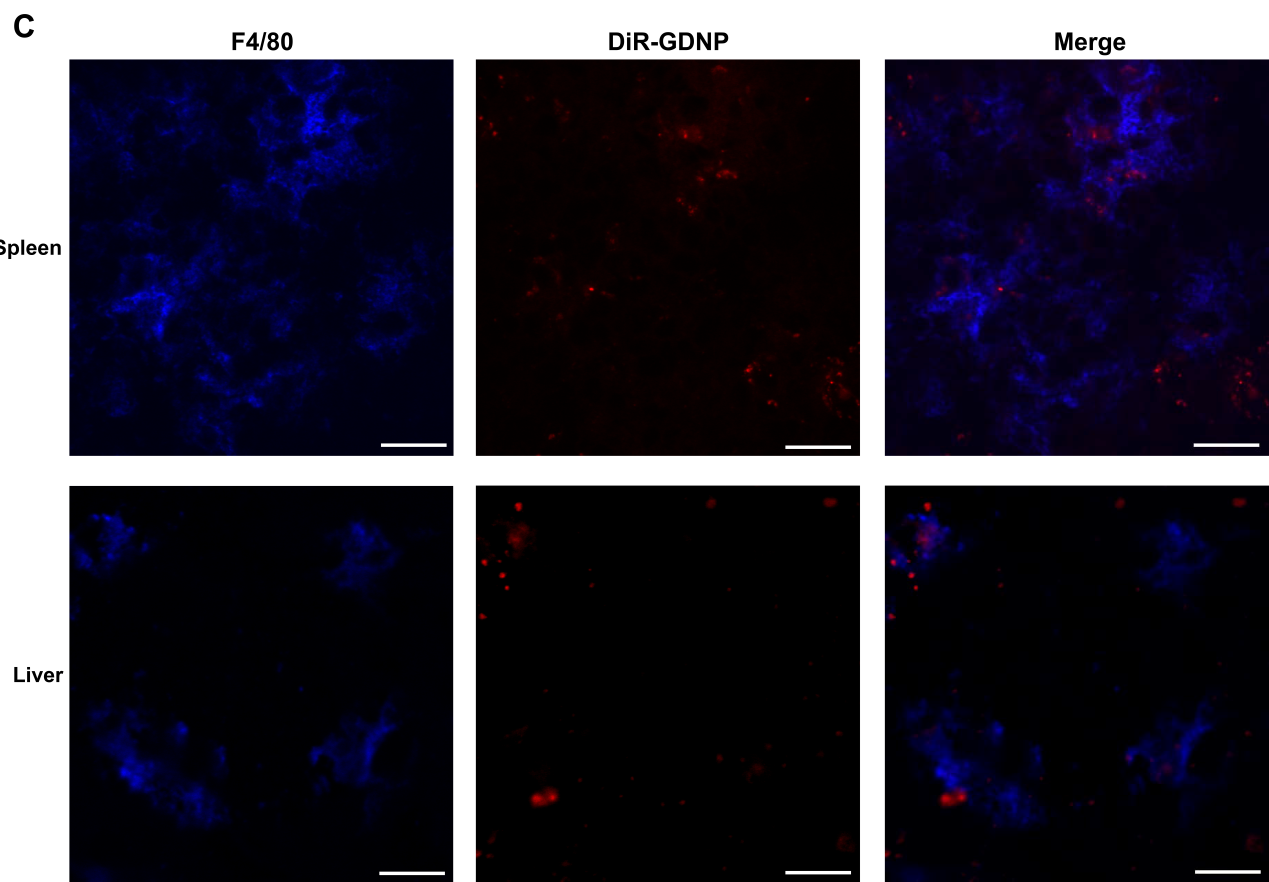
**

**Spleen**

**Liver**

**
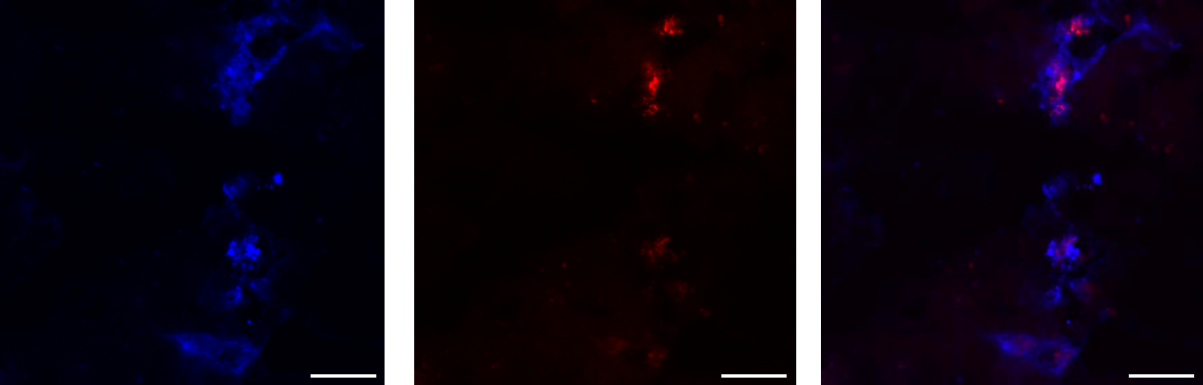
**

**Tumor**

**Figure S7.** GDNPs inhibit melanoma tumor growth *in vivo*. **a** Tumor samples per group obtained 21 days after implantation. **b** The percentages of DCs, B cells and granulocytes in total CD45^+^ TILs. **c** *In vivo* FACS quantitative analysis of main sets of TILs uptake of DiI-labelled GDNPs administrated i.p. injection. **d** Representative confocal fluorescence images showing the co-localization of DiI-GDNPs with macrophages, DCs, granulocytes or CD8^+^ T cells in the tumor of the melanoma-bearing mouse model following 14 days administration. (Scale bar = 20 μm). **e** Representative confocal fluorescence images showing the accumulation of DiI-GDNPs in liver, spleen and tumor of the melanoma-bearing mouse model following 14 days administration. (Scale bar = 20 μm).

**

Figure S8.** Mean tumor volume of subcutaneous B16F10 tumors in GDNPs versus PBS-treated mice with or without CD8^+^ T cell depletion by anti-CD8 antibody. The results represent the mean ± SEM (*n* = 5). Two-way ANOVA was used to compare results of different experimental groups for statistically significant difference (**P* < 0.05, *****P* < 0.0001).


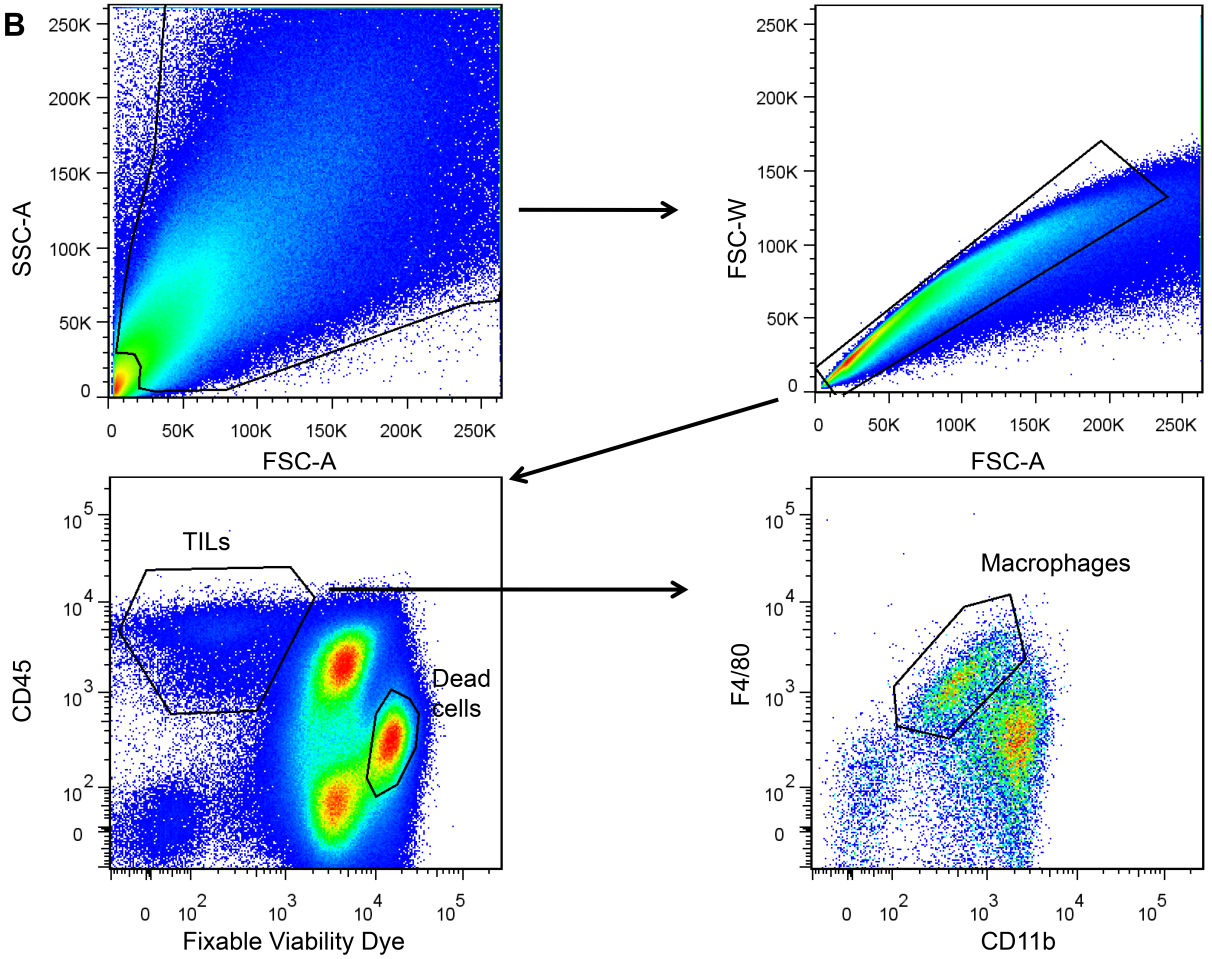


**Figure S9.** Flow cytometry gating strategy for polarization analysis of tumor associated macrophages (TAMs).

**Supplemental Methods**

**Preparation of cucumber and kiwi derived nanoparticles**

To prepare cucumber and kiwi derived nanoparticles, fresh cucumber and kiwi were purchased from the local market. The plants listed above were peeled and isolated according to the methods as described above. The protein concentration in the cucumber and kiwi derived nanoparticles were quantified using a BCA protein assay kit.

**Analysis of the composition of GDNPs**

The composition of GDNPs was analysed by Jingjie PTM Bio Co. Ltd. (Hangzhou, Zhejiang, China) for analysis. Briefly, the molecular composition of GDNPs was determined using a triple quadrupole mass spectrometer (Applied Biosystems Q-TRAP, Applied Biosystems, USA). In addition, the composition of proteins was determined using tandem mass spectrometry (MS/MS) on a Q ExactiveTM Plus system (Thermo Fisher Scientific, USA) coupled online to the Ultra-Performance Liquid Chromatography column UPLC. The details have been described in the online protocols.

A sensitive LC-MS method has been developed for determination of Rg3 in GDNPs. For quantitative analysis, the separation of the multi components was carried out using a Waters Quattro Micro (Waters, USA) liquid chromatography equipped with a quaternary pump, an online vacuum degasser, an autosampler, a thermostatic column compartment. All data collected were analyzed and processed using the Masslynx (Waters, USA). Chromatographic separation was performed on an Agilent C18 column ( 4.6 mm × 150 mm, 5 μm) using gradient elution of acetonitrile-0.1% formic acid in water with a flow rate of 0.4ml/min. The mass spectrometer was run in positive electrospray ionization (ESI＋) mode.

**Biocompatibility assays**

Cell viability was determined by MTT assay. Briefly, B16F10 cells, 4T1 cells and HEK293T cells were seeded in 96-well plates at a density of 1×10^4^/well and incubated with increased concentrations of GDNPs (0-30 μg/ml) for 48 h. After incubation, cell viability was measured using Varioskan Flash (Thermo Fisher Scientific, USA) at 490 nm. Untreated cells were used as a negative control. Age- and weight-matched healthy male C57BL/6 mice were injected intraperitoneally (i.p.) with GDNPs (250 μg per mouse) or PBS. The body weights of the mice were measured every 2 days. After two weeks, the mice were euthanized, and blood samples were collected from each mouse, followed by whole blood cell analyses and blood chemistry tests. To conduct histological analysis, the main organs were harvested, fixed in 4% buffered paraformaldehyde, then embedded, sliced, stained with haematoxylin & eosin (H&E), and observed under an optical microscope (Olympus, Japan).

**Cytokine microarray analysis**

To further confirm the production of M1- and M2-associated cytokines, cytokine profiling was performed on the supernatants collected as described above using RayBiotech Mouse Inflammation Antibody Array G-Series 1 and G-Series Human Cytokine Antibody Array 440 (RayBiotech, USA). The samples were detected by the Shanghai XY Biotech Company. The experimental procedure was carried out in accordance with the manufacturer’s instructions. Briefly, pre-coated antibody array membranes were incubated with coating buffer for 30 min. After that, the blocking buffer was decanted and replaced with sample dilutions. Membranes were incubated overnight at 4°C with shaking. The next day, the membranes were incubated with biotin-conjugated antibody for 2 h after washing. Mixture of biotin-conjugated antibodies was removed and Streptavidin-Fluor was added to each sub-array. The incubation chambers were covered with adhesive film and incubated for 2 h. After washing, signals were detected with a GenePix 4000B system (Axon Instruments, USA). GenePix Pro 6.0 software (Axon Instruments) was used for densitometric analysis of the spots. These values were normalized to the ratio of positive control values for each sample. Afterward, the total normalized fluorescence values of replicate spots were averaged and expressed as fold increase over the control samples.

**T cell suppression assay.**

The preparation of M2 macrophages was described previously. Splenocytes from naive mice were obtained by gently pressing the spleens between two sterile glass slides and ground through a 100-μm nylon filter strainer and washed thoroughly in PBS to generate a single-cell suspension. After RBC lysis, CD8^+^ cells were purified using anti-CD8 (Ly-2) microbeads (Miltenyi Biotech, Germany) according to the manufacturer’s protocol and labelled with 2 mM CFSE (Thermo Fisher Scientific, USA) in pre-warmed PBS for 15 min at 37 °C. The CFSE-labelled CD8^+^ T cells were then plated in complete RPMI media onto round bottom 48-well plates (1 × 10^5^ cells per well) coated with 200 ng/ml anti-CD3 (clone 145-2C11) and 250 ng/ml anti-CD28 (clone 37.51) antibodies (Thermo Fisher Scientific, USA). M2 macrophages with or without GDNPs were added in indicated ratios and plates were incubated at 37 °C. The T cell proliferation is determined by Cell Trace Violet dye dilution measured by flow cytometry (FACSAria II Flow Cytometer, BD Biosciences) after 48 h.

**Potential pathways utilized by GDNPs to enter macrophages**

To study the effect of inhibitors on GDNPs uptake, M2-like macrophages (5×10^5^/well) were cultured at 37°C in the presence of 5-(N,N-Dimethyl) amiloride hydrochloride (EIPA, macropinocytosis inhibitor, 10 μM) or LY294002 (phagocytosis inhibitor, 30 μM) for 1 h before the addition of DiI-labelled GDNPs, and culturing continued for an additional 12 h. Each of the samples was analyzed for internalization by confocal microscopy and FACS. And the surface markers of macrophages were detected by FACS as described previously.

**Apoptosis assay**

The Annexin V-PE/7-AAD apoptosis assay was used to quantify cell apoptosis *in vitro*. B16F10 cells (1×10^6^/well) were seeded in 12-well culture plates and cultured in the presence of medium from M0, M2 and M2 plus GDNPs for 24 h. After the incubation, cells were washed twice with cold PBS and then suspended in binding buffer at a concentration of 1×10^6^ cells/ml. Then, 100 μl of the cell suspension were mixed with 5 μl Annexin V-FITC and 5 μl 7-AAD (BD Biosciences, USA) and incubated at room temperature for 15 min in the dark. Finally, 400 μl of Annexin V binding buffer was added and the results were analysed by flow cytometry. Healthy cells were double negative for Annexin V and 7-AAD staining, early apoptotic cells were positive for Annexin V but negative for 7-AAD staining, necrotic cells were positive only for 7-AAD, and late apoptotic cells were double positive.

**Measurement of reactive oxygen species (ROS)**

M2-like macrophages (1x10^6^/well) were seeded into 6-well plates. Cells were either untreated or incubated with GDNPs (10 μg/ml) for 48 h at 37°C in a humidified atmosphere with 5% CO_2_. Total ROS levels in live cells were measured using a Total ROS kit as per the manufacturer’s instructions (Thermo Fisher Scientific, USA) and analysed by flow cytometry. Flow cytometry data were analysed using FlowJo, and median fluorescence intensity (MFI) was used to compare total ROS produced by macrophages. At least 2x10^4^ cellular events were analysed. For measuring superoxide levels, hydrogen peroxide in the culture media was measured by incubating culture media with a hydrogen peroxide colorimetric detection kit for 30 min at 25°C (Enzo Life Science, Switzerland) and measuring the resultant absorbance at 550 nm.

**Histology**

For immunofluorescence, tumor samples were embedded in Tissue-Tek OCT compound (Thermo Fisher Scientific, USA), snap frozen in liquid nitrogen and cut into 6-μm thick frozen sections using a cryostat. After overnight drying, the sections were fixed in ice-cold acetone and stored at -80°C. Before staining, sections were blocked with 1% bovine serum albumin (BSA)-PBS. Then, the sections were stained with appropriate dilutions of various combinations of the following fluorochrome-conjugated antibodies: anti-CD206 Alexa Fluor 488; anti-CD86 (Alexa Fluor 647; anti-F4/80 Alexa Fluor 594; anti-F4/80 Alexa Fluor 647; anti-CD11c FITC; anti-Ly6G APC; anti-CD8 FITC (BioLegend, USA), and the corresponding isotype controls were diluted in 1% BSA-PBS to the indicated concentrations and applied to the sections followed by overnight incubation at 4°C in the dark. Sections were washed three times with PBS for 5 min and mounted with ProLongGold with DAPI (Thermo Fisher Scientific, USA) and imaged with 40X magnification with an Olympus FV10i confocal microscope (Olympus, Japan), and the resultant digital images were analysed using the Olympus Fluoview software version 4.0b. Images of three nonoverlapping optical fields covering the surface of the tumor sections were captured. Image analysis was performed in ImageJ using the area measurement application.

The remaining tumor tissues were fixed in 4% buffered paraformaldehyde for 24 h, then embedded, sectioned at 5 μm thick sections using Leica RM 2235 (Leica, Germany) and mounted on adhesive glass slides. To conduct histological analysis, the sections were stained with haematoxylin & eosin (H&E) using standard procedures and observed under an optical microscope (Olympus, Japan).

**CD8^+^ Cell depletion studies.**

C57BL6 mice were depleted of CD8^+^ T cells by i.p. injection of anti-CD8 antibody (clone 169.4, BioXCell, USA) beginning 3 days before tumor implantation and continuing every 3 days for the duration of the study. Control mice were injected i.p. with the isotype control antibody (clone LTF-2, BioXCell, USA) according to the same dosing schedule. CD8^+^ T-cell-depleted and isotype control-treated mice were implanted with 2×10^5^ B16F10 cells subcutaneously on their right flanks. Seven days after implantation, animals were administered every four days with PBS or GDNPs (250 μg per mouse) and dosed every three days with 100 μg antibody, either isotype control or anti-CD8. Tumor measurements were taken every two days for up to 19 days after implantation.
